# Mortality, morbidity, and hospitalisations due to influenza lower respiratory tract infections, 2017: an analysis for the Global Burden of Disease Study 2017

**DOI:** 10.1016/S2213-2600(18)30496-X

**Published:** 2019-01

**Authors:** GBD 2017 Influenza Collaborators, Christopher E Troeger, Brigette F. Blacker, Ibrahim A. Khalil, Stephanie R M Zimsen, Samuel B. Albertson, Degu Abate, Jemal Abdela, Tara Ballav Adhikari, Sargis Aghasi Aghayan, Sutapa Agrawal, Alireza Ahmadi, Amani Nidhal Aichour, Ibtihel Aichour, Miloud Taki Eddine Aichour, Ayman Al-Eyadhy, Rajaa M Al-Raddadi, Fares Alahdab, Kefyalew Addis Alene, Syed Mohamed Aljunid, Nelson Alvis-Guzman, Nahla Hamed Anber, Mina Anjomshoa, Carl Abelardo T. Antonio, Olatunde Aremu, Hagos Tasew Atalay, Suleman Atique, Engi F. Attia, Euripide F G A Avokpaho, Ashish Awasthi, Arefeh Babazadeh, Hamid Badali, Alaa Badawi, Joseph Adel Mattar Banoub, Aleksandra Barac, Quique Bassat, Neeraj Bedi, Abate Bekele Belachew, Derrick A. Bennett, Krittika Bhattacharyya, Zulfiqar A Bhutta, Ali Bijani, Félix Carvalho, Carlos A Castañeda-Orjuela, Devasahayam J Christopher, Lalit Dandona, Rakhi Dandona, Anh Kim Dang, Ahmad Daryani, Meaza Girma Degefa, Feleke Mekonnen Demeke, Meghnath Dhimal, Shirin Djalalinia, David Teye Doku, Manisha Dubey, Eleonora Dubljanin, Eyasu Ejeta Duken, Dumessa Edessa, Maysaa El Sayed Zaki, Hamed Fakhim, Eduarda Fernandes, Florian Fischer, Luisa Sorio Flor, Kyle J. Foreman, Teklu Gebrehiwo Gebremichael, Demeke Geremew, Keyghobad Ghadiri, Alessandra C Goulart, Jingwen Guo, Giang Hai Ha, Gessessew Bugssa Hailu, Arvin Haj-Mirzaian, Arya Haj-Mirzaian, Samer Hamidi, Hamid Yimam Hassen, Chi Linh Hoang, Nobuyuki Horita, Mihaela Hostiuc, Seyed Sina Naghibi Irvani, Ravi Prakash Jha, Jost B. Jonas, Amaha Kahsay, André Karch, Amir Kasaeian, Tesfaye Dessale Kassa, Adane Teshome Kefale, Yousef Saleh Khader, Ejaz Ahmad Khan, Gulfaraz Khan, Md Nuruzzaman Khan, Young-Ho Khang, Abdullah T Khoja, Jagdish Khubchandani, Ruth W Kimokoti, Adnan Kisa, Luke D Knibbs, Sonali Kochhar, Soewarta Kosen, Parvaiz A Koul, Ai Koyanagi, Barthelemy Kuate Defo, G Anil Kumar, Dharmesh Kumar Lal, Prabhat Lamichhane, Cheru Tesema Leshargie, Miriam Levi, Shanshan Li, Erlyn Rachelle King Macarayan, Marek Majdan, Varshil Mehta, Addisu Melese, Ziad A Memish, Desalegn Tadese Mengistu, Tuomo J Meretoja, Tomislav Mestrovic, Bartosz Miazgowski, George J Milne, Branko Milosevic, Erkin M Mirrakhimov, Babak Moazen, Karzan Abdulmuhsin Mohammad, Shafiu Mohammed, Lorenzo Monasta, Lidia Morawska, Seyyed Meysam Mousavi, Oumer Sada S Muhammed, Srinivas Murthy, Ghulam Mustafa, Aliya Naheed, Huong Lan Thi Nguyen, Nam Ba Nguyen, Son Hoang Nguyen, Trang Huyen Nguyen, Muhammad Imran Nisar, Molly R Nixon, Felix Akpojene Ogbo, Andrew T Olagunju, Tinuke O Olagunju, Eyal Oren, Justin R Ortiz, Mahesh P A, Smita Pakhale, Shanti Patel, Deepak Paudel, David M Pigott, Maarten J Postma, Mostafa Qorbani, Anwar Rafay, Alireza Rafiei, Vafa Rahimi-Movaghar, Rajesh Kumar Rai, Mohammad Sadegh Rezai, Nicholas L S Roberts, Luca Ronfani, Salvatore Rubino, Saeed Safari, Saeid Safiri, Zikria Saleem, Evanson Zondani Sambala, Abdallah M. Samy, Milena M Santric Milicevic, Benn Sartorius, Shahabeddin Sarvi, Miloje Savic, Monika Sawhney, Sonia Saxena, Seyedmojtaba Seyedmousavi, Masood Ali Shaikh, Mehdi Sharif, Aziz Sheikh, Mika Shigematsu, David L Smith, Ranjani Somayaji, Joan B Soriano, Chandrashekhar T Sreeramareddy, Mu'awiyyah Babale Sufiyan, Mohamad-Hani Temsah, Belay Tessema, Mebrahtu Teweldemedhin, Miguel Tortajada-Girbés, Bach Xuan Tran, Khanh Bao Tran, Afewerki Gebremeskel Tsadik, Kingsley Nnanna Ukwaja, Irfan Ullah, Tommi Juhani Vasankari, Giang Thu Vu, Fiseha Wadilo Wada, Yasir Waheed, T. Eoin West, Charles Shey Wiysonge, Ebrahim M Yimer, Naohiro Yonemoto, Zoubida Zaidi, Theo Vos, Stephen S Lim, Christopher J L Murray, Ali H Mokdad, Simon I. Hay, Robert C Reiner

## Abstract

**Background:**

Although the burden of influenza is often discussed in the context of historical pandemics and the threat of future pandemics, every year a substantial burden of lower respiratory tract infections (LRTIs) and other respiratory conditions (like chronic obstructive pulmonary disease) are attributable to seasonal influenza. The Global Burden of Disease Study (GBD) 2017 is a systematic scientific effort to quantify the health loss associated with a comprehensive set of diseases and disabilities. In this Article, we focus on LRTIs that can be attributed to influenza.

**Methods:**

We modelled the LRTI incidence, hospitalisations, and mortality attributable to influenza for every country and selected subnational locations by age and year from 1990 to 2017 as part of GBD 2017. We used a counterfactual approach that first estimated the LRTI incidence, hospitalisations, and mortality and then attributed a fraction of those outcomes to influenza.

**Findings:**

Influenza LRTI was responsible for an estimated 145 000 (95% uncertainty interval [UI] 99 000–200 000) deaths among all ages in 2017. The influenza LRTI mortality rate was highest among adults older than 70 years (16·4 deaths per 100 000 [95% UI 11·6–21·9]), and the highest rate among all ages was in eastern Europe (5·2 per 100 000 population [95% UI 3·5–7·2]). We estimated that influenza LRTIs accounted for 9 459 000 (95% UI 3 709 000–22 935 000) hospitalisations due to LRTIs and 81 536 000 hospital days (24 330 000–259 851 000). We estimated that 11·5% (95% UI 10·0–12·9) of LRTI episodes were attributable to influenza, corresponding to 54 481 000 (38 465 000–73 864 000) episodes and 8 172 000 severe episodes (5 000 000–13 296 000).

**Interpretation:**

This comprehensive assessment of the burden of influenza LRTIs shows the substantial annual effect of influenza on global health. Although preparedness planning will be important for potential pandemics, health loss due to seasonal influenza LRTIs should not be overlooked, and vaccine use should be considered. Efforts to improve influenza prevention measures are needed.

**Funding:**

Bill & Melinda Gates Foundation.

## Introduction

In 1918, an influenza pandemic killed an estimated 20–50 million people,^1^, ^2^, ^3^ more than the number that died in World War 1. Today, seasonal influenza remains a substantial contributor to the growing number of cases of lower respiratory tract infections (LRTI) worldwide.^4^ Research is underway to clarify the pandemic potential of influenza.^5^, ^6^, ^7^ Such efforts have focused on understanding the risk factors predictive of pandemic potential, modelling disease transmission to inform preparedness,^6^ and identifying strategies to interrupt or mitigate pandemics.^8^ However, the sum of seasonal influenza deaths in the past 100 years is likely to exceed deaths due to influenza pandemics, and seasonal influenza is responsible for substantial mortality, disability, and economic disruption. Appropriate efforts to decrease this burden require timely and reliable estimates of the full spectrum of disease.

**Figure 1:**
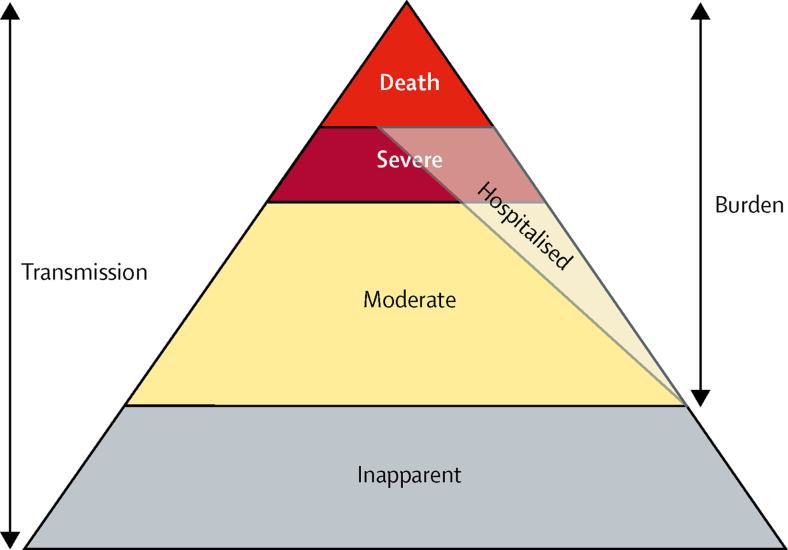
Conceptual diagram of the influenza LRTI burden pyramid This diagram shows the spectrum of influenza LRTI. We presents estimates of moderate and severe influenza LRTIs (of which some fraction [modelled independently] is hospitalised), and mortality due to influenza LRTI. We did not estimate inapparent infection, which could be important for understanding the transmission dynamics of influenza LRTIs but does not account for a measurable burden of disease. LRTI=lower respiratory tract infection.

The construction of a pyramid of influenza disease burden would include metrics describing the spectrum of disease, including the incidence of moderate and severe LRTIs, hospitalisations, and deaths (figure 1). By contrast, a transmission pyramid could also include asymptomatic infections, which, by definition, do not have a disease burden but might be crucial to the understanding of influenza transmission dynamics. This conceptualisation could enable public health officials, health-care providers, and policy makers to use available data to focus on any point of the pyramid and develop a comprehensive sense of influenza burden. The burden of influenza LRTI is difficult to quantify for various reasons related to diagnosis of LRTIs, detection of influenza, and data availability in many setting. Furthermore, there is a dearth of information available about the burden of influenza as an aetiology of LRTIs,^9^ and a full perspective of the health loss associated with influenza LRTIs at the population level is important to understand the burden and develop surveillance and intervention programmes.

The Global Burden of Disease Study (GBD) 2017 is a systematic scientific effort to produce comprehensive and comparable estimates of the burden of disease across causes of death and disability. In this Article, we seek to quantify the burden of influenza LRTIs by using a counterfactual approach to estimate LRTIs caused by influenza and build on previous descriptions of LRTIs in the GBD.^4^, ^10^ LRTIs are the leading cause of infectious disease mortality worldwide, and cause more deaths than tuberculosis and HIV combined. In 2016, they were responsible for more than 2 500 000 deaths and were the fifth-leading incident infectious disease globally.^4^ Within the GBD framework, influenza is considered a causal aetiology only for LRTIs and it is estimated as a subset of the overall LRTI burden. In this Article, we describe the global incidence of influenza LRTIs, rates of hospitalisation associated with influenza LRTIs, and the number of deaths due to influenza LRTIs across time, geographical regions and age groups.

**Research in context**

**Evidence before the study**

The burden of influenza has frequently been described in geographical or age-specific subpopulations, and several studies have focused on pandemic H1N1 or syndromic definitions of influenza, such as influenza-like illness. Several studies have sought to describe mortality and morbidity associated with influenza, including a publication led by the US Centers for Disease Control and Prevention and WHO that estimated 298 000–646 000 seasonal influenza-associated respiratory deaths globally in 2015. Previous iterations of the Global Burden of Disease Study (GBD) reported mortality from influenza-attributable lower respiratory tract infection (LRTI) globally, with an estimated 58 000 deaths (95% uncertainty interval 44 000–74 000) in 2016. An analysis to produce a comprehensive description of influenza LRTIs that covers the full range of the disease by age group and geographical region, and over time, has not previously been done.

**Added value of this study**

We estimated the influenza-attributable burden of LRTIs, including estimates of incidence, hospitalisation, and deaths for every country globally, for both sexes, and for all ages, for 2017. To the best of our knowledge, no other study has produced estimates for such specific demographic categories. We leveraged the statistical methods developed for the GBD to produce internally consistent estimates of LRTI morbidity and mortality, and applied a counterfactual strategy to establish the fraction of LRTI burden that was directly caused by influenza. The strength of these methods is that our results are interpretable as a preventable burden if influenza transmission were reduced or eliminated. Such findings provide detailed evidence about where influenza LRTI burden is greatest by age and geography, and about the potential health effects of efforts to reduce influenza transmission.

**Implications of all the available evidence**

Our estimates of influenza LRTI suggest that the burden is not uniform across regions or by age, and that locations and age groups with the highest underlying rate of LRTI have the highest influenza LRTI burden. We suggest that interventions that affect influenza transmission, such as vaccination, should be combined with interventions that reduce LRTI risk, such as improvement of air quality, to reduce the overall burden of influenza LRTI.

## Methods

### Summary

The GBD quantifies four pathogens as causative aetiologies of LRTI, namely the influenza virus, respiratory syncytial virus, *Haemophilus influenzae* type b, and *Streptococcus pneumoniae*. These four pathogens were identified on the basis of expert opinion. A comprehensive description of LRTI modelling, including mortality and morbidity methods for all-cause LRTI, has been described elsewhere,^4^ and so here we focus on specific methods for influenza attribution in GBD 2017.^4^ Estimation of LRTIs attributable to influenza has two main components. First, we estimated the counterfactual attributable fraction of LRTIs that were due to influenza on the basis of modelled estimates produced by a Bayesian predictive model by age, sex, year, and geography. Second, we estimated the number of deaths and hospitalisations due to LRTIs and episodes of LRTIs by age, sex, year, and geography with Bayesian predictive models. The product of the LRTI mortality, episodes, and hospitalisations and the influenza attributable fraction was the estimated burden of influenza in this study. This study complies with the Guidelines for Accurate and Transparent Health Estimates Reporting (GATHER) statement.^11^

### Procedures

We used a counterfactual definition for influenza. LRTIs were defined as clinician-diagnosed pneumonia or bronchiolitis (appendix p 1).^4^ There is evidence of a causal association between influenza and LRTIs among children younger than 5 years when the influenza virus is detected by reverse transcriptase (RT) PCR of respiratory samples.^12^ On the basis of these data, we estimated the population attributable fraction (PAF) of LRTI episodes, deaths, and hospitalisations that were caused by influenza in all age groups. This approach was a counterfactual analysis to establish the contribution of influenza to LRTIs. In this analysis, the counterfactual estimated was the burden of LRTI that would exist in the absence of influenza—or, in other words, the burden of LRTI causally attributable to influenza. The attribution was based on the exposure and the risk of the outcome.

Data for influenza were obtained through a systematic review^4^ of scientific literature for the proportion of LRTIs that tested positive by PCR or ELISA for influenza, from studies published between Jan 1, 1990, and May 26, 2017. We excluded studies that focused only on 2009 pandemic H1N1 influenza and studies in which influenza-like illness was the outcome definition. Our final dataset included all data used in GBD 2016 in addition to those identified in our updated systematic review. The search string is provided in the appendix (p 3). We included studies that had a sample size of at least 100 people (to avoid potential biases associated with small denominators, and consistent with other aetiologies in GBD 2017), were at least 1 year in duration (to limit seasonal detection bias), and used a case definition of LRTI, pneumonia, or bronchiolitis. During our updated systematic review, we identified 595 studies, 75 of which met our inclusion criteria and were extracted. These 75 studies were added to the 153 data sources that were used in GBD 2016 and extracted according to the same inclusion and exclusion criteria. We did not include surveillance or administrative data because they do not typically include the proportion of LRTIs positive for influenza and because they are prone to reporting and testing biases.

Specifically, we sought to model the frequency of detection of influenza in LRTI episodes on the basis of an RT-PCR reference case definition. The frequency of detection was a modelled value with variation in age, sex, year, and geography from our model and was based on the distributions of the input data. In this model, we estimated the relative frequency of detection in hospitalised compared with non-hospitalised populations and assumed that this value was a proxy for fatal LRTI episodes. By contrast with a categorical approach, which would stop at this point, the counterfactual approach required quantification of the relative risk of LRTI in view of evidence of influenza in the nasopharynx or oropharynx, for which we used odds ratios (ORs) from a systematic review and meta-analysis of studies of children younger than 5 years.^12^ In the absence of available and reliable results in older children and adults, we assumed that this association was constant across age groups.

### Statistical analysis

We estimated three related PAFs for non-mutually exclusive influenza LRTI categories: a non-fatal PAF, a hospitalisation PAF, and a fatal PAF (appendix p 4). The non-fatal PAF (appendix p 4) was the simplest.

$$\text{Non}-\text{fatal PAF}=\text{frequency}\times \left(1-\frac{1}{\text{OR}}\right)$$

Frequency was the modelled proportion of LRTI episodes that test positive for influenza by PCR, which varied by age, sex, year, and geography, and OR was the odds of an LRTI episode in view of the presence of influenza in a respiratory tract sample (5·10 [95% CI 3·19–8·14]).^12^ To account for the previously described frequency of influenza detection in hospitalised compared with non-hospitalised LRTI episodes, using the equation for non-fatal PAF, we applied a constant scalar (ie, a hospital scalar) to establish the PAF for LRTI hospitalisations. This PAF was calculated by comparing the mean frequency of influenza detection in hospitalised sample populations with that in non-hospitalised sample populations in the proportion data from our literature review.

Finally, to account for the relative difference in the risk of mortality between bacterial and viral causes of LRTIs, using the equation for hospitalisation PAF, we applied a fatality scalar. We modelled the ratio of case fatality of viral-to-bacterial International Classification of Diseases-coded hospital admissions (appendix p 23) to calculate the fatality scalar, which was applied to establish the attribution of influenza for fatal LRTI outcomes. Hospital data from high-income and low-income countries were used in this analysis, and we estimated an age-specific curve for this relationship (appendix p 24).

The final number of LRTI episodes, deaths, and hospitalisations attributable to influenza was the product of the relevant PAF and the overall number of episodes, deaths, and hospitalisations for each country, year, age, and sex.

LRTI episodes, deaths, and hospitalisations were modelled independently of influenza attributable fractions. The mortality due to LRTIs was modelled with a Bayesian predictive ensemble modelling tool developed for the GBD called the Cause of Death Ensemble model (CODEm) which has been described in detail previously (appendix pp 1–2).^13^, ^14^ Briefly, CODEm uses a covariate selection algorithm to produce a wide array of sub-models, which are assessed on the basis of their out-of-sample predictive validity. The best-performing models then contribute relatively more to a final ensemble model of LRTI mortality. The input data for this model were vital registration data, verbal autopsy studies, and surveillance system records. The predictive validity of this model was assessed with out-of-sample statistics, and we judged the best-performing model to be the one with the best out-of-sample values for statistical fit.

The incidence of all LRTI episodes was modelled with a Bayesian meta-regression tool developed for the GBD called DisMod-MR 2.1 (appendix p 3).^15^ DisMod-MR 2.1 was designed to incorporate all available epidemiological data, to standardise these data so that they are comparable, and to develop estimates of disease burden by age, sex, year, and geography. Data for this model primarily came from population-representative surveys, inpatient and outpatient health-care utilisation records, and scientific literature. LRTIs in GBD are defined as either moderate or severe, and the proportion of severe LRTI episodes was established by a meta-analysis of the incidence of severe LRTIs—defined as infections that required inpatient admission or oxygen therapy, or that met the WHO Integrated Management of Childhood Infections definition (appendix p 3) of severe pneumonia—versus non-severe LRTIs in studies in which the incidence of both was reported.^4^ Modelling of severe influenza LRTIs and hospitalised influenza LRTIs was not done with the same data, and so were not independent from each other in this analysis.

The incidence of LRTI hospitalisations was also modelled with DisMod-MR 2.1. For this model, only inpatient utilisation data were included. These data were primarily from high-income countries such as the USA and western Europe countries, but data from Brazil, India, Indonesia, Kenya, Mexico, Nepal, the Philippines, Qatar, and Vietnam were also included (appendix pp 24–25). Covariates such as the total inpatient visits per person and the Healthcare Access and Quality Index^16^ were used to help account for variations in health-care availability and access. The duration in days of hospitalisation for viral LRTI episodes was based on a meta-analysis of studies in which this duration was reported.^4^

All modelled estimates for the GBD, including influenza attributable fractions, influenza LRTI incidence, hospitalisations, and deaths, were estimated for 195 countries and territories by sex and age group, from 1990 to 2017. The results presented are the mean values from a distribution of 1000 estimated observations (draws) for each modelled value or input parameter. 95% uncertainty intervals (UI) are reported as the 2·5th and 97·5th percentiles of the posterior distributions.

### Role of the funding source

The study sponsor had no role in study design; data collection, analysis, or interpretation; or writing of the report. The corresponding author had full access to all study data and had final responsibility for the decision to submit for publication.

## Results

**Figure 2:**
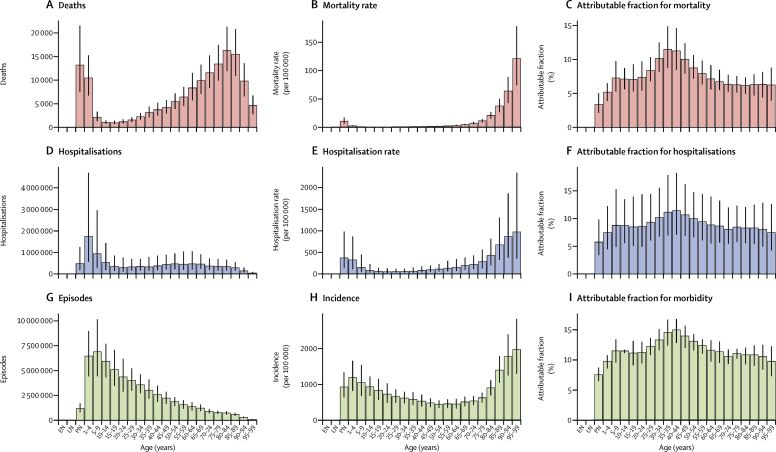
Age distribution of deaths attributed to influenza lower respiratory tract infections (A–C), hospitalisations attributed to influenza lower respiratory tract infections (D–F), and episodes of influenza lower respiratory tract infections (G–I) globally, 2017 Lower respiratory tract infections were not attributed to influenza in the early or late neonatal age groups. Error bars show 95% uncertainty intervals. EN=early neonatal (ie, 0–6 days). LN=late neonatal (7–27 days), PN=post neonatal.

**Figure 3:**
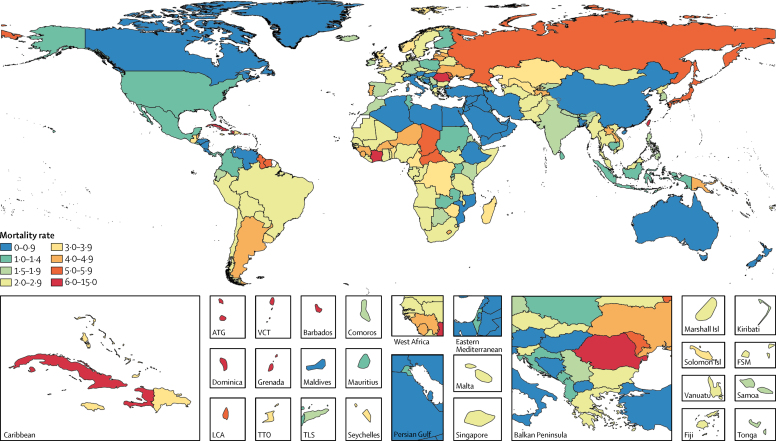
Influenza lower respiratory tract infection mortality rate per 100 000 for all ages, 2017 ATG=Antigua and Barbuda. FSM=Federated States of Micronesia. LCA=Saint Lucia. VCT=Saint Vincent and the Grenadines. TLS=Timor-Leste. TTO=Trinidad and Tobago. Isl=Islands.

We estimated that 5·6% (95% UI 4·3–7·1) of global LRTI deaths were attributable to influenza in 2017, which corresponded to 145 000 (98 000–200 000) deaths across all ages (table). Deaths attributable to influenza accounted for 0·26% (95% UI 0·2–0·32) of all deaths in 2017. The PAF was greater among adults older than 70 years (6·3% [95% UI 4·8–7·8) than among children younger than 5 years (2·9% [2·0–4·0]; figure 2; appendix p 28). The fraction of LRTI deaths that were attributable to influenza ranged from 1·9% (95% UI 1·6–2·2) in Mozambique to 23·7% (19·1–27·6) in Ukraine (appendix p 28). Most influenza LRTI deaths occurred among elderly people, with 71 000 deaths (95% UI 50 000–95 000) among adults older than 70 years (figure 2). Mortality rate was also highest in this age group (16·4 deaths per 100 000 [95% UI 11·6–21·9]). Among all ages, when looking at regional estimates, the highest estimated influenza LRTI mortality rates occurred in the Caribbean (5·5 per 100 000 [95% UI 3·6–7·7]) and eastern Europe (5·2 per 100 000 [3·5–7·2]) regions, and the highest mortality rate overall was in Taiwan (province of China; 12·1 per 100 000 [7·8–17·6]; figure 3; table). The estimated influenza LRTI mortality rate was lowest in the Australasia region (0·9 per 100 000 [95% UI 0·5–1·3), and Qatar was the country with the lowest rate (0·2 per 100 000 [0·1–0·4]; figure 3; table). Nearly a third of all influenza LRTI deaths occurred in India (26 000 [95% UI 16 000–37 000]), China (11 000 [7000–16 000]), and Russia (8000 [5000–11 000]; table). Between 1990 and 2017, the influenza LRTI mortality rate decreased by 29·5% among all ages (from 2·7 per 100 000 to 1·9 per 100 000). The rate of decline in this period was fastest among children younger than 5 years (67·8%) and slowest among adults older than 70 years (10·2%; data not shown).

**Table:** Influenza lower respiratory tract infection episodes, hospitalisations, and deaths among all ages by GBD country, region, and super-region, 2017

			**Deaths (95% UI)**	**Deaths per 100 000 (95% UI)**	**Hospitalisations (95% UI)**	**Hospitalisations per 100 000 (95% UI)**	**Episodes (95% UI)**	**Incidence per 100 000 (95% UI)**
**Global**			**145 000 (99 000 to 200 000)**	**1·9 (1·3 to 2·6)**	**9 459 000 (3 709 000 to 22 935 000)**	**123·8 (48·5 to 300·2)**	**54 481 000 (38 465 000 to 73 864 000)**	**713·1 (503·4 to 966·7)**
**Central Europe, eastern Europe, and central Asia**			**16 000 (11 000 to 23 000)**	**3·9 (2·7 to 5·4)**	**1 609 000 (660 000 to 3 731 000)**	**386·8 (158·6 to 897·0)**	**6 624 000 (4 796 000 to 8 752 000)**	**1592·6 (1153·0 to 2104·3)**
Central Asia			3000 (2**000** to 4000)	3·1 (2·0 to 4·4)	276 000 (110 000 to 656 000)	303·1 (120·5 to 721·6)	1 175 000 (867 000 to 1 531 000)	1292·3 (953·3 to 1683·5)
	Armenia		<1000 (<1000 to <1000)	2·6 (1·7 to 3·9)	11 000 (4000 to 27 000)	365·0 (134·5 to 902·1)	40 000 (28 000 to 54 000)	1331·2 (935·2 to 1798·8)
	Azerbaijan		<1000 (<1000 to <1000)	2·4 (1·3 to 4·1)	26 000 (9000 to 70 000)	252·9 (88·0 to 688·4)	109 000 (75 000 to 154 000)	1066·3 (733·5 to 1506·0)
	Georgia		<1000 (<1000 to <1000)	3·6 (2·2 to 5·4)	11 000 (4000 to 25 000)	292·2 (120·5 to 672·6)	47 000 (33 000 to 63 000)	1263·1 (889·4 to 1703·7)
	Kazakhstan		1000 (<1000 to <1000)	3·5 (2·2 to 5·4)	61 000 (22 000 to 156 000)	341·6 (124·1 to 872·2)	243 000 (170 000 to 330 000)	1355·2 (949·0 to 1842·2)
	Kyrgyzstan		<1000 (<1000 to <1000)	1·6 (0·9 to 2·5)	25 000 (9000 to 65 000)	398·4 (144·9 to 1026·3)	88 000 (61 000 to 121 000)	1377·3 (951·0 to 1892·5)
	Mongolia		<1000 (<1000 to <1000)	2·0 (1·1 to 3·5)	8000 (3000 to 21 000)	242·4 (84·6 to 635·1)	35 000 (24 000 to 47 000)	1068·0 (746·4 to 1443·8)
	Tajikistan		<1000 (<1000 to <1000)	4·1 (2·2 to 7·0)	25 000 (8000 to 69 000)	265·4 (88·7 to 748·7)	112 000 (76 000 to 161 000)	1211·0 (819·0 to 1736·5)
	Turkmenistan		<1000 (<1000 to <1000)	2·1 (1·2 to 3·5)	10 000 (4000 to 29 000)	208·4 (71·4 to 573·9)	47 000 (32 000 to 67 000)	942·1 (641·6 to 1343·7)
	Uzbekistan		1000 (1000 to 2000)	3·2 (1·9 to 5·1)	99 000 (35 000 to 262 000)	307·0 (107·0 to 813·5)	456 000 (315 000 to 626 000)	1413·1 (976·9 to 1940·9)
Central Europe			3000 (2000 to 4000)	2·4 (1·7 to 3·3)	139 000 (60 000 to 312 000)	121·4 (52·1 to 272·0)	411 000 (289 000 to 560 000)	358·0 (251·8 to 488·2)
	Albania		<1000 (<1000 to <1000)	1·2 (0·6 to 2·0)	3000 (1000 to 9000)	116·0 (39·9 to 309·1)	10 000 (7000 to 14 000)	365·4 (251·8 to 503·4)
	Bosnia and Herzegovina		<1000 (<1000 to <1000)	1·0 (0·6 to 1·7)	4000 (1000 to 9000)	106·3 (37·1 to 278·8)	11 000 (8000 to 16 000)	338·1 (234·1 to 463·7)
	Bulgaria		<1000 (<1000 to <1000)	2·1 (1·3 to 3·2)	8000 (3000 to 21 000)	116·3 (42·1 to 293·6)	24 000 (17 000 to 32 000)	335·5 (234·4 to 456·6)
	Croatia		<1000 (<1000 to <1000)	1·4 (0·9 to 2·1)	4000 (2000 to 9000)	96·0 (42·1 to 213·7)	11 000 (7000 to 15 000)	251·3 (175·1 to 349·8)
	Czech Republic		<1000 (<1000 to <1000)	2·9 (1·8 to 4·2)	11 000 (5000 to 25 000)	102·9 (42·9 to 235·7)	32 000 (22 000 to 44 000)	303·5 (212·4 to 412·5)
	Hungary		<1000 (<1000 to <1000)	1·0 (0·6 to 1·4)	9000 (3000 to 25 000)	95·7 (33·5 to 253·3)	30 000 (21 000 to 42 000)	312·9 (215·6 to 430·4)
	Macedonia		<1000 (<1000 to <1000)	0·7 (0·4 to 1·2)	2000 (1000 to 7000)	112·1 (37·9 to 312·4)	8000 (6000 to 12 000)	381·8 (259·6 to 546·0)
	Montenegro		<1000 (<1000 to <1000)	1·2 (0·6 to 1·9)	1000 (<1000 to 2000)	126·1 (42·7 to 348·3)	3000 (2000 to 4000)	409·0 (277·0 to 586·3)
	Poland		1000 (<1000 to 1000)	1·4 (0·9 to 2·0)	21 000 (9000 to 49 000)	55·7 (22·8 to 127·8)	57 000 (39 000 to 77 000)	147·2 (101·7 to 200·7)
	Romania		1000 (1000 to 2000)	6·1 (4·0 to 8·9)	65 000 (28 000 to 147 000)	335·0 (141·9 to 756·5)	174 000 (121 000 to 235 000)	893·6 (624·5 to 1209·3)
	Serbia		<1000 (<1000 to <1000)	1·6 (0·9 to 2·5)	6000 (2000 to 14 000)	65·8 (25·4 to 161·4)	24 000 (17 000 to 33 000)	273·7 (189·4 to 375·5)
	Slovakia		<1000 (<1000 to <1000)	2·9 (1·8 to 4·5)	5000 (2000 to 13 000)	101·2 (40·0 to 244·3)	17 000 (12 000 to 24 000)	320·4 (222·6 to 438·0)
	Slovenia		<1000 (<1000 to <1000)	2·6 (1·7 to 3·9)	3000 (1000 to 6000)	123·2 (49·2 to 283·1)	6000 (4000 to 8000)	297·9 (207·1 to 406·1)
Eastern Europe			11 000 (7000 to 15 000)	5·2 (3·5 to 7·2)	1 027 000 (391 000 to 2 532 000)	488·7 (185·9 to 1204·6)	5 043 000 (3 610 000 to 6 738 000)	2399·3 (1717·2 to 3205·6)
	Belarus		<1000 (<1000 to <1000)	2·6 (1·5 to 4·1)	46 000 (15 000 to 127 000)	481·5 (161·1 to 1341·4)	232 000 (158 000 to 328 000)	2440·6 (1665·6 to 3460·9)
	Estonia		<1000 (<1000 to <1000)	3·3 (2·0 to 5·1)	6000 (2000 to 16 000)	466·6 (166·9 to 1205·1)	28 000 (20 000 to 38 000)	2151·3 (1512·7 to 2909·9)
	Latvia		<1000 (<1000 to <1000)	4·0 (2·4 to 6·1)	8000 (3000-20 000)	425·2 (167·8 to 1021·9)	44 000 (31 000 to 59 000)	2237·4 (1576·0 to 3021·9)
	Lithuania		<1000 (<1000 to <1000)	4·9 (3·1 to 7·4)	16 000 (6000-38 000)	560·7 (227·2 to 1351·7)	71 000 (49 000 to 99 000)	2489·6 (1728·2 to 3469·3)
	Moldova		<1000 (<1000 to <1000)	5·3 (3·4 to 7·9)	17 000 (6000-44 000)	451·7 (161·2 to 1192·0)	89 000 (63 000 to 121 000)	2395·6 (1687·7 to 3240·7)
	Russia		8000 (5000 to 11 000)	5·5 (3·7 to 7·6)	723 000 (268 000 to 1 815 000)	494·4 (183·6 to 1241·6)	3 504 000 (2 454 000 to 4 757 000)	2397·0 (1678·9 to 3253·9)
	Ukraine		2000 (1000 to 3000)	4·9 (3·1 to 7·3)	211 000 (75 000 to 550 000)	472·5 (168·9 to 1229·7)	1 076 000 (756 000 to 1 456 000)	2407·4 (1691·5 to 3258·0)
**High income**			**24 000 (17 000 to 32 000)**	**2·2 (1·5 to 2·9)**	**821 000 (366 000 to 1 725 000)**	**76·4 (34·1 to 160·5)**	**2 269 000 (1 668 000 to 2 963 000)**	**211·1 (155·1 to 275·6)**
Australasia			<1000 (<1000 to <1000)	0·9 (0·5 to 1·3)	11 000 (4000 to 27 000)	39·9 (15·1 to 95·9)	39 000 (27 000 to 53 000)	135·9 (94·2 to 185·7)
	Australia		<1000 (<1000 to <1000)	0·9 (0·5 to 1·3)	9000 (3000 to 23 000)	38·3 (14·0 to 94·1)	30 000 (21 000 to 41 000)	125·2 (86·6 to 171·7)
	New Zealand		<1000 (<1000 to <1000)	0·8 (0·5 to 1·2)	2000 (1000 to 5000)	47·2 (18·8 to 112·0)	9000 (6000 to 12 000)	193·1 (132·2 to 267·2)
High-income Asia Pacific			8000 (5000 to 10 000)	4·0 (2·7 to 5·6)	157 000 (64 000 to 357 000)	84·0 (34·4 to 190·9)	273 000 (192 000 to 370 000)	146·0 (102·8 to 197·6)
	Brunei		<1000 (<1000 to <1000)	1·3 (0·8 to 2·1)	<1000 (<1000 to 1000)	102·6 (35·0 to 262·2)	1000 (1000 to 1000)	173·7 (118·3 to 241·6)
	Japan		7000 (4000 to 9000)	5·1 (3·5 to 7·0)	104 000 (43 000 to 236 000)	80·9 (33·5 to 184·1)	182 000 (127 000 to 248 000)	141·5 (98·7 to 193·0)
	South Korea		1000 (1000 to 1000)	1·6 (1·0 to 2·3)	44 000 (16 000 to 108 000)	83·1 (29·9 to 206·0)	75 000 (52 000 to 101 000)	141·6 (99·3 to 192·5)
	Singapore		<1000 (<1000 to <1000)	3·0 (2·0 to 4·3)	10 000 (4000 to 22 000)	171·0 (65·2 to 396·8)	16 000 (11 000 to 22 000)	290·0 (204·8 to 390·4)
High-income North America			4000 (3000 to 6000)	1·1 (0·7 to 1·5)	195 000 (80 000 to 444 000)	54·0 (22·2 to 123·1)	1 014 000 (711 000 to 1 376 000)	280·9 (197·2 to 381·2)
	Canada		<1000 (<1000 to <1000)	0·8 (0·5 to 1·2)	16 000 (6000 to 38 000)	44·2 (16·6 to 106·7)	78 000 (54 000 to 107 000)	216·8 (150·6 to 296·0)
	Greenland		<1000 (<1000 to <1000)	0·9 (0·5 to 1·5)	<1000 (<1000 to <1000)	48·5 (17·6 to 121·5)	<1000 (<1000 to <1000)	277·6 (192·5 to 378·8)
	USA		4000 (2000 to 5000)	1·1 (0·8 to 1·6)	179 000 (73 000 to 407 000)	55·0 (22·5 to 125·4)	935 000 (656 000 to 1 272 000)	287·9 (201·8 to 391·7)
Southern Latin America			3000 (2000 to 4000)	3·9 (2·7 to 5·5)	78 000 (32 000 to 182 000)	119·4 (48·7 to 277·1)	341 000 (251 000 to 448 000)	519·6 (381·9 to 682·4)
	Argentina		2000 (1000 to 3000)	4·4 (2·8 to 6·4)	46 000 (17 000 to 117 000)	103·6 (37·7 to 264·0)	186 000 (127 000 to 258 000)	419·7 (288·0 to 582·0)
	Chile		1000 (0 to 1000)	2·9 (1·8 to 4·4)	28 000 (11 000 to 68 000)	153·8 (60·7 to 377·0)	138 000 (93 000 to 198 000)	768·5 (517·9 to 1102·3)
	Uruguay		<1000 (<1000 to <1000)	3·7 (2·3 to 5·6)	4000 (2000 to 11 000)	125·4 (46·4 to 307·7)	17 000 (12 000 to 23 000)	491·3 (342·0 to 671·6)
Western Europe			9000 (7000 to 12 000)	2·1 (1·5 to 2·8)	260 000 (119 000 to 529 000)	59·9 (27·5 to 122·1)	595 000 (453 000 to 755 000)	137·5 (104·7 to 174·4)
	Andorra		<1000 (<1000 to <1000)	2·4 (1·3 to 4·0)	<1000 (<1000 to <1000)	52·5 (19·6 to 129·7)	<1000 (<1000 to <1000)	132·4 (94·3 to 180·6)
	Austria		<1000 (<1000 to <1000)	0·7 (0·5 to 1·1)	5000 (2000 to 11 000)	56·4 (23·4 to 123·8)	11 000 (8000 to 15 000)	124·9 (88·2 to 169·2)
	Belgium		<1000 (<1000 to 1000)	3·2 (2·0 to 4·8)	10 000 (4000 to 22 000)	86·6 (36·7 to 190·6)	19 000 (14 000 to 27 000)	171·6 (120·4 to 234·9)
	Cyprus		<1000 (<1000 to <1000)	0·9 (0·5 to 1·6)	<1000 (<1000 to 1000)	27·6 (11·2 to 65·0)	1000 (1000 to 2000)	90·1 (63·9 to 121·7)
	Denmark		<1000 (<1000 to <1000))	2·7 (1·7 to 4·0)	4000 (1000 to 8000)	62·4 (24·6 to 143·5)	8000 (6000 to 11 000)	137·3 (97·6 to 184·2)
	Finland		<1000 (<1000 to <1000)	1·3 (0·8 to 2·0)	5000 (2000 to 11 000)	91·0 (36·6 to 204·6)	11 000 (7000 to 14 000)	190·8 (134·3 to 259·3)
	France		1000 (1000 to 2000)	2·2 (1·4 to 3·3)	36 000 (14 000 to 88 000)	55·2 (20·8 to 133·8)	88 000 (62 000 to 120 000)	134·6 (95·1 to 182·0)
	Germany		1000 (1000 to 2000)	1·7 (1·0 to 2·5)	49 000 (20 000 to 112 000)	58·4 (23·7 to 134·0)	101 000 (72 000 to 137 000)	121·6 (86·5 to 164·9)
	Greece		<1000 (<1000 to <1000)	2·7 (1·7 to 4·0)	6000 (2000 to 15 000)	60·9 (23·4 to 144·0)	15 000 (11 000 to 20 000)	142·7 (101·9 to 190·4)
	Iceland		<1000 (<1000 to <1000)	1·8 (1·2 to 2·8)	<1000 (<1000 to <1000)	48·3 (19·0 to 114·7)	<1000 (<1000 to 1000)	124·7 (88·0 to 168·7)
	Ireland		<1000 (<1000 to <1000)	1·8 (1·1 to 2·7)	2000 (1000 to 5000)	42·4 (15·9 to 104·0)	6000 (4000 to 7000)	113·5 (80·2 to 153·4)
	Israel		<1000 (<1000 to <1000)	1·3 (0·8 to 2·0)	3000 (1000 to 7000)	31·7 (11·7 to 80·9)	7000 (5000 to 10 000)	83·2 (57·3 to 117·2)
	Italy		<1000 (<1000 to 1000)	0·8 (0·5 to 1·2)	17 000 (7000 to 37 000)	28·2 (11·7 to 61·7)	38 000 (27 000 to 52 000)	63·4 (44·5 to 85·1)
	Luxembourg		<1000 (<1000 to <1000)	1·6 (1·0 to 2·5)	<1000 (<1000 to 1000)	48·6 (19·4 to 111·5)	1000 (1000 to 1000)	120·7 (85·6 to 162·7)
	Malta		<1000 (<1000 to <1000)	2·6 (1·7 to 3·9)	<1000 (<1000 to 1000)	59·0 (23·6 to 136·6)	1000 (1000 to 1000)	143·7 (102·4 to 192·8)
	Netherlands		<1000 (<1000 to 1000)	2·3 (1·5 to 3·4)	8000 (3000 to 20 000)	48·1 (18·7 to 114·7)	19 000 (14 000 to 26 000)	114·5 (81·7 to 155·3)
	Norway		<1000 (<1000 to <1000)	3·6 (2·4 to 5·1)	9000 (4000 to 20 000)	172·4 (72·4 to 378·8)	26 000 (18 000 to 35 000)	489·8 (341·6 to 668·2)
	Portugal		<1000 (<1000 to 1000)	4·4 (2·8 to 6·4)	5000 (2000 to 11 000)	44·4 (17·5 to 104·7)	17 000 (12 000 to 22 000)	155·2 (111·0 to 206·7)
	Spain		1000 (<1000 to 1000)	1·6 (1·0 to 2·3)	17 000 (7000 to 41 000)	37·7 (14·4 to 89·2)	42 000 (30 000 to 56 000)	91·1 (65·0 to 120·7)
	Sweden		<1000 (<1000 to <1000)	2·3 (1·4 to 3·4)	7000 (3000 to 18 000)	74·0 (27·8 to 179·8)	17 000 (12 000 to 23 000)	167·4 (116·7 to 229·1)
	Switzerland		<1000 (<1000 to <1000)	2·4 (1·5 to 3·6)	8000 (3000 to 17 000)	88·2 (36·4 to 198·4)	18 000 (13 000 to 24 000)	206·6 (145·8 to 281·5)
	UK		2000 (2000 to 3000)	3·7 (2·5 to 5·2)	67 000 (30 000 to 138 000)	100·7 (45·6 to 207·6)	148 000 (105 000 to 199 000)	222·4 (158·1 to 297·9)
		England	2000 (1000 to 3000)	3·8 (2·5 to 5·2)	61 000 (28 000 to 125 000)	108·8 (49·6 to 222·6)	133 000 (94 000 to 179 000)	237·4 (168·2 to 319·3)
		Northern Ireland	<1000 (<1000 to <1000)	3·4 (2·1 to 5·1)	1000 (<1000 to 3000)	57·0 (21·8 to 137·1)	3000 (2000 to 4000)	139·9 (99·3 to 188·0)
		Scotland	<1000 (<1000 to <1000)	3·4 (2·1 to 5·1)	3000 (1000 to 7000)	56·0 (21·6 to 133·1)	7000 (5000 to 10 000)	132·5 (94·6 to 177·0)
		Wales	<1000 (<1000 to <1000)	4·5 (2·9 to 6·7)	2000 (1000 to 5000)	71·1 (27·5 to 166·4)	5000 (4000 to 7000)	163·0 (116·2 to 217·7)
**Latin America and Caribbean**			**13 000 (9 000 to 18 000)**	**2·3 (1·6 to 3·2)**	**434 000 (177 000 to 1 029 000)**	**74·6 (30·5 to 176·8)**	**2 664 000 (1 871 000 to 3 628 000)**	**457·7 (321·6 to 623·4)**
Andean Latin America			1000 (1000 to 2000)	2·4 (1·5 to 3·5)	22 000 (8000 to 55 000)	35·6 (13·4 to 89·0)	427 000 (293 000 to 591 000)	694·6 (477·1 to 961·4)
	Bolivia		<1000 (<1000 to <1000)	2·3 (1·2 to 3·9)	4000 (1000 to 10 000)	30·6 (10·2 to 84·4)	72 000 (49 000 to 100 000)	622·1 (426·2 to 863·1)
	Ecuador		<1000 (<1000 to <1000)	1·7 (1·0 to 2·5)	6000 (2000 to 15 000)	36·5 (14·6 to 87·9)	125 000 (86 000 to 173 000)	748·2 (512·5 to 1037·6)
	Peru		1000 (1000 to 1000)	2·8 (1·6 to 4·2)	12 000 (4000 to 32 000)	36·8 (13·0 to 96·6)	230 000 (158 000 to 318 000)	692·9 (477·0 to 957·3)
Caribbean			3000 (2000 to 4000)	5·5 (3·6 to 7·7)	75 000 (28 000 to 188 000)	162·1 (61·6 to 406·5)	481 000 (350 000 to 631 000)	1039·3 (757·0 to 1364·8)
	Antigua and Barbuda		<1000 (<1000 to <1000)	6·3 (3·9 to 9·7)	<1000 (<1000 to <1000)	163·9 (56·8 to 436·1)	1000 (1000 to 1000)	1074·1 (742·4 to 1459·3)
	The Bahamas		<1000 (<1000 to <1000)	3·4 (2·0 to 5·3)	<1000 (<1000 to 1000)	101·1 (34·8 to 279·5)	3000 (2000 to 4000)	669·9 (460·0 to 941·9)
	Barbados		<1000 (<1000 to <1000)	10·8 (6·7 to 16·2)	1000 (<1000 to 2000)	233·8 (84·9 to 601·7)	4000 (3000 to 5000)	1331·8 (932·4 to 1789·8)
	Belize		<1000 (<1000 to <1000)	4·7 (2·8 to 7·3)	<1000 (<1000 to 1000)	124·4 (41·4 to 343·6)	4000 (3000 to 5000)	932·7 (636·7 to 1280·5)
	Bermuda		<1000 (<1000 to <1000)	4·5 (2·7 to 6·8)	<1000 (<1000 to <1000)	156·7 (56·3 to 405·9)	1000 (<1000 to 1000)	929·2 (649·9 to 1250·9)
	Cuba		1000 (<1000 to 1000)	6·1 (3·9 to 9·0)	16 000 (6000 to 42 000)	139·6 (51·1 to 365·7)	85 000 (60 000 to 115 000)	742·8 (523·4 to 1015·0)
	Dominica		<1000 (<1000 to <1000)	7·8 (4·7 to 11·9)	<1000 (<1000 to <1000)	188·9 (66·2 to 497·4)	1000 (1000 to 1000)	1148·9 (797·6 to 1555·4)
	Dominican Republic		<1000 (<1000 to 1000)	3·7 (2·0 to 5·9)	18 000 (6000 to 48 000)	167·9 (55·5 to 459·1)	121 000 (83 000 to 167 000)	1160·7 (791·9 to 1595·0)
	Grenada		<1000 (<1000 to <1000)	9·1 (5·7 to 13·7)	<1000 (<1000 to 1000)	184·5 (65·5 to 489·6)	1000 (1000 to 2000)	979·4 (683·6 to 1356·0)
	Guyana		<1000 (<1000 to <1000)	5·8 (3·4 to 9·0)	1000 (<1000 to 3000)	131·4 (44·1 to 356·6)	7000 (5000 to 9000)	920·3 (629·8 to 1260·3)
	Haiti		1000 (<1000 to 1000)	6·2 (3·2 to 11·0)	22 000 (7000 to 60 000)	182·9 (59·4 to 508·5)	146 000 (100 000 to 201 000)	1237·8 (844·5 to 1698·2)
	Jamaica		<1000 (<1000 to <1000)	3·2 (1·8 to 5·1)	4000 (1000 to 10 000)	128·0 (43·5 to 345·6)	25 000 (17 000 to 34 000)	893·9 (614·5 to 1 219·5)
	Puerto Rico		<1000 (<1000 to <1000)	7·8 (4·9 to 11·5)	8000 (3000 to 20 000)	207·4 (74·9 to 537·7)	43 000 (30 000 to 58 000)	1181·0 (826·6 to 1587·0)
	Saint Lucia		<1000 (<1000 to <1000)	5·7 (3·4 to 8·6)	<1000 (<1000 to 1000)	167·6 (58·4 to 445·6)	2000 (1000 to 3000)	1104·3 (764·7 to 1498·1)
	Saint Vincent and the Grenadines		<1000 (<1000 to <1000)	6·9 (4·2 to 10·5)	<1000 (<1000 to 1000)	177·0 (62·3 to 464·8)	1000 (1000 to 2000)	1112·0 (771·5 to 1506·6)
	Suriname		<1000 (<1000 to <1000)	5·4 (3·2 to 8·3)	1000 (<1000 to 2000)	130·3 (44·8 to 345·5)	5000 (3000 to 7000)	864·2 (597·0 to 1175·5)
	Trinidad and Tobago		<1000 (<1000 to <1000)	4·0 (2·2 to 6·5)	2000 (1000 to 5000)	141·8 (49·3 to 380·1)	13 000 (9000 to 18 000)	958·2 (663·1 to 1301·8)
	Virgin Islands		<1000 (<1000 to <1000)	5·4 (3·2 to 8·5)	<1000 (<1000 to <1000)	166·6 (59·7 to 428·7)	1000 (1000 to 1000)	995·9 (696·1 to 1342·3)
Central Latin America			3000 (2000 to 5000)	1·3 (0·9 to 1·8)	113 000 (41 000 to 295 000)	44·3 (16·0 to 115·5)	1 134 000 (776 000 to 1 573 000)	443·8 (303·9 to 615·8)
	Colombia		1000 (<1000 to 1000)	1·2 (0·7 to 1·8)	28 000 (9000 to 79 000)	55·0 (17·7 to 156·4)	273 000 (183 000 to 386 000)	539·1 (361·3 to 761·8)
	Costa Rica		<1000 (<1000 to <1000)	0·6 (0·4 to 1·0)	2000 (1000 to 5000)	38·5 (12·2 to 109·9)	19 000 (13 000 to 27 000)	412·6 (277·5 to 581·3)
	El Salvador		<1000 (<1000 to <1000)	1·3 (0·7 to 2·2)	2000 (1000 to 6000)	32·5 (10·6 to 91·4)	18 000 (12 000 to 26 000)	299·9 (201·3 to 419·3)
	Guatemala		1000 (<1000 to 1000)	3·2 (1·9 to 4·8)	13 000 (4000 to 36 000)	76·1 (24·7 to 214·7)	116 000 (77 000 to 166 000)	685·4 (455·3 to 983·1)
	Honduras		<1000 (<1000 to <1000)	0·7 (0·3 to 1·2)	6000 (2000 to 19 000)	66·2 (20·0 to 197·8)	61 000 (41 000 to 88 000)	645·9 (430·9 to 925·5)
	Mexico		2000 (1000 to 2000)	1·3 (0·9 to 1·9)	49 000 (18 000 to 126 000)	38·9 (14·6 to 99·8)	503 000 (343 000 to 704 000)	397·3 (271·0 to 555·9)
	Nicaragua		<1000 (<1000 to <1000)	0·4 (0·2 to 0·6)	1000 (<1000 to 4000)	20·2 (6·2 to 58·8)	13 000 (9000 to 19 000)	209·5 (139·7 to 296·0)
	Panama		<1000 (<1000 to <1000)	1·2 (0·7 to 1·8)	2000 (1000 to 5000)	49·2 (16·0 to 138·6)	18 000 (12 000 to 26 000)	463·8 (310·8 to 654·9)
	Venezuela		<1000 (<1000 to <1000)	0·9 (0·5 to 1·4)	11 000 (4000 to 31 000)	35·2 (11·5 to 99·4)	111 000 (75 000 to 156 000)	359·5 (241·7 to 506·9)
Tropical Latin America			6000 (4000 to 9000)	2·8 (1·8 to 3·9)	177 000 (73 000 to 414 000)	80·8 (33·2 to 189·4)	620 000 (420 000 to 871 000)	283·4 (192·1 to 398·2)
	Brazil		6000 (4000 to 8000)	2·8 (1·8 to 4·0)	164 000 (67 000 to 382 000)	77·3 (31·9 to 180·6)	569 000 (384 000 to 802 000)	268·6 (181·4 to 378·6)
	Paraguay		<1000 (<1000 to <1000)	2·7 (1·5 to 4·5)	14 000 (4000 to 39 000)	197·9 (64·3 to 557·8)	51 000 (35 000 to 72 000)	738·8 (498·6 to 1034·1)
**North Africa and Middle East**			**6000 (4000 to 8000)**	**0·9 (0·6 to 1·3)**	**746 000 (253 000 to 2 027 000)**	**124·2 (42·1 to 337·7)**	**4 656 000 (3 180 000 to 6 468 000)**	**775·7 (529·9 to 1077·7)**
Afghanistan			1000 (<1000 to 1000)	2·4 (1·2 to 4·1)	74 000 (22 000 to 223 000)	226·0 (67·3 to 679·5)	395 000 (260 000 to 571 000)	1 201·3 (791·6 to 1 737·8)
Algeria			<1000 (<1000 to 1000)	0·8 (0·4 to 1·3)	53 000 (17 000 to 152 000)	132·1 (42·9 to 374·9)	320 000 (217 000 to 452 000)	791·4 (535·2 to 1118·2)
Bahrain			<1000 (<1000 to <1000)	0·5 (0·3 to 0·8)	1000 (<1000 to 4000)	101·2 (32·1 to 294·5)	10 000 (7000 to 14 000)	692·0 (471·3 to 972·9)
Egypt			1000 (<1000 to 1000)	0·7 (0·4 to 1·3)	98 000 (30 000 to 281 000)	101·1 (30·8 to 291·1)	588 000 (395 000 to 818 000)	609·5 (409·2 to 848·3)
Iran			1000 (<1000 to 1000)	0·8 (0·5 to 1·2)	84 000 (29 000 to 230 000)	102·7 (35·1 to 280·3)	482 000 (332 000 to 675 000)	587·0 (404·4 to 820·9)
Iraq			<1000 (<1000 to <1000)	0·4 (0·2 to 0·7)	55 000 (16 000 to 165 000)	126·6 (37·9 to 382·1)	357 000 (238 000 to 512 000)	825·0 (549·4 to 1181·2)
Jordan			<1000 (<1000 to <1000)	0·6 (0·3 to 0·9)	12 000 (4000 to 32 000)	110·6 (38·3 to 302·8)	84 000 (57 000 to 120 000)	791·4 (530·7 to 1126·2)
Kuwait			<1000 (<1000 to <1000)	1·5 (0·9 to 2·2)	7000 (2000 to 19 000)	160·4 (52·1 to 453·3)	47 000 (32 000 to 65 000)	1093·8 (740·7 to 1529·4)
Lebanon			<1000 (<1000 to <1000)	0·6 (0·3 to 1·1)	12 000 (4000 to 38 000)	145·9 (44·8 to 442·3)	82 000 (55 000 to 116 000)	961·0 (644·5 to 1368·1)
Libya			<1000 (<1000 to <1000)	0·9 (0·5 to 1·5)	9000 (3000 to 27 000)	133·7 (42·7 to 391·6)	62 000 (42 000 to 88 000)	899·7 (608·8 to 1270·4)
Morocco			1000 (<1000 to 1000)	1·8 (1·0 to 3·1)	84 000 (27 000 to 237 000)	237·0 (76·7 to 669·0)	509 000 (345 000 to 711 000)	1435·4 (973·3 to 2002·5)
Oman			<1000 (<1000 to <1000)	0·6 (0·3 to 1·1)	5000 (2000 to 14 000)	105·6 (33·7 to 307·5)	35 000 (24 000 to 49 000)	775·2 (528·3 to 1089·3)
Palestine			<1000 (<1000 to <1000)	0·7 (0·4 to 1·2)	7000 (2000 to 20 000)	139·3 (42·9 to 413·0)	44 000 (29 000 to 62 000)	898·2 (599·7 to 1283·4)
Qatar			<1000 (<1000 to <1000)	0·2 (0·1 to 0·4)	3000 (1000 to 8000)	102·7 (32·7 to 298·7)	19 000 (13 000 to 26 000)	687·1 (470·4 to 961·9)
Saudi Arabia			<1000 (<1000 to <1000)	0·8 (0·4 to 1·3)	36 000 (12 000 to 104 000)	103·9 (33·6 to 303·0)	267 000 (181 000 to 374 000)	773·8 (526·7 to 1086·8)
Sudan			<1000 (<1000 to 1000)	1·1 (0·5 to 2·1)	64 000 (19 000 to 191 000)	157·8 (47·2 to 475·7)	354 000 (235 000 to 510 000)	880·4 (584·4 to 1265·8)
Syria			<1000 (<1000 to <1000)	0·8 (0·4 to 1·3)	22 000 (7000 to 64 000)	119·4 (37·3 to 350·5)	148 000 (100 000 to 210 000)	817·0 (548·9 to 1160·6)
Tunisia			<1000 (<1000 to <1000)	1·0 (0·6 to 1·8)	18 000 (6000 to 51 000)	155·6 (50·9 to 442·1)	103 000 (70 000 to 146 000)	903·0 (612·5 to 1272·9)
Turkey			1000 (<1000 to 1000)	0·7 (0·4 to 1·1)	44 000 (17 000 to 110 000)	54·7 (20·7 to 136·6)	342 000 (239 000 to 466 000)	425·2 (296·9 to 578·6)
United Arab Emirates			<1000 (<1000 to <1000)	0·5 (0·2 to 0·9)	7000 (2000 to 22 000)	76·6 (25·1 to 221·6)	58 000 (40 000 to 81 000)	595·2 (410·6 to 829·8)
Yemen			<1000 (<1000 to 1000)	1·0 (0·5 to 1·8)	61 000 (18 000 to 186 000)	200·8 (60·8 to 609·6)	347 000 (230 000 to 499 000)	1139·5 (756·7 to 1637·9)
**South Asia**			**31 000 (20 000 to 45 000)**	**1·8 (1·1 to 2·6)**	**765 000 (255 000 to 2 096 000)**	**42·9 (14·3 to 117·6)**	**18 950 000 (12 935 000 to 26 374 000)**	**1063·0 (725·6 to 1479·4)**
Bangladesh			1000 (1000 to 2000)	0·6 (0·3 to 1·1)	19 000 (6000 to 53 000)	11·9 (3·7 to 33·8)	719 000 (484 000 to 1 006 000)	457·7 (308·3 to 640·6)
Bhutan			<1000 (<1000 to <1000)	1·0 (0·5 to 1·7)	<1000 (<1000 to 1000)	20·8 (6·2 to 61·4)	9000 (6000 to 12 000)	912·2 (616·3 to 1274·4)
India			26 000 (16 000 to 37 000)	1·8 (1·2 to 2·7)	588 000 (196 000 to 1 611 000)	42·6 (14·2 to 116·7)	13 966 000 (9 449 000 to 19 552 000)	1011·6 (684·4 to 1416·3)
Nepal			<1000 (<1000 to 1000)	1·6 (0·8 to 2·7)	3000 (1000 to 8000)	9·4 (3·2 to 25·7)	208 000 (140 000 to 293 000)	695·5 (468·8 to 979·4)
Pakistan			4000 (2000 to 8000)	2·0 (1·0 to 3·7)	126 000 (38 000 to 378 000)	58·8 (17·6 to 176·5)	4 059 000 (2 721 000 to 5 746 000)	1894·2 (1269·6 to 2681·5)
**Southeast Asia, east Asia, and Oceania**			**27 000 (18 000 to 37 000)**	**1·2 (0·9 to 1·7)**	**2 330 000 (919 000 to 5 592 000)**	**107·9 (42·6 to 259·0)**	**13 256 000 (9 342 000 to 17 963 000)**	**614·0 (432·8 to 832·1)**
East Asia			14 000 (10 000 to 20 000)	1·0 (0·7 to 1·4)	884 000 (336 000 to 2 168 000)	59·5 (22·6 to 145·9)	2 571 000 (1 787 000 to 3 510 000)	173·1 (120·3 to 236·3)
	China		11 000 (7000 to 16 000)	0·8 (0·5 to 1·1)	739 000 (279 000 to 1 826 000)	52·4 (19·8 to 129·2)	2 145 000 (1 481 000 to 2 941 000)	151·8 (104·9 to 208·2)
	North Korea		1000 (<1000 to 1000)	2·3 (1·2 to 4·1)	50 000 (16 000 to 140 000)	196·2 (63·6 to 545·1)	156 000 (106 000 to 217 000)	607·3 (413·2 to 845·0)
	Taiwan (Province of China)		3000 (2000 to 4000)	12·1 (7·8 to 17·6)	80 000 (28 000 to 206 000)	338·8 (119·7 to 874·4)	230 000 (161 000 to 310 000)	976·6 (681·4 to 1315·9)
Oceania			<1000 (<1000 to 1000)	3·9 (2·2 to 6·6)	19 000 (6000 to 54 000)	152·3 (49·9 to 431·3)	192 000 (134 000 to 264 000)	1527·2 (1066·2 to 2097·6)
	American Samoa		<1000 (<1000 to <1000)	1·7 (1·0 to 2·9)	<1000 (<1000 to <1000)	107·5 (34·8 to 307·9)	1000 (<1000 to 1000)	1110·5 (762·2 to 1545·2)
	Federated States of Micronesia		<1000 (<1000 to <1000)	2·2 (1·0 to 3·9)	<1000 (<1000 to <1000)	116·7 (37·6 to 333·0)	1000 (1000 to 2000)	1207·7 (830·6 to 1676·9)
	Fiji		<1000 (<1000 to <1000)	2·3 (1·3 to 3·8)	1000 (<1000 to 3000)	113·9 (37·3 to 319·8)	10 000 (7000 to 14 000)	1121·0 (773·0 to 1554·0)
	Guam		<1000 (<1000 to <1000)	2·0 (1·2 to 3·3)	<1000 (<1000 to <1000)	106·8 (35·7 to 297·0)	2000 (1000 to 2000)	1043·7 (721·0 to 1443·4)
	Kiribati		<1000 (<1000 to <1000)	1·8 (0·9 to 3·2)	<1000 (<1000 to <1000)	102·4 (31·0 to 316·5)	1000 (1000 to 2000)	989·9 (656·3 to 1433·4)
	Marshall Islands		<1000 (<1000 to <1000)	2·6 (1·3 to 4·6)	<1000 (<1000 to <1000)	118·0 (38·1 to 335·7)	1000 (<1000 to 1000)	1249·0 (860·2 to 1733·6)
	Northern Mariana Islands		<1000 (<1000 to <1000)	1·7 (1·0 to 2·8)	<1000 (<1000 to <1000)	101·1 (34·4 to 278·1)	<1000 (<1000 to 1000)	998·2 (690·9 to 1376·9)
	Papua New Guinea		<1000 (<1000 to 1000)	4·3 (2·2 to 7·6)	15 000 (5000 to 44 000)	162·7 (51·5 to 475·2)	151 000 (103 000 to 210 000)	1635·6 (1120·3 to 2279·0)
	Samoa		<1000 (<1000 to <1000)	1·7 (0·9 to 3·0)	<1000 (<1000 to 1000)	120·1 (37·9 to 350·0)	2000 (2000 to 3000)	1145·5 (784·3 to 1597·3)
	Solomon Islands		<1000 (<1000 to <1000)	3·9 (2·1 to 6·6)	1000 (<1000 to 3000)	132·0 (40·3 to 400·8)	8000 (6000 to 12 000)	1309·6 (869·6 to 1894·7)
	Tonga		<1000 (<1000 to <1000)	1·7 (0·9 to 2·9)	<1000 (<1000 to <1000)	94·2 (29·5 to 285·1)	1000 (1000 to 1000)	921·7 (612·1 to 1331·6)
	Vanuatu		<1000 (<1000 to <1000)	2·6 (1·3 to 4·9)	<1000 (<1000 to 1000)	122·6 (38·2 to 356·5)	3000 (2000 to 5000)	1146·5 (784·5 to 1600·2)
Southeast Asia			12 000 (8000 to 17 000)	1·8 (1·2 to 2·5)	205 000 (77 000 to 518 000)	31·0 (11·6 to 78·4)	10 509 000 (7 385 000 to 14 268 000)	1591·2 (1118·1 to 2160·2)
	Cambodia		<1000 (<1000 to <1000)	1·4 (0·7 to 2·3)	2000 (1 000 to 7000)	14·7 (4·6 to 42·7)	132 000 (88 000 to 188 000)	818·8 (545·2 to 1166·6)
	Indonesia		3000 (2000 to 4000)	1·2 (0·7 to 1·7)	40 000 (14 000 to 108 000)	15·7 (5·6 to 41·8)	3 317 000 (2 261 000 to 4 619 000)	1285·0 (876·0 to 1789·3)
	Laos		<1000 (<1000 to 1000)	4·2 (2·1 to 7·4)	3000 (1000 to 8000)	37·9 (11·9 to 107·8)	153 000 (105 000 to 210 000)	2190·0 (1504·6 to 3016·5)
	Malaysia		1000 (<1000 to 1000)	2·6 (1·3 to 4·1)	6000 (2000 to 17 000)	20·2 (6·6 to 56·9)	342 000 (230 000 to 480 000)	1116·8 (752·2 to 1567·2)
	Maldives		<1000 (<1000 to <1000)	0·3 (0·2 to 0·5)	<1000 (<1000 to <1000)	18·4 (5·7 to 53·2)	7000 (5000 to 9000)	1462·2 (1008·8 to 2018·4)
	Mauritius		<1000 (<1000 to <1000)	1·3 (0·8 to 1·9)	<1000 (<1000 to 1000)	21·0 (7·2 to 56·1)	14 000 (10 000 to 20 000)	1123·4 (776·2 to 1547·9)
	Myanmar		1000 (1000 to 2000)	2·1 (1·1 to 3·5)	11 000 (4000 to 30 000)	20·8 (6·8 to 57·6)	609 000 (418 000 to 845 000)	1153·9 (792·6 to 1599·8)
	Philippines		2000 (1000 to 3000)	2·0 (1·2 to 3·0)	22 000 (8000 to 58 000)	20·9 (7·3 to 55·9)	1 169 000 (798 000 to 1 616 000)	1129·6 (771·3 to 1561·5)
	Sri Lanka		<1000 (<1000 to <1000)	1·2 (0·7 to 2·2)	6000 (2000 to 15 000)	25·6 (8·5 to 70·6)	297 000 (205 000 to 411 000)	1376·9 (949·2 to 1904·4)
	Seychelles		<1000 (<1000 to <1000)	3·7 (2·2 to 5·7)	<1000 (<1000 to <1000)	32·2 (11·0 to 86·6)	2000 (1000 to 2000)	1591·2 (1099·7 to 2191·8)
	Thailand		2000 (1000 to 3000)	3·0 (1·5 to 4·7)	20 000 (7000 to 53 000)	28·4 (10·1 to 74·9)	869 000 (608 000 to 1 189 000)	1231·1 (861·4 to 1682·8)
	Timor-Leste		<1000 (<1000 to <1000)	1·7 (0·7 to 3·0)	<1000 (<1000 to 1000)	27·4 (8·5 to 79·4)	19 000 (13 000 to 27 000)	1469·8 (999·5 to 2060·4)
	Vietnam		2000 (1000 to 3000)	2·1 (1·2 to 3·6)	135 000 (48 000 to 356 000)	139·9 (49·7 to 370·3)	3 567 000 (2 439 000 to 4 943 000)	3710·5 (2537·3 to 5141·6)
**Sub-Saharan Africa**			**27 000 (17 000 to 40 000)**	**2·7 (1·7 to 3·9)**	**1 598 000 (556 000 to 4 250 000)**	**155·7 (54·2 to 414·2)**	**6 052 000 (4 189 000 to 8 318 000)**	**589·8 (408·2 to 810·6)**
Central sub-Saharan Africa			4000 (2000 to 7000)	3·4 (1·9 to 5·5)	293 000 (98 000 to 812 000)	241·0 (80·6 to 667·2)	937 000 (636 000 to 1 310 000)	770·0 (522·7 to 1076·9)
	Angola		1000 (<1000 to 1000)	2·5 (1·3 to 4·3)	52 000 (17 000 to 145 000)	184·4 (61·2 to 512·5)	170 000 (116 000 to 238 000)	603·8 (410·1 to 845·2)
	Central African Republic		<1000 (<1000 to <1000)	5·2 (2·5 to 9·3)	12 000 (4000 to 35 000)	265·4 (86·5 to 746·9)	39 000 (27 000 to 55 000)	849·0 (577·7 to 1185·4)
	Congo (Brazzaville)		<1000 (<1000 to <1000)	2·8 (1·5 to 4·8)	10 000 (3000 to 27 000)	199·1 (66·4 to 550·2)	32 000 (22 000 to 45 000)	660·6 (452·3 to 918·9)
	Democratic Republic of the Congo		3000 (2000 to 5000)	3·6 (1·9 to 6·4)	214 000 (69 000 to 608 000)	264·0 (85·4 to 751·2)	675 000 (455 000 to 954 000)	834·4 (562·1 to 1179·2)
	Equatorial Guinea		<1000 (<1000 to <1000)	1·5 (0·8 to 2·8)	2000 (1000 to 7000)	173·1 (55·7 to 494·4)	8000 (6000 to 12 000)	631·2 (429·3 to 879·6)
	Gabon		<1000 (<1000 to <1000)	2·6 (1·4 to 4·4)	3000 (1000 to 10 000)	204·6 (68·9 to 562·4)	12 000 (8000 to 16 000)	677·5 (465·3 to 939·2)
Eastern sub-Saharan Africa			7000 (4000 to 10 000)	1·7 (1·1 to 2·5)	430 000 (146 000 to 1 167 000)	109·4 (37·1 to 296·9)	2 183 000 (1 504 000 to 2 994 000)	555·1 (382·4 to 761·4)
	Burundi		<1000 (<1000 to <1000)	1·9 (1·0 to 3·4)	13 000 (4000 to 37 000)	117·3 (37·0 to 335·0)	66 000 (45 000 to 93 000)	609·1 (410·8 to 850·4)
	Comoros		<1000 (<1000 to <1000)	1·8 (1·0 to 3·0)	1000 (<1000 to 2000)	102·8 (34·2 to 282·6)	4000 (3000 to 5000)	545·3 (372·0 to 753·7)
	Djibouti		<1000 (<1000 to <1000)	1·3 (0·7 to 2·4)	1000 (<1000 to 3000)	89·5 (29·2 to 251·6)	5000 (4000 to 7000)	477·9 (325·3 to 662·1)
	Eritrea		<1000 (<1000 to <1000)	2·0 (1·0 to 3·4)	7000 (2000 to 19 000)	112·5 (36·0 to 321·1)	36 000 (25 000 to 51 000)	620·2 (419·3 to 863·7)
	Ethiopia		1000 (1000 to 2000)	1·0 (0·6 to 1·5)	69 000 (23 000 to 192 000)	67·4 (22·1 to 186·2)	339 000 (230 000 to 468 000)	329·3 (223·7 to 454·6)
	Kenya		1000 (1000 to 1000)	1·8 (1·1 to 2·7)	74 000 (24 000 to 206 000)	152·3 (50·1 to 427·0)	366 000 (245 000 to 512 000)	756·5 (506·7 to 1 060·2)
	Madagascar		1000 (<1000 to 2000)	3·4 (1·7 to 5·9)	57 000 (19 000 to 164 000)	219·6 (71·1 to 629·7)	300 000 (204 000 to 417 000)	1 147·5 (780·7 to 1597·5)
	Malawi		<1000 (<1000 to <1000)	2·0 (1·1 to 3·4)	22 000 (7000 to 61 000)	125·6 (39·9 to 357·4)	110 000 (74 000 to 154 000)	640·2 (429·1 to 897·7)
	Mozambique		<1000 (<1000 to <1000)	0·9 (0·5 to 1·5)	16 000 (5000 to 47 000)	53·3 (17·0 to 155·2)	89 000 (60 000 to 126 000)	297·0 (201·1 to 419·0)
	Rwanda		<1000 (<1000 to <1000)	1·5 (0·8 to 2·5)	13 000 (4000 to 39 000)	105·8 (32·8 to 309·4)	68 000 (44 000 to 97 000)	538·5 (352·0 to 774·3)
	Somalia		<1000 (<1000 to 1000)	2·1 (1·0 to 3·8)	17 000 (5000 to 50 000)	102·3 (32·1 to 297·8)	88 000 (60 000 to 124 000)	523·5 (352·6 to 731·9)
	South Sudan		<1000 (<1000 to 1000)	3·0 (1·5 to 5·3)	12 000 (4000 to 35 000)	122·2 (37·9 to 354·0)	60 000 (40 000 to 84 000)	602·3 (404·3 to 844·3)
	Tanzania		1000 (1000 to 2000)	2·7 (1·4 to 4·4)	75 000 (25 000 to 213 000)	139·9 (45·6 to 394·4)	385 000 (261 000 to 534 000)	712·6 (484·4 to 989·0)
	Uganda		<1000 (<1000 to 1000)	1·2 (0·6 to 2·0)	40 000 (13 000 to 116 000)	102·6 (32·3 to 296·7)	212 000 (143 000 to 298 000)	542·0 (365·7 to 762·4)
	Zambia		<1000 (<1000 to <1000)	1·1 (0·6 to 1·8)	10 000 (3000 to 28 000)	58·0 (18·8 to 163·1)	54 000 (37 000 to 75 000)	308·7 (210·7 to 430·7)
Southern sub-Saharan Africa			2000 (1000 to 3000)	3·0 (1·9 to 4·4)	156 000 (57 000 to 399 000)	201·4 (73·3 to 515·2)	532 000 (373 000 to 727 000)	687·9 (482·0 to 939·2)
	Botswana		<1000 (<1000 to <1000)	1·8 (1·0 to 3·0)	3000 (1000 to 9000)	145·6 (49·3 to 391·3)	12 000 (9000 to 17 000)	547·1 (376·2 to 751·6)
	eSwatini		<1000 (<1000 to <1000)	3·2 (1·6 to 5·6)	3000 (1000 to 7000)	237·6 (78·8 to 661·6)	10 000 (7000 to 14 000)	881·0 (603·6 to 1226·0)
	Lesotho		<1000 (<1000 to <1000)	4·4 (2·2 to 7·6)	5000 (2000 to 14 000)	270·1 (91·0 to 739·7)	19 000 (13 000 to 26 000)	968·2 (666·4 to 1341·9)
	Namibia		<1000 (<1000 to <1000)	2·6 (1·4 to 4·4)	5000 (2000 to 14 000)	221·2 (74·1 to 608·2)	19 000 (14 000 to 26 000)	787·5 (540·0 to 1094·2)
	South Africa		2000 (1000 to 2000)	2·8 (1·8 to 4·0)	108 000 (40 000 to 275 000)	196·5 (71·9 to 499·7)	365 000 (256 000 to 497 000)	663·4 (465·5 to 905·1)
	Zimbabwe		1000 (<1000 to 1000)	3·9 (2·0 to 6·6)	31 000 (11 000 to 86 000)	212·6 (71·4 to 582·5)	108 000 (74 000 to 151 000)	733·6 (500·4 to 1024·4)
Western sub-Saharan Africa			14 000 (9000 to 22 000)	3·3 (2·0 to 5·1)	736 000 (251 000 to 1 996 000)	169·7 (57·8 to 460·0)	2 408 000 (1 644 000 to 3 354 000)	555·2 (379·0 to 773·2)
	Benin		<1000 (<1000 to 1000)	3·2 (1·6 to 5·6)	20 000 (7000 to 55 000)	171·4 (56·5 to 475·8)	66 000 (45 000 to 92 000)	572·0 (388·6 to 797·7)
	Burkina Faso		1000 (<1000 to 2000)	4·8 (2·4 to 8·8)	48 000 (15 000 to 146 000)	225·6 (69·2 to 693·2)	151 000 (96 000 to 227 000)	713·1 (452·8 to 1075·7)
	Cameroon		1000 (<1000 to 1000)	3·1 (1·6 to 5·3)	51 000 (17 000 to 140 000)	182·5 (59·8 to 507·5)	171 000 (116 000 to 238 000)	616·1 (419·0 to 858·8)
	Cape Verde		<1000 (<1000 to <1000)	2·6 (1·5 to 4·2)	1000 (<1000 to 3000)	172·9 (60·0 to 459·4)	3000 (2000 to 4000)	597·8 (412·9 to 821·4)
	Chad		1000 (<1000 to 2000)	5·9 (3·0 to 10·0)	42 000 (13 000 to 119 000)	273·9 (86·4 to 780·6)	126 000 (85 000 to 177 000)	827·9 (556·4 to 1165·0)
	Côte d'Ivoire		2000 (1000 to 3000)	6·8 (3·6 to 11·9)	99 000 (33 000 to 273 000)	396·4 (131·8 to 1094·4)	327 000 (223 000 to 455 000)	1308·3 (891·4 to 1824·0)
	The Gambia		<1000 (<1000 to <1000)	2·4 (1·3 to 4·0)	3000 (1000 to 9000)	144·9 (47·2 to 411·7)	10 000 (7000 to 15 000)	482·4 (320·5 to 689·2)
	Ghana		1000 (<1000 to 1000)	2·6 (1·4 to 4·4)	45 000 (15 000 to 124 000)	147·6 (49·1 to 411·5)	155 000 (104 000 to 218 000)	511·9 (345·1 to 721·8)
	Guinea		1000 (<1000 to 1000)	5·0 (2·7 to 8·4)	30 000 (10 000 to 84 000)	254·6 (83·6 to 709·6)	94 000 (64 000 to 131 000)	796·1 (539·8 to 1111·9)
	Guinea-Bissau		<1000 (<1000 to <1000)	3·3 (1·7 to 5·7)	4000 (1000 to 10 000)	190·5 (62·0 to 532·3)	12 000 (8000 to 17 000)	637·3 (432·4 to 889·9)
	Liberia		<1000 (<1000 to <1000)	2·5 (1·3 to 4·4)	10 000 (3000 to 29 000)	215·8 (70·3 to 603·7)	33 000 (23 000 to 47 000)	706·8 (480·1 to 986·0)
	Mali		1000 (<1000 to 1000)	2·6 (1·3 to 4·6)	30 000 (10 000 to 86 000)	148·3 (47·1 to 423·9)	97 000 (65 000 to 136 000)	476·6 (322·8 to 669·9)
	Mauritania		<1000 (<1000 to <1000)	2·5 (1·3 to 4·3)	7000 (2000 to 20 000)	179·7 (58·8 to 505·1)	23 000 (16 000 to 33 000)	597·5 (406·6 to 831·9)
	Niger		1000 (<1000 to 2000)	4·3 (2·2 to 7·7)	50 000 (16 000 to 142 000)	234·4 (75·4 to 665·9)	155 000 (104 000 to 218 000)	724·9 (488·2 to 1018·3)
	Nigeria		5000 (3000 to 9000)	2·6 (1·4 to 4·6)	231 000 (75 000 to 655 000)	112·3 (36·2 to 317·7)	758 000 (512 000 to 1 063 000)	368·0 (248·3 to 515·8)
	São Tomé and Príncipe		<1000 (<1000 to <1000)	3·4 (1·9 to 5·6)	<1000 (<1000 to 1000)	229·1 (77·1 to 631·8)	2000 (1000 to 2000)	811·2 (555·1 to 1124·5)
	Senegal		<1000 (<1000 to 1000)	2·7 (1·4 to 4·6)	34 000 (11 000 to 93 000)	228·6 (75·3 to 632·5)	106 000 (72 000 to 148 000)	720·3 (488·4 to 1006·3)
	Sierra Leone		<1000 (<1000 to 1000)	4·4 (2·3 to 7·7)	20 000 (6000 to 54 000)	250·6 (82·2 to 692·7)	64 000 (44 000 to 90 000)	821·1 (557·3 to 1146·1)
	Togo		<1000 (<1000 to <1000)	2·6 (1·4 to 4·5)	14 000 (5000 to 39 000)	190·2 (63·1 to 524·7)	48 000 (33 000 to 67 000)	638·0 (435·4 to 886·4)

Numbers are rounded to the nearest thousand. If the value was less than 1000, it is shown as <1000. Exact estimates are available online. GBD=Global Burden of Disease Study. UI=uncertainty interval.

**Figure 4:**
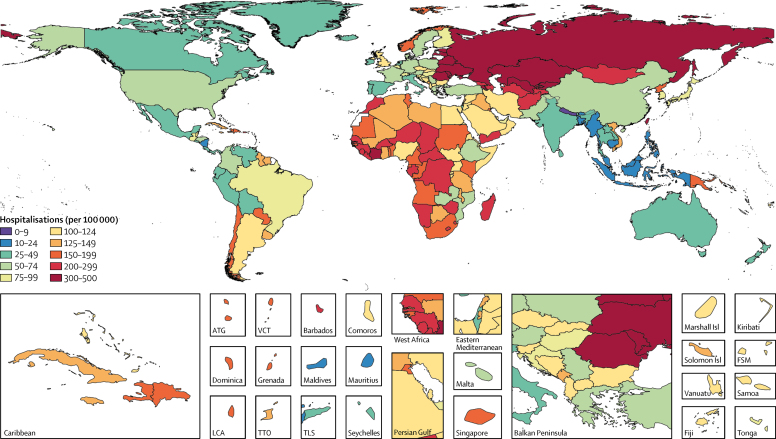
Influenza lower respiratory tract infection hospitalisations per 100 000 for all ages, 2017 ATG=Antigua and Barbuda. FSM=Federated States of Micronesia. LCA=Saint Lucia. VCT=Saint Vincent and the Grenadines. TLS=Timor-Leste. TTO=Trinidad and Tobago. Isl=Islands.

Influenza was more frequently associated with non-fatal or non-severe LRTI episodes than with fatal or severe episodes. We estimated that influenza was present 23% (95% UI 9–33) less frequently in hospitalised LRTI cases than in non-hospitalised LRTI episodes. This finding was also shown by the proportionally greater attribution of influenza to non-fatal LRTI than to fatal LRTI (figure 2). Among all ages, an estimated 8·5% (95% UI 5·4–13·5) of LRTI hospitalisations were attributable to influenza (figure 2). We estimated that influenza was responsible for 9 459 000 (95% UI 3 709 000–22 935 000) LRTI hospitalisations —a rate of 123·8 per 100 000 (95% UI 48·5–300·2; table) in 2017—corresponding to an estimated 81 536 000 (95% UI 24 330 000–259 851 000) hospital days due to influenza LRTI. The greatest number of influenza LRTI episodes and hospitalisations occurred among children younger than 10 years (figure 2). We estimated that there were 2 224 000 (95% UI 738 000–5 979 000) LRTI hospitalisations due to influenza among children younger than 5 years in 2017. The incidences of non-hospitalised and hospitalised influenza LRTIs were high in children and elderly people, resulting in a U-shaped curve when graphed (figure 2). The incidence per 100 000 population of hospitalisation due to influenza LRTI was greatest in eastern Europe (488·7 [95% UI 185·9–1204·6]) and central Asia (303·1 [120·5–721·6]; figure 4; table). The countries with the highest estimated rates of influenza LRTI hospitalisation per 100 000 population were Lithuania (560·7 [227·2–1351·7]) and Russia (494·4 [183·6–1241·6]), whereas Nepal (9·4 [3·2–25·7]) and Bangladesh (11·9 [3·7–33·8]) had the lowest rates per 100 000 (figure 4; table). Globally in 2017, 17·4% (95% UI 9·6–31·0) of influenza LRTI cases were hospitalised among all ages (appendix pp 28–29). The proportion hospitalised was highest in adults older than 70 years (appendix p 29). Indonesia (1·2% [0·6–2·3]) and the Maldives (1·3% [0·6–2·6]) had the lowest estimated proportions of influenza LRTI episodes that were hospitalised, whereas Singapore (59·0% [31·8–100·0]) and Brunei (59·1% [29·6–100·0]) had the highest proportions (appendix p 28). Although the number of influenza LRTI hospitalisations increased by 14·0% between 1990 and 2017 (from 8 300 000 to 9 459 000), the hospitalisation rate declined over the same period by 19·6% (from 153·9 per 100 000 to 123·8 per 100 000; data not shown).

**Figure 5:**
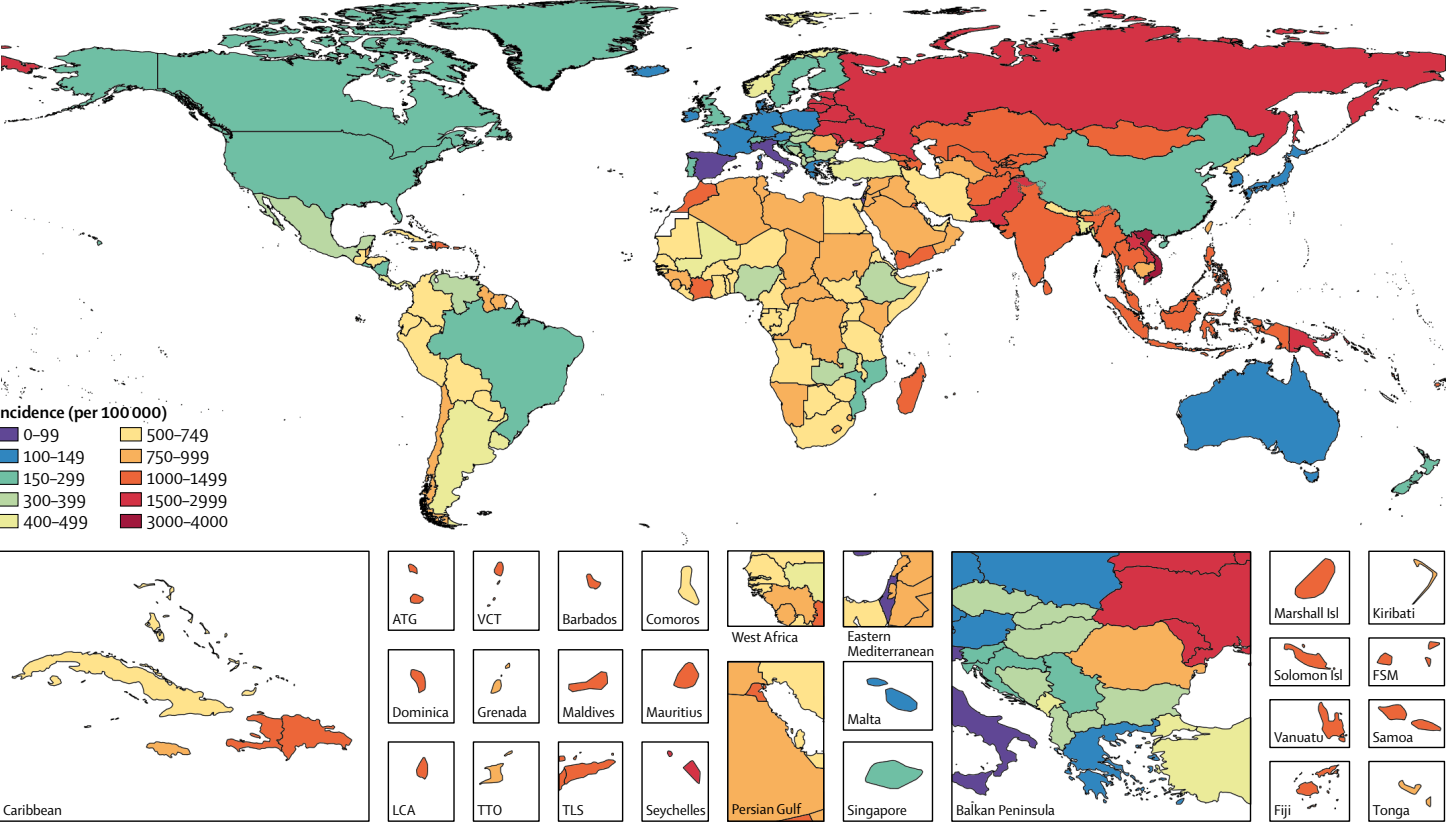
Influenza lower respiratory tract infection incidence per 100 000 for all ages, 2017 ATG=Antigua and Barbuda. FSM=Federated States of Micronesia. LCA=Saint Lucia. VCT=Saint Vincent and the Grenadines. TLS=Timor-Leste. TTO=Trinidad and Tobago. Isl=Islands.

Among all ages, we estimated that 11·5% (95% UI 10·0–12·9) of LRTI episodes were attributable to influenza (figure 2). This attributable fraction was highest among people aged 30–54 years (figure 2). 9·2% (95% UI 8·0–10·2) of LRTI episodes among children younger than 5 years and 10·8% (9·3–12·0%) among adults older than 70 years were attributable to influenza (figure 2). We estimated that influenza was responsible for 54 481 000 (95% UI 38 465 000–73 864 000) LRTI episodes among all ages in 2017 (table), including 8 172 000 (5 000 000–13 296 000) severe LRTI episodes. Influenza episodes were most common in eastern Europe (2399·3 episodes per 100 000 [95% UI 1717·2–3205·6]) and southeast Asia (1591·2 per 100 000 [1118·1–2160·2]; figure 5; table). The highest overall incidences per 100 000 population were in Vietnam (3710·5 [95% UI 2537·3–5141·6]) and Lithuania (2489·6 [1728·2–3469·3]), whereas the lowest incidences were in Italy (63·4 [44·5–85·1]) and Israel (83·2 [57·3–117·2]; table). The incidence of influenza LRTIs decreased by 9·7% between 1990 and 2017 (from 789·9 per 100 000 to 713·1 per 100 000), but increased in young adults aged 15–49 years by 12·1% (from 566·1 per 100 000 to 634·6 per 100 000; data not shown).

## Discussion

In this Article, we provide the most thorough and comprehensive analysis of the burden of LRTIs attributable to influenza so far. We provide estimates of the incidence, incidence of severe disease, hospitalisations, and mortality for each age group and by geographical region for 2017. We estimated that there were 54 481 000 (95% UI 38 465 000–73 864 000) LRTI episodes attributable to influenza, 8 172 000 (5 000 000–13 296 000) of which were severe, that caused 145 000 (99 000–200 000) deaths and 9 459 000 (3 709 000–22 935 000) hospitalisations.

A full picture of LRTIs and upper respiratory infections (including the non-respiratory infections), including symptomatic and inapparent infections, hospitalisations, and deaths, is necessary to quantify the entire spectrum of influenza illness (figure 1). This study has captured many, but not all those components. Inclusion of inapparent or asymptomatic influenza virus infection is crucial to model the transmission of influenza spread. The GBD does not typically estimate asymptomatic infections for causes of disease. There is evidence that influenza virus infection could be a risk factor for cardiovascular mortality,^17^ renal failure, and other systemic outcomes, perhaps through exacerbation of underlying or comorbid diseases. These systemic outcomes could begin with a respiratory infection and might be captured in time-series analyses of influenza burden or through influenza vaccine probe studies with severe illness endpoints to measure vaccine-preventable disease incidence.^18^ Our study quantified the remaining aspects of influenza disease burden, by focusing exclusively on LRTIs attributable to influenza. We have shown that a moderate amount of LRTIs are attributable to influenza. The attributable fraction is highest in adolescents and adults, but because of the greater overall risk of LRTIs in young children and elderly adults, our results suggest that the greatest number of LRTI episodes, deaths, and hospitalisations occur in young children and elderly adults.

Age patterns in rates and counts should be considered when developing appropriate responses and interventions. Global estimates of coverage of the seasonal influenza vaccine, which could provide additional evidence of disease burden, were not available for this study. Although we did not account for influenza vaccine coverage, our results help to point to where a seasonal or universal influenza vaccine would have the greatest effect. Modelling studies have suggested that vaccination of targeted populations could reduce seasonal influenza outbreaks,^19^ and could help to prevent episodes and hospitalisations in elderly adults.^20^ Our results suggest that the potential effect of vaccination on the burden of influenza LRTIs—ie, reductions in severe episodes, non-severe episodes, and deaths—might vary, and that targeting of specific age groups, particularly elderly people, and regions such as eastern Europe and southeast Asia could substantially reduce the global burden of influenza LRTIs. The counterfactual strategy used in our model allows for quantification of the avertable burden through vaccination or other interventions.

To estimate the attributable fraction of LRTI that was due to influenza, we made several important analytic decisions and assumptions. Our analysis of available data that reflected the frequency of influenza isolation among respiratory samples from pneumonia or bronchiolitis episodes (ie, the definition of LRTI in GBD 2017) showed that RT-PCR is significantly more sensitive than ELISA-based methods for detection of acute influenza infection. The mean frequency of influenza-positive samples was about 25% greater with RT-PCR than with non-RT-PCR diagnostics (appendix p 5), a result that is consistent with the general body of knowledge about molecular diagnostic methods.^21^ Another systematic review^22^ of influenza detection among acute LRTI episodes in children showed that PCR diagnostics detected influenza more than twice as frequently as immunofluorescence tests. One of the explanations for this finding could be the anatomical site where samples were collected, which can affect the frequency of influenza detection. Nasopharyngeal swabs were used in more studies because samples are easy to collect and the process is non-invasive.^23^ The data included in our systematic review were nearly exclusively based on nasopharyngeal or oropharyngeal swabs. Studies have shown varying levels of specificity and sensitivity for the relationship between detection based on nasopharyngeal samples and LRTIs.^23^, ^24^ The time between infection and onset of symptoms, geographical regions, and sample collection could also have crucial roles in influenza virus detection with or without molecular diagnostics.

Our aetiological attribution for influenza is based on approximately 650 data points from 100 sources in 40 countries (appendix pp 6–24). The predictive modelling tools used in the GBD are based on space-time information and covariates to help to make estimates for every geographical region, year, and age. In areas with sparse data, uncertainty around the estimates is greater than that in areas with many data. The modelled frequency of influenza attributable LRTI episodes is a component of the counterfactual attribution strategy used in GBD 2017. The second component of the PAF model, the OR, reflects how likely a sample that tests positive for influenza is to identify the aetiology of LRTI. The source that we used to quantify this relationship was a systematic review^12^ of studies of children younger than 5 years. Because of the absence of data for this relationship in older age groups, we assumed that the relationship was constant across ages—an assumption that might not hold because of immunological or biological differences between young children and adults. If adults are more likely than children younger than 5 years to have an LRTI episode if influenza is detected, our estimates in adults might be an underestimate. Conversely if adults are less likely to have an LRTI episode if influenza is detected than are children under 5, our results might be an overestimate. A study^25^ in South Africa showed that the odds of severe acute respiratory illness if influenza was present was lower among 5–64-year-olds than among people younger than 5 years or older than 65 years. Although those results come from only one study with a different case definition from what we used, they suggest that we might have overestimated the influenza attribution in people aged 5–64 years.

There is evidence of a potentially important role in influenza for co-infection with *S pneumoniae, Staphylococcus aureus, H influenzae* type b, and other bacterial pneumonias.^26^, ^27^, ^28^ The interpretation of the counterfactual attribution used in the GBD, although estimated independently for each pathogen, allows for overlap in the attribution of LRTI episodes or deaths due to multiple pathogens. However, our estimation does not explicitly account for influenza infection as a risk factor for subsequent bacterial infections, which is a potentially important burden.

The GBD 2017 estimates of LRTI deaths attributable to influenza are about two times greater than the estimates from GBD 2016 (appendix pp 31–33). This change is largely because of an important adjustment scalar in calculating how frequently viral causes of LRTI are associated with mortality compared with bacterial causes of LRTI. Our analysis showed that bacterial aetiologies are more likely to lead to death than viral aetiologies.^4^ For previous GBD studies, this relationship was quantified on the basis of data from a small subset of countries. We reproduced this analysis for GBD 2017 with a much larger dataset of hospitalisation records that were coded specifically to viral or bacterial causes of LRTI and for which the outcome—discharge or death—was known. The International Classification of Diseases codes used in this step were unlikely to capture any interaction between influenza and bacterial aetiologies of LRTI and the risk of death in people with viral–bacterial co-infections is probably higher than that in people with viral infections alone.^29^ This reanalysis substantially reduced the uncertainty around this adjustment and reduced the magnitude of the scalar used to adjust the fatal influenza attributable fraction (p 30).

The number of LRTI deaths attributable to influenza that we calculated was much lower than that in the 2018 report^30^ from Iuliano and colleagues, who estimated 290 000–650 000 seasonal influenza-associated respiratory deaths. Differences in the modelling strategy used account for a large amount of the variation in the estimates and have been explored as part of a modelling workshop between these research groups. Whereas Iuliano and colleagues' approach was designed to estimate any deaths that could be considered associated with influenza, our approach was designed to estimate the proportion of LRTI deaths that were attributable to (ie, caused by) influenza in our counterfactual framework. The GBD 2017 estimation strategy starts with an overall number of LRTI deaths and counterfactually attributes a fraction of these deaths to influenza. By contrast, Iuliano and colleagues used a time-series analysis to identify excess mortality among all respiratory-coded deaths (10th edition of the International Classification of Diseases codes J00–J99) during the influenza season and used these estimates to model annual influenza-associated respiratory deaths. Although their strategy allows for variation in burden by year and season, our approach was robust to influenza occurrence, association with LRTI episodes, and consistency between other causes of death, and estimates that were produced for every year, age, sex, and geographical region. Our approach has the major advantage of quantifying the potentially avertable influenza burden, and our results can be used to estimate the vaccine preventable burden and the burden preventable by other interventions.

Lafond and colleagues^22^ estimated the global frequency of paediatric influenza-associated hospitalisations. They used a similar approach to ours: starting with a systematic review of the proportion of respiratory infections positive for influenza among children younger than 18 years that resulted in hospitalisation, they then calculated the product of that proportion and the number of acute LRTI hospitalisation in 2010, and estimated that there were 870 000 (95% CI 610 000–1 237 000) influenza-associated hospitalisations among children younger than 5 years.^22^ This estimate is lower than ours (2 224 000 [95% UI 738 000–5 979 000]) but our estimates of the fraction of hospitalised LRTIs that are due to influenza are nearly identical (7·4%^22^ *vs* 6·9% in our study), which suggests that the main difference is in the overall estimates of LRTI hospitalisations among children younger than 5 years. Indeed, our estimate of LRTI hospitalisations of 32 211 000 (95% UI 18 073 000–52 112 000) is much larger than that used by Lafond and colleagues (11 751 000).^22^, ^31^ This example shows the effect of multiple estimated values in both studies and that rates of hospitalisations depend on not only the attributable fraction of LRTI due to influenza but also the overall availability and use of health care.

Our study had several limitations. The availability and quality of data are very important in any predictive regression model. There is a scarcity of data in several geographical regions, such as south Asia and sub-Saharan Africa for several of our models, particularly the models for LRTI mortality and hospitalisation. By contrast, there was good data coverage for LRTI incidence globally because of population representative surveys. The data that informed the attributable fraction of influenza were also sparse in some parts of the world, such as central Europe and central Asia. Strengthening of the capacity for detection of influenza has become a priority among global health organisations, largely with the aim of improving early detection systems, but expansion of routine surveillance could also help to improve global burden estimates.^32^ Our systematic review identified several reports from central-latitude locations in which influenza accounted for a substantial proportion of LRTI episodes.^33^ This finding suggests that influenza could be an important cause of LRTIs in tropical climates,^34^ an area of some debate centred on whether environmental suitability is necessary for seasonal influenza burden.

As mentioned previously, because of an absence of data, we assumed that the presence of influenza in a respiratory sample has the same risk of contributing to an LRTI episode among children younger than 5 years as among older children and adults. This assumption could be inaccurate when taking into account various immunological and biological factors, and circulating influenza strains. Changes in circulating influenza virus strains over time and space have been studied extensively to predict strains for seasonal vaccine development and for assessments of the risk of a pandemic.^35^ Our study attempted to combine all strains of influenza into a single burden estimate, and stratification by subtype, including influenza A, B, and pandemic H1N1, might reveal additional trends in influenza burden. However, the influenza burden has the potential to change substantially from year to year and from season to season.^36^ We deliberately chose to include only studies that were done over a full year to avoid biasing our estimates with data from studies done during the peak influenza season. We also chose to exclude studies that tested for pandemic H1N1 exclusively because we thought this would bias our estimates, but included studies that tested for H1N1 concurrently with seasonal influenza. These decisions contributed to stability over time in our estimates of the attributable fraction of influenza. Although our model probably failed to capture significant year-to-year variation in influenza burden, the purpose of this modelling approach was to minimise the effect of such variation in calculation of the attributable burden of influenza LRTI.

Although much of the public attention on influenza has centred around the global threat of a pandemic, and has rightly emphasised the potential effects of the virus in an increasingly interconnected global community, our results show that seasonal influenza contributes to a substantial burden of LRTIs. The best way to prevent influenza-specific LRTIs is vaccination or targeted prophylaxis, and we have previously showed that reduction of exposure to air pollution and tobacco smoke could have major effects on the risk of all LRTIs.^4^ The highest burden of influenza LRTIs is in low-income and middle-income countries, and thus additional data about influenza epidemiology in those countries are needed to guide decisions about the development of vaccines and appropriate vaccination strategies to prevent severe influenza illnesses.^37^ Efforts to develop improved influenza vaccines that could avert a large annual burden of LRTI episodes, deaths, and hospitalisations are needed.^38^, ^39^ A full understanding of the burden of seasonal influenza could contribute to improved understanding of the epidemiology of the virus and preparedness in case of another influenza pandemic.

For **exact values for these estimates** see http://ihmeuw.org/4o0
