# Supplementary material for: Mortality, morbidity, and hospitalisations due to influenza lower respiratory tract infections, 2017: an analysis for the Global Burden of Disease Study 2017
Source: Lancet Respir Med. 2019 Jan;7(1):69–89. doi: 10.1016/S2213-2600(18)30496-X (PMC6302221; doi:10.1016/S2213-2600(18)30496-X)
Supplement: Supplementary appendix [file mmc1.pdf]

THE LANCET
Respiratory Medicine

Supplementary appendix

This appendix formed part of the original submission and has been peer reviewed. We post it as supplied by the authors.

Supplement to: GBD 2017 Influenza Collaborators.Mortality, morbidity, and hospitalisations due to influenza lower respiratory tract infections, 2017: an analysis for the Global Burden of Disease Study 2017. *Lancet Respir Med* 2018; published online Dec 12. http://dx.doi.org/10.1016/S2213-2600(18)30496-X.

Supplementary material for: Assessing the mortality, morbidity, and hospitalisations due to influenza lower respiratory infections: an analysis from the Global Burden of Disease Study 2017

Additional information on modelling inputs and methods

Definition of lower respiratory infection. We used clinician-diagnosed pneumonia or bronchiolitis as our case definition for lower respiratory infections (LRI). We included ICD9 codes 073.0-073.6, 079.82, 466-469, 480-489, 513.0, and 770.0 and ICD10 codes A48.1, J09-J22, J85.1, P23-P23.9, and U04. LRI etiologies are modeled separately from overall LRI incidence and prevalence. The etiologies include influenza, respiratory syncytial virus (RSV), *Streptococcus pneumonia* (pneumococcal pneumonia), and *Haemophilus influenzae* type b (Hib) and are episodes of LRI where the aetiology is the causal pathogen in the infection.

LRI mortality model. Lower respiratory infection mortality was estimated in the Cause of Death Ensemble model (CODEm) platform.[1,2] CODEm is a Bayesian statistical model and uses spatial priors from a hierarchical structure to inform the mortality models. CODEm is based on five general principles: identifying all available data, maximising the comparability and quality of the dataset, developing a diverse set of plausible models, assessing the predictive validity of each plausible individual model and of ensemble models, and choosing the model or ensemble model with the best performance in out-of-sample predictive analysis. CODEm produces a large suite of models based on either cause fraction or mortality rate, uses linear and space-time Gaussian process regression (ST-GPR), and a covariate selection process. Each sub-model is evaluated using out-of-sample predictive validity. Thirty percent of the data are excluded from the initial model fits and 15% are used to evaluate component models and 15% used to build the ensembles. The sub-models are ranked using 15% of the data based on their out-of-sample predictive validity. The proportion weighting of the ensemble sub-models is evaluated using the remaining 15% of the hold-out data. This weighting scheme evaluates ensemble models that are built with ranked sub-models contributing proportionally more or fewer draws to the final ensemble. The final ensemble model is evaluated against other ensemble models using the same fit statistics (in-sample, out-of-sample root mean squared error and data coverage). Detailed information on this process can be found in Foreman et al 2012[3] and in the GBD 2016 Mortality and Causes of Death manuscript.[4]

Covariates are selected independently for each sub-model and the selection is based on an algorithm that captures biologically plausible relationships between the covariates and LRI mortality and provides a diversity of possible models (**Appendix Table 1**). Each model includes all combinations of covariates if the direction of effect is along the assumed direction and the coefficient is significant at the $p < 0{\cdot}05$ level. Also, if adding a higher level covariate changes the significance of a level one to non-significant or an implausible direction, it will be dropped from the set. The reason for this algorithm is to give priority and emphasis on covariates that are more causally and proximately related to LRI such as air pollution and malnutrition rather than more contextual and macro covariates such as education and income per capita.

Appendix Table 1. Covariates used in LRI mortality modelling. Table 1A is for children under 5 and Table 1B shows the covariates used for ages 5-95+. The *Level* is the associated strength of relationship between the covariate and LRI mortality, ranked from 1 (proximally related) to 3 (distally related). The direction is the forced direction of the association between the covariate and LRI mortality.

Table 1A. Covariates used in under 5 years model

Level	Covariate	Direction
1	Childhood stunting SEV	+
	Childhood underweight SEV	+
	Childhood wasted SEV	+
	Indoor air pollution	+
	Short gestation SEV	+
	Low weight gestation	+
	LRI summary exposure variable	+
	Second-hand smoking prevalence	+
	Antibiotics for LRI	-
	Hib vaccine coverage	-
	Pneumococcal conjugate vaccine coverage	-
2	Discontinued breastfeeding SEV	+
	Vitamin A deficiency	+
	Zinc deficiency	+
	DTP3 vaccine coverage	-
	Healthcare access and quality index	-
3	Outdoor air pollution ($PM_{2.5}$)	+
	Population density > 1000/km^2	+
	Sanitation SEV	+
	Handwashing	-
	LDI per capita	-
	Maternal education	-
	Socio-demographic Index	-

Table 1B. Covariates used in 5-95+ years model

Level	Covariate	Direction
1	Indoor air pollution	+
	LRI summary exposure variable	+
	Outdoor air pollution	+
	Secondhand smoking prevalence	+
	Smoking prevalence	+
2	DTP3 vaccine coverage	-
	Healthcare access and quality index	-
	Mean BMI	-
	Pneumococcal conjugate vaccine coverage	-
	Handwashing	+
3	Education years per capita	-
	LDI per capita	-
	Socio-demographic Index	-
	Alcohol consumption	+
	Sanitation summary exposure variable	+

LRI mortality is estimated for 23 age groups, 774 locations, both sexes, and every year from 1980-2016. We estimated LRI mortality separately for males and females and for children under 5 years and older than 5 years due to expected underlying differences in the risk of mortality between these age groups. Data-rich and data-poor geographic locations were modelled separately and these models were then hybridised for a global model. This was to maintain proper uncertainty in the models where trusted data on causes of death exist. LRI mortality estimates are then squeezed into an overall mortality envelope by age/sex/location/year in a process called CoDCorrect. This step is to ensure internal consistency among causes of death and that the sum of cause-specific mortality is the same as the estimated all-cause mortality.

Non-fatal LRI model. The non-fatal LRI burden, including incidence and prevalence, is modelled in DisMod-MR 2·1 (DisMod). DisMod is a Bayesian, hierarchical, age-integrating meta-regression tool that relates incidence, prevalence, recovery, and mortality. Input data are from a systematic literature review of cross-sectional and cohort studies, hospital inpatient and outpatient data (ICD9 Codes included 073·0-073·6, 079·82, 466-469, 480-489, 513·0, and 770·0. ICD10 codes included A48·1, J09-J22, J85·1, P23-P23·9, and U04), MarketScan healthcare utilization data (USA only), and population-representative surveys. To make the data more consistent for covariates and adjustments in the modelling process, we converted all incidence data to prevalence data using an average duration of illness of 7.8 days.

Input data are adjusted to our standard case definition. Data are adjusted by study-level binary covariates which describe if the source is a hospital or inpatient sample and if the data come from a self-reported survey. Self-reported prevalence of LRI symptoms from population-representative surveys such as the Demographic and Health Survey (DHS) and the Multiple Indicator Cluster Survey (MICS) is used. Our case definition from symptom-based prevalence estimates is children in the last two weeks with fever and cough with difficulty breathing and symptoms located in the chest and/or chest and nose. This is consistent with the WHO Integrated Management of Childhood Illness guidelines definition of pneumonia and with the DHS and MICS definition of acute respiratory infection (ARI).[5] We extracted the prevalence of children under 5 years old that had fever and cough with difficulty breathing and included an indicator for this less-specific definition.

Additional information on the modeling strategy and input data for LRI modeling in GBD 2017 can be found in the GBD 2017 Causes of Death manuscript and the Morbidity and Disability manuscript.[6–8]

Influenza attribution. We updated our systematic review of scientific literature for the proportion of LRI that tested positive for influenza to include all data from GBD 2016 and from studies published between January 1, 1990 and May 26, 2017. Studies published in English were extracted. The inclusion and exclusion criteria as well as the search strings were unchanged between GBD 2016 and GBD 2017 and was:

*("lower respiratory"[title/abstract] OR pneumonia[title/abstract]) AND (**2016/05/01[PDat] :** 2017/12/31[PDat]) AND (incidence OR prevalence OR epidemiology OR etiolog*[title/abstract] OR influenza[title/abstract] OR "respiratory syncytial virus"[title/abstract]) AND Humans[MeSH Terms] NOT(autoimmune[title/abstract] OR COPD [title/abstract] OR "cystic fibrosis"[title/abstract] OR Review[ptyp])*

A map of these input data sources can be found in **Appendix Figure 2.** Inclusion criteria were studies that had a sample size of at least 100, studies that were at least one year in duration, and

studies describing lower respiratory infections, pneumonia, or bronchiolitis as the case definition. During our updated literature review we identified 595 studies, of which 75 met our inclusion criteria and were extracted. Before our updated literature review completed for GBD 2017, there were 153 data sources used for influenza burden modeling in GBD 2016, the previous round of estimation. We excluded studies that described pandemic H1N1 influenza solely and studies that used influenza-like illness as the case definition. We assigned an age range based on the prevalence-weighted mean age of LRI in the appropriate year/sex/location if the ages of the study participants were not reported.

We modeled the proportion data using the meta-regression tool DisMod-MR to estimate the proportion of LRI cases that are positive for influenza by location/year/age/sex. We accounted for study-level covariates in our models such as PCR as the diagnostic technique, studies that investigated influenza exclusively, and studies from inpatient populations. The adjustments for PCR as the diagnostic technique and for inpatient sample populations were made before modeling occurred (**Appendix Figure 3**). The values of these adjustments can be found in **Appendix Table 2.** The list of sources used in influenza proportion modeling can be found in **Appendix Table 3.**

There is evidence of a causal association between influenza and LRIs among children under 5 when it is detected by RT-PCR from respiratory samples.[9] Based on these data[9] we estimated a population attributable fraction (PAF) of LRI episodes, hospitalisations, and deaths that were caused by influenza. This approach is a counterfactual analysis to determine the contribution of influenza to LRI. In this analysis, the counterfactual that is being estimated is the burden of LRI that would exist in the absence of influenza, in other words, the burden of LRI causally attributable to influenza. The attribution was based on the exposure and the risk of the outcome.

We estimated three related PAFs for non-mutually exclusive influenza LRI categories: a non-fatal PAF, a hospitalisation PAF, and a fatal PAF. The non-fatal attribution is the simplest and was defined as (**Appendix Figure 6A)**

$$PAF = \boldsymbol{Frequency\ (proportion)} * \left(\mathbf{1} - \frac{\mathbf{1}}{\boldsymbol{OR}}\right)$$

Where $Frequency$ is the modeled proportion of LRI episodes that test positive for influenza by PCR and varies by age, sex, year, and geography and *OR* is the odds ratio of an LRI episode given the presence of influenza in a respiratory tract sample (5.10, 95% Confidence Interval 3.19 to 8.14). [9] To account for the previously described frequency of influenza detection in hospitalised compared to non-hospitalised LRI episodes, we applied a constant scalar to determine the PAF for LRI hospitalisations which was determined by comparing the mean frequency of influenza detection in hospitalised compared to non-hospitalised sample populations in the proportion data from our literature review *(Hospital scalar*, **Appendix Table 2, Appendix Figure 6B***)*.

$$\text{PAF Hospitalisations} = Frequency\ (proportion) * \left(1 - \frac{1}{OR}\right) * \boldsymbol{Hospital\ scalar}$$

Finally, to account for the relative difference in the risk of mortality between bacterial and viral causes of LRIs, we modelled the ratio in case fatality of viral-to-bacterial ICD-coded hospital admissions (**Appendix page 22**). Hospital data from high and low-income countries were used in this analysis and

we estimated an age-specific curve for this relationship (**Appendix Table 4**). This scalar was applied to determine the attribution of influenza for fatal LRI outcomes (*Fatality scalar*, **Appendix Figure 6C**).

$$\text{PAF Fatal} = Frequency\ (proportion) * \left(1 - \frac{1}{OR}\right) * Hospital\ Scalar * \boldsymbol{Fatality\ Scalar}$$

The final number of LRI episodes, hospitalisations, and deaths attributable to influenza was the product of the relevant PAF and the overall number of episodes, hospitalisations, and deaths for each country, year, age, and sex.

Appendix Table 2. Adjustment factors for data that report the percentage of LRI episodes that tested positive for influenza.

These results suggest that PCR detects influenza about 25% more frequently than non-PCR diagnostic methods and that influenza is detected about 22% more frequently in non-hospitalised LRI episodes compared to hospitalized LRI episodes. The boxplots in Appendix Figure 1 show the values for the frequency of influenza in LRI episodes by diagnostic used and by hospital inpatient sample population.

Covariate	Adjustment
Non-PCR diagnostic	0.75 (0.62-0.89)
Inpatient population	0.78 (0.69-0.89)

Appendix Figure 1. Boxplots of the frequency of influenza detected in LRI episodes by diagnostic type used and by the sample population in the study.

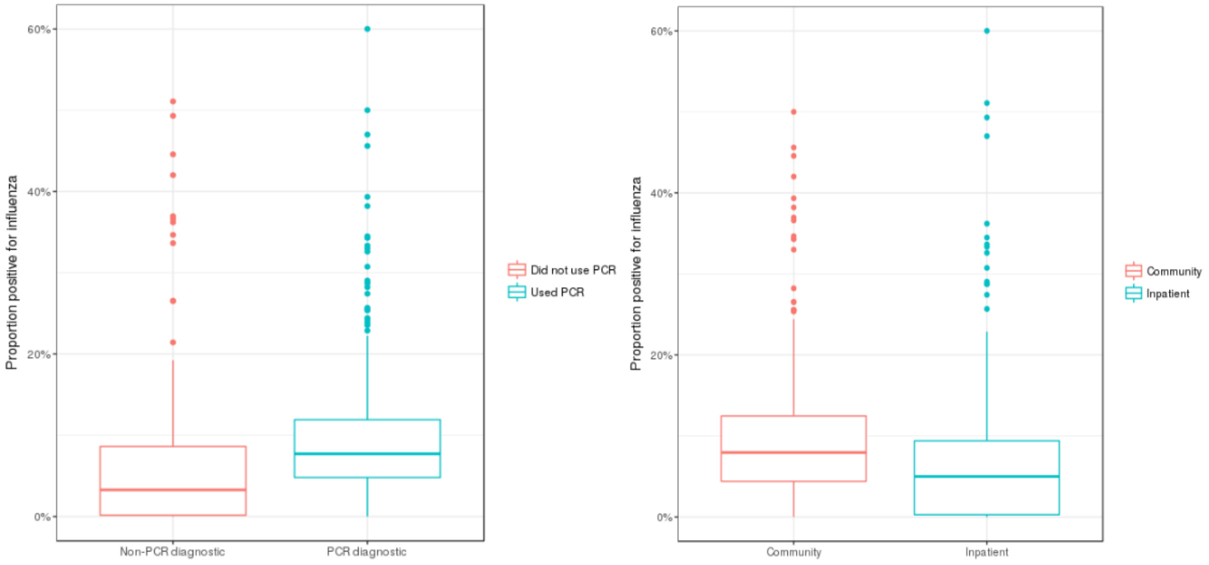

Appendix Table 3. Source list for influenza proportion modeling in GBD 2017 (201 sources).

Source	Location
Adegbola RA, Falade AG, Sam BE, Aidoo M, Baldeh I, Hazlett D, Whittle H, Greenwood BM, Mulholland EK. The etiology of pneumonia in malnourished and well-nourished Gambian children. Pediatr Infect Dis J. 1994; 13(11): 975-82.	The Gambia
Ali A, Khowaja AR, Bashir MZ, Aziz F, Mustafa S, Zaidi A. Role of human metapneumovirus, influenza A virus and respiratory syncytial virus in causing WHO-defined severe pneumonia in children in a developing country. PLoS One. 2013; 8(9): e74756.	Pakistan
Almirall J, Boixeda R, Bolíbar I, Bassa J, Sauca G, Vidal J, Serra-Prat M, Balanzó X, GEMPAC Study Group. Differences in the etiology of community-acquired pneumonia according to site of care: a population-based study. Respir Med. 2007; 101(10): 2168-75.	Spain
Arancibia F, Cortes CP, Valdés M, Cerda J, Hernández A, Soto L, Torres A. Importance of Legionella pneumophila in the etiology of severe community-acquired pneumonia in Santiago, Chile. Chest. 2014; 145(2): 290-6.	Chile
Aydemir Y, Aydemir O, Pekcan S, Ozdemir M. Value of multiplex PCR to determine the bacterial and viral aetiology of pneumonia in school-age children. Paediatr Int Child Health. 2017; 37(1): 29-34.	Turkey
Azziz-Baumgartner E, Cabrera AM, Cheng P-Y, Garcia E, Kusznierz G, Calli R, Baez C, Buyayisqui MP, Poyard E, Pérez E, Basurto-Davila R, Palekar R, Oliva O, Alencar AP, de Souza R, Santos T dos, Shay DK, Widdowson M-A, Breese J, Echenique H. Incidence of influenza-associated mortality and hospitalizations in Argentina during 2002-2009. Influenza Other Respir Viruses. 2013; 7(5): 710–7.	Argenti na
Baggett HC, Chittaganpitch M, Thamthitiwat S, Prapasiri P, Naorat S, Sawatwong P, Ditsungnoen D, Olsen SJ, Simmerman JM, Srisaengchai P, Chantra S, Peruski LF, Sawanpanyalert P, Maloney SA, Akarasewi P. Incidence and epidemiology of hospitalized influenza cases in rural Thailand during the influenza A (H1N1)pdm09 pandemic, 2009-2010. PLoS One. 2012; 7(11): e48609.	Thailan d
Barrett C, Ben-Shimol S, Greenberg D. Differences Between Radiologically Confirmed Pneumonia With and Without Pleural Fluid in Hospitalized Children Younger Than 5 Years in Southern Israel. Clin Pediatr (Phila). 2016; 55(10): 897-903.	Israel
Basnet S, Sharma A, Mathisen M, Shrestha PS, Ghimire RK, Shrestha DM, Valentiner-Branth P, Sommerfelt H, Strand TA. Predictors of duration and treatment failure of severe pneumonia in hospitalized young Nepalese children. PLoS One. 2015; 10(3): e0122052.	Nepal
Berg AS, Inchley CS, Aase A, Fjaerli HO, Bull R, Aaberge I, Leegaard TM, Nakstad B. Etiology of Pneumonia in a Pediatric Population with High Pneumococcal Vaccine Coverage: A Prospective Study. Pediatr Infect Dis J. 2016; 35(3): e69-75.	Norway
Berkley JA, Munywoki P, Ngama M, Kazungu S, Abwao J, Bett A, Lassauniére R, Kresfelder T, Cane PA, Venter M, Scott JAG, Nokes DJ. Viral etiology of severe pneumonia among Kenyan infants and children. JAMA. 2010; 303(20): 2051-7.	Kenya
Bhat N, Tokarz R, Jain K, Haq S, Weatherholtz R, Chandran A, Karron R, Reid R, Santosham M, O'Brien KL, Lipkin WI. A prospective study of agents associated with acute respiratory infection among young American Indian children. Pediatr Infect Dis J. 2013; 32(8): e324-33.	United States
Bicer S, Giray T, Col D, Erdag GC, Vitrinel A, Gurol Y, Çelik G, Kaspar C, Kucuk O. Virological and clinical characterizations of respiratory infections in hospitalized children. Ital J Pediatr. 2013; 22.	Turkey
Breiman RF, Cosmas L, Njenga M, Williamson J, Mott JA, Katz MA, Erdman DD, Schneider E, Oberste M, Neatherlin JC, Njuguna H, Ondari DM, Odero K, Okoth GO, Olack B, Wamola N, Montgomery JM, Fields BS, Feikin DR. Severe acute respiratory infection in children in a densely populated urban slum in Kenya, 2007-2011. BMC Infect Dis. 2015; 15: 95.	Kenya
Briones ML, Blanquer J, Ferrando D, Blasco ML, Gimeno C, Marín J. Assessment of analysis of urinary pneumococcal antigen by immunochromatography for etiologic diagnosis of community-acquired pneumonia in adults. Clin Vaccine Immunol. 2006; 13(10): 1092-7.	Spain
Budge PJ, Griffin MR, Edwards KM, Williams JV, Verastegui H, Hartinger SM, Mäusezahl D, Johnson M, Klemenc JM, Zhu Y, Gil AI, Lanata CF, Grigalva CG. Impact of home environment interventions on the risk of influenza-associated ARI in Andean children: observations from a prospective household-based cohort study. PLoS One. 2014; 9(3): e91247.	Peru
Calvo C, Garcia-Garcia ML, Sanchez-Dehesa R, Roman C, Tabares A, Pozo F, Casas I. Eight Year Prospective Study of Adenoviruses Infections in Hospitalized Children. Comparison with Other Respiratory Viruses. PLoS One. 2015; 10(7): e0132162.	Spain

Cao B, Song S-F, Bai L, Yin YD, Zhang Y-Y, Liu Y-M, Guo P, Wang C, Ren L-L, Wang J-W, Zhao F, Zhang J-Z, Gonzalez R. Viral and Mycoplasma pneumoniae community-acquired pneumonia and novel clinical outcome evaluation in ambulatory adult patients in China. Eur J Clin Microbiol Infect Dis. 2010; 29(11): 1443-8.	China
Cevey-Macherel M, Galetto-Lacour A, Gervaix A, Siegrist C-A, Bille J, Bescher-Ninet B, Kaiser L, Krahenbuhl J-D, Gehri M. Etiology of community-acquired pneumonia in hospitalized children based on WHO clinical guidelines. Eur J Pediatr. 2009; 168(12): 1429-36.	Switzerl and
Charles PGP, Grayson ML, Pierce RJP, Mayall BC, Fuller AJ, Stirling R, Hooy M, Korman TM, Holmes PW, Wright AA, Catton MG, Whitby M, Johnson B, Armstrong JG, Nimmo GR, Christiansen KJ, Waterer GW, Grayson L, Johnson P, Munckhof W, Looke D, Garske L, Playford G, Spelman D, Kotsimbos T, Holmes P, Korman T, Bardin P, Heath C, Birch C, Druce J, Ryan N, Irving L, Hart D. The etiology of community-acquired pneumonia in Australia: Why penicillin plus doxycycline or a macrolide is the most appropriate therapy. Clin Infect Dis. 2008; 46(10): 1513-21.	Australi a
Chen CJ, Lin PY, Tsai MH, Huang CG, Tsao KC, Wong KS, Chang LY, Chiu CH, Lin TY, Huang YC. Etiology of community-acquired pneumonia in hospitalized children in northern Taiwan. Pediatr Infect Dis J. 2012; 31(11): e196-201.	Taiwan
Chen K, Jia R, Li L, Yang C, Shi Y. The aetiology of community associated pneumonia in children in Nanjing, China and aetiological patterns associated with age and season. BMC Public Health. 2015; 113.	China
Chiu SC, Lin JH, Wang HC, Wu HS, Chang HW, Lin YC, Liu HF. Molecular epidemiologic and clinical characteristics of influenza B-associated complications among hospitalized patients during an outbreak in Taiwan. Int J Infect Dis. 2014; 94-100.	Taiwan
Choi S-H, Hong S-B, Ko G-B, Lee Y, Park HJ, Park S-Y, Moon SM, Cho O-H, Park K-H, Chong YP, Kim S-H, Huh JW, Sung H, Do K-H, Lee S-O, Kim M-N, Jeong J-Y, Lim C-M, Kim YS, Woo JH, Koh Y. Viral infection in patients with severe pneumonia requiring intensive care unit admission. Am J Respir Crit Care Med. 2012; 186(4): 325-32.	South Korea
Choi SH, Huh JW, Hong SB, Lee JY, Kim SH, Sung H, Do KH, Lee SO, Kim MN, Jeong JY, Lim CM, Kim YS, Woo JH, Koh Y. Clinical characteristics and outcomes of severe rhinovirus-associated pneumonia identified by bronchoscopic bronchoalveolar lavage in adults: comparison with severe influenza virus-associated pneumonia. J Clin Virol. 2015; 62: 41-7.	South Korea
Chorazy ML, Lebeck MG, McCarthy TA, Richter SS, Torner JC, Gray GC. Polymicrobial acute respiratory infections in a hospital-based pediatric population. Pediatr Infect Dis J. 2013; 32(5): 460-6.	United States
Cicek C, Arslan A, Karakus HS, Yalaz M, Saz EU, Pullukcu H, Cok G. [Prevalence and seasonal distribution of respiratory viruses in patients with acute respiratory tract infections, 2002-2014]. Mikrobiyol Bul. 2015; 49(2): 188-200.	Turkey
Cilla G, Oñate E, Perez-Yarza EG, Montes M, Vicente D, Perez-Trallero E. Viruses in community-acquired pneumonia in children aged less than 3 years old: High rate of viral coinfection. J Med Virol. 2008; 80(10): 1843-9.	Spain
Cillóniz C, Ewig S, Menéndez R, Ferrer M, Polverino E, Reyes S, Gabarrús A, Marcos MA, Cordoba J, Mensa J, Torres A. Bacterial co-infection with H1N1 infection in patients admitted with community acquired pneumonia. J Infect. 2012; 65(3): 223-30.	Spain
Cohen AL, Hellferscee O, Pretorius M, Treurnicht F, Walaza S, Madhi S, Groome M, Dawood H, Variava E, Kahn K, Wolter N, von Gottberg A, Tempia S, Venter M, Cohen C. Epidemiology of influenza virus types and subtypes in South Africa, 2009-2012. Emerg Infect Dis. 2014; 20(7): 1162–9.	South Africa
Cohen C, Moyes J, Tempia S, Groom M, Walaza S, Pretorius M, Dawood H, Chhagan M, Haffejee S, Variava E, Kahn K, Tshangela A, von Gottberg A, Wolter N, Cohen AL, Kgokong B, Venter M, Madhi SA. Severe influenza-associated respiratory infection in high HIV prevalence setting, South Africa, 2009-2011. Emerg Infect Dis. 2013; 19(11): 1766–74.	South Africa
Cohen C, Walaza S, Moyes J, Groome M, Tempia S, Pretorius M, Hellferscee O, Dawood H, Chhagan M, Naby F, Haffejee S, Variava E, Kahn K, Nzenze S, Tshangela A, von Gottberg A, Wolter N, Cohen AL, Kgokong B, Venter M, Madhi SA. Epidemiology of viral-associated acute lower respiratory tract infection among children <5 years of age in a high HIV prevalence setting, South Africa, 2009-2012. Pediatr Infect Dis J. 2015; 34(1): 66–72.	South Africa
Costa LF, Queiroz DA, Lopes da Silveira H, Bernardino Neto M, de Paula NT, Oliveira TF, Tolardo AL, Yokosawa J. Human rhinovirus and disease severity in children. Pediatrics. 2014; 133(2): e312-21.	Brazil
da Silva ER, Pitrez MC, Arruda E, Mattiello R, Sarria EE, de Paula FE, Proença-Modena JL, Delcaro LS, Cintra O, Jones MH, Ribeiro JD, Stein RT. Severe lower respiratory tract infection in infants and	Brazil

toddlers from a non-affluent population: viral etiology and co-detection as risk factors. BMC Infect Dis. 2013; 41.	
Dananché C, Sánchez Picot V, Bénet T, *et al.* Burden of Influenza in Less Than 5-Year-Old Children Admitted to Hospital with Pneumonia in Developing and Emerging Countries: A Descriptive, Multicenter Study. *Am J Trop Med Hyg* 2018; **98**: 1805–10.	Cambodia
Dananché C, Sánchez Picot V, Bénet T, *et al.* Burden of Influenza in Less Than 5-Year-Old Children Admitted to Hospital with Pneumonia in Developing and Emerging Countries: A Descriptive, Multicenter Study. *Am J Trop Med Hyg* 2018; **98**: 1805–10.	China
Dananché C, Sánchez Picot V, Bénet T, *et al.* Burden of Influenza in Less Than 5-Year-Old Children Admitted to Hospital with Pneumonia in Developing and Emerging Countries: A Descriptive, Multicenter Study. *Am J Trop Med Hyg* 2018; **98**: 1805–10.	Haiti
Dananché C, Sánchez Picot V, Bénet T, *et al.* Burden of Influenza in Less Than 5-Year-Old Children Admitted to Hospital with Pneumonia in Developing and Emerging Countries: A Descriptive, Multicenter Study. *Am J Trop Med Hyg* 2018; **98**: 1805–10.	India
Dananché C, Sánchez Picot V, Bénet T, *et al.* Burden of Influenza in Less Than 5-Year-Old Children Admitted to Hospital with Pneumonia in Developing and Emerging Countries: A Descriptive, Multicenter Study. *Am J Trop Med Hyg* 2018; **98**: 1805–10.	Madagascar
Dananché C, Sánchez Picot V, Bénet T, *et al.* Burden of Influenza in Less Than 5-Year-Old Children Admitted to Hospital with Pneumonia in Developing and Emerging Countries: A Descriptive, Multicenter Study. *Am J Trop Med Hyg* 2018; **98**: 1805–10.	Mali
Dananché C, Sánchez Picot V, Bénet T, *et al.* Burden of Influenza in Less Than 5-Year-Old Children Admitted to Hospital with Pneumonia in Developing and Emerging Countries: A Descriptive, Multicenter Study. *Am J Trop Med Hyg* 2018; **98**: 1805–10.	Mongolia
Dananché C, Sánchez Picot V, Bénet T, *et al.* Burden of Influenza in Less Than 5-Year-Old Children Admitted to Hospital with Pneumonia in Developing and Emerging Countries: A Descriptive, Multicenter Study. *Am J Trop Med Hyg* 2018; **98**: 1805–10.	Paraguay
Das D, Le Floch H, Houhou N, Epelboin L, Hausfater P, Khalil A, Ray P, Duval X, Claessens Y-E, Leport C, ESCAPED Study Group. Viruses detected by systematic multiplex polymerase chain reaction in adults with suspected community-acquired pneumonia attending emergency departments in France. Clin Microbiol Infect. 2015; 21(6): 608e1-8.	France
De Schutter I, De Wachter E, Malfroot A, Soetens O, Pierard D, Crokaert F, Verhaegen J. Microbiology of bronchoalveolar lavage fluid in children with acute nonresponding or recurrent community-acquired pneumonia: Identification of nontypeable haemophilus influenzae as a major pathogen. Clin Infect Dis. 2011; 52(12): 1437-44.	Belgium
del Valle Mendoza J, Cornejo-Tapia A, Weilg P, Verne E, Nazario-Fuertes R, Ugarte C, del Valle LJ, Pumarola T. Incidence of respiratory viruses in Peruvian children with acute respiratory infections. J Med Virol. 2015; 87(6): 917–24.	Peru
Díaz A, Barria P, Niederman M, Restrepo MI, Dreyse J, Fuentes G, Couble B, Saldias F. Etiology of community-acquired pneumonia in hospitalized patients in Chile: the increasing prevalence of respiratory viruses among classic pathogens. Chest. 2007; 131(3): 779-87.	Chile
Esposito S, Daleno C, Prunotto G, Scala A, Tagliabue C, Borzani I, Fossali E, Pelucchi C, Principi N. Impact of viral infections in children with community-acquired pneumonia: results of a study of 17 respiratory viruses. Influenza Other Respir Viruses. 2013; 7(1): 18-26.	Italy
Ewig S, Klapdor B, Pletz MW, Rohde G, Schütte H, Schaberg T, Bauer TT, Welte T, CAPNETZ study group. Nursing-home-acquired pneumonia in Germany: an 8-year prospective multicentre study. Thorax. 2012; 67(2): 132-8.	Germany
Feikin DR, Njenga MK, Bigogo G, Aura B, Aol G, Audi A, Jagero G, Muluare PO, Gikunju S, Nderitu L, Balish A, Winchell J, Schneider E, Erdman D, Oberste MS, Katz MA, Breiman RF. Etiology and Incidence of viral and bacterial acute respiratory illness among older children and adults in rural western Kenya, 2007-2010. PLoS One. 2012; 7(8): e43656.	Kenya
Feikin DR, Njenga MK, Bigogo G, Aura B, Aol G, Audi A, Jagero G, Muluare PO, Gikunju S, Nderitu L, Winchell JM, Schneider E, Erdman DD, Oberste MS, Katz MA, Breiman RF. Viral and bacterial causes of severe acute respiratory illness among children aged less than 5 years in a high malaria prevalence area of western Kenya, 2007-2010. Pediatr Infect Dis J. 2013; 32(1): e14-9.	Kenya
Feng L, Lai S, Li F, Ye X, Li S, Ren X, Zhang H, Li Z, Yu H, Yang W. [Viral etiologies of hospitalized pneumonia patients aged less than five years in six provinces, 2009-2012]. Chin J Epidemiol. 2014; 35(6): 646–9.	China

Feng L, Li Z, Zhao S, Nair H, Lai S, Xu W, Li M, Wu J, Ren L, Liu W, Yuan Z, Chen Y, Wang X, Zhao Z, Zhang H, Li F, Ye X, Li S, Feikin D, Yu H, Yang W. Viral etiologies of hospitalized acute lower respiratory infection patients in China, 2009-2013. PLoS One. 2014; 9(6): e99419.	China
Ferronato AE, Gilio AE, Ferraro AA, Paulis Md, Vieira SE. Etiological diagnosis reduces the use of antibiotics in infants with bronchiolitis. Clinics (Sao Paulo). 2012; 67(9): 1001-6.	Brazil
Forgie IM, O'Neill KP, Lloyd-Evans N, Leinonen M, Campbell H, Whittle HC, Greenwood BM. Etiology of acute lower respiratory tract infections in Gambian children: I Acute lower respiratory tract infections in infants presenting at the hospital. Pediatr Infect Dis J. 1991; 10(1): 33-41.	The Gambia
Forgie IM, O'Neill KP, Lloyd-Evans N, Leinonen M, Campbell H, Whittle HC, Greenwood BM. Etiology of acute lower respiratory tract infections in Gambian children: II Acute lower respiratory tract infection in children ages one to nine years presenting at the hospital. Pediatr Infect Dis J. 1991; 10(1): 42-7.	The Gambia
Garcia-Garcia ML, Calvo C, Pozo F, Villadangos PA, Perez-Brena P, Casas I. Spectrum of respiratory viruses in children with community-acquired pneumonia. Pediatr Infect Dis J. 2012; 31(8): 808-13.	Spain
Gowin E, Bartkowska-Sniatkowska A, Jonczyk-Potoczna K, Wysocka-Leszczynska J, Bobkowski W, Fichna P, Sobkowiak P, Mazur-Melewska K, Breborowicz A, Wysocki J, Januszkiewicz-Lewandowska D. Assessment of the Usefulness of Multiplex Real-Time PCR Tests in the Diagnostic and Therapeutic Process of Pneumonia in Hospitalized Children: A Single-Center Experience. Biomed Res Int. 2017; 2017: 8037963.	Poland
Grijalva CG, Zhu Y, Williams DJ, Self WH, Ampofo K, Pavia AT, Stockmann CR, McCullers J, Arnold SR, Wunderink RG, Anderson EJ, Lindstrom S, Fry AM, Foppa IM, Finelli L, Bramley AM, Jain S, Griffin MR, Edwards KM. Association Between Hospitalization With Community-Acquired Laboratory-Confirmed Influenza Pneumonia and Prior Receipt of Influenza Vaccination. JAMA. 2015; 314(14): 1488-97.	United States, Georgia
Grijalva CG, Zhu Y, Williams DJ, Self WH, Ampofo K, Pavia AT, Stockmann CR, McCullers J, Arnold SR, Wunderink RG, Anderson EJ, Lindstrom S, Fry AM, Foppa IM, Finelli L, Bramley AM, Jain S, Griffin MR, Edwards KM. Association Between Hospitalization With Community-Acquired Laboratory-Confirmed Influenza Pneumonia and Prior Receipt of Influenza Vaccination. JAMA. 2015; 314(14): 1488-97.	United States, Illinois
Grijalva CG, Zhu Y, Williams DJ, Self WH, Ampofo K, Pavia AT, Stockmann CR, McCullers J, Arnold SR, Wunderink RG, Anderson EJ, Lindstrom S, Fry AM, Foppa IM, Finelli L, Bramley AM, Jain S, Griffin MR, Edwards KM. Association Between Hospitalization With Community-Acquired Laboratory-Confirmed Influenza Pneumonia and Prior Receipt of Influenza Vaccination. JAMA. 2015; 314(14): 1488-97.	United States, Tenness ee
Guan WD, Gong XY, Mok CK, Chen TT, Wu SG, Pan SH, Cowling BJ, Yang ZF, Chen de H. Surveillance for seasonal influenza virus prevalence in hospitalized children with lower respiratory tract infection in Guangzhou, China during the post-pandemic era. PLoS One. 2015; 10(4): e0120983.	China
Guerrier G, Goyet S, Chheng ET, Rammaert B, Borand L, Te V, Try PL, Sareth R, Cavailler P, Mayaud C, Guillard B, Vong S, Buchy P, Tarantola A. Acute viral lower respiratory tract infections in Cambodian children: clinical and epidemiologic characteristics. Pediatr Infect Dis J. 2013; 32(1): e8-13.	Cambod ia
Guo RN, Zheng HZ, Huang LQ, Zhou Y, Zhang X, Liang CK, Lin JY, He JF, Zhang JQ. Epidemiologic and economic burden of influenza in the outpatient setting: a prospective study in a subtropical area of China. PLoS One. 2012; 7(7): e41403.	China
Gupta V, Dawood FS, Rai SK, Broor S, Wigh R, Mishra AC, Lafond K, Mott JA, Widdowson M-A, Lal RB, Krishnan A. Validity of clinical case definitions for influenza surveillance among hospitalized patients: results from a rural community in North India. Influenza Other Respir Viruses. 2013; 7(3): 321–9.	India
Hammitt LL, Kazungu S, Morpeth SC, Gibson DG, Mvera B, Brent AJ, Mwarumba S, Onyango CO, Bett A, Akech DO, Murdoch DR, Nokes DJ, Scott JAG. A preliminary study of pneumonia etiology among hospitalized children in Kenya. Clin Infect Dis. 2012; 54(Supp 2): S190-199.	Kenya
Hara K, Yahara K, Gotoh K, Nakazono Y, Kashiwagi T, Imamura Y, Hamada N, Khositsakulchai W, Sanchai T, Khantawa B, Tharavichitkul P, Maneekarn N, Sirisanthana T, Watanabe H. Clinical study concerning the relationship between community-acquired pneumonia and viral infection in northern Thailand. Intern Med. 2011; 50(9): 991-8.	Thailan d
Hartung TK, Chimbayo D, van Oosterhout JJG, Chikaonda T, van Doornum GJJ, Claas ECJ, Melchers WJG, Molyneux ME, Zijlstra EE. Etiology of suspected pneumonia in adults admitted to a high-dependency unit in Blantyre, Malawi. Am J Trop Med Hyg. 2011; 85(1): 105-12.	Malawi

Hasan K, Jolly P, Marquis G, Roy E, Podder G, Alam K, Huq F, Sack R. Viral etiology of pneumonia in a cohort of newborns till 24 months of age in Rural Mirzapur, Bangladesh. Scand J Infect Dis. 2006; 38(8): 690-5.	Bangladesh
Hasan R, Rhodes J, Thamthitiwat S, Olsen SJ, Prapasiri P, Naorat S, Chittaganpitch M, Henchaichon S, Dejsirilert S, Srisaengchai P, Sawatwong P, Jorakate P, Kaewpwan A, Fry AM, Erdman D, Chuananon S, Amornintapichet T, Maloney SA, Baggett HC. Incidence and etiology of acute lower respiratory tract infections in hospitalized children younger than 5 years in rural Thailand. Pediatr Infect Dis J. 2014; 33(2): e45-52.	Thailand
He Y, Lin GY, Wang Q, Cai XY, Zhang YH, Lin CX, Lu CD, Lu XD. A 3-year prospective study of the epidemiology of acute respiratory viral infections in hospitalized children in Shenzhen, China. Influenza Other Respir Viruses. 2014; 8(4): 443-51.	China
Henrickson KJ, Hoover S, Kehl KS, Hua W. National disease burden of respiratory viruses detected in children by polymerase chain reaction. Pediatr Infect Dis J. 2004; 23(1 Suppl): S11–8.	United States
Hernandez-Bou S, Novell CB, Alins JG, García-García JJ. Hospitalized children with influenza A H1N1 (2009) infection: a Spanish multicenter study. Pediatr Emerg Care. 2013; 29(1): 49-52.	Spain
Herrera-Rodríguez DH, de la Hoz F, Mariño C, Ramírez E. [Respiratory virus in children aged less than 10 years old suffering from respiratory infection in the Hospital Militar Central in Bogota from 2000-2001]. Rev Salud Publica (Bogota). 2007; 9(4): 576–86.	Colombia
Hijazi Z, Pacsa A, El-Gharbawy F, Chugh TD, Essa S, El Shazli A, Abd El-Salam R. Acute lower respiratory tract infections in children in Kuwait. Ann Trop Paediatr. 1997; 17(2): 127-34.	Kuwait
Hoffmann J, Rabezanahary H, Randriamarotia M, Ratsimbasoa A, Najjar J, Vernet G, Contamin B, Paranhos-BaccalA G. Viral and atypical bacterial etiology of acute respiratory infections in children under 5 years old living in a rural tropical area of Madagascar. PLoS One. 2012; 7(8): e43666.	Madagascar
Holter JC, Muller F, Bjorang O, Samdal HH, Marthinsen JB, Jenum PA, Ueland T, Froland SS, Aukrust P, Husebye E, Heggelund L. Etiology of community-acquired pneumonia and diagnostic yields of microbiological methods: a 3-year prospective study in Norway. BMC Infect Dis. 2015; 15: 64.	Norway
Homaira N, Luby SP, Petri WA, Vainionpaa R, Rahman M, Hossain K, Snider CB, Rahman M, Alamgir ASM, Zesmin F, Alam M, Gurley ES, Zaman RU, Azim T, Erdman DD, Fry AM, Bresee J, Widdowson M-A, Haque R, Azziz-Baumgartner E. Incidence of respiratory virus-associated pneumonia in urban poor young children of Dhaka, Bangladesh, 2009-2011. PLoS One. 2012; 7(2): e32056.	Bangladesh
Hon KL, Leung E, Hung E, Tang J, Chow CM, Leung TF, Cheung KL, Ng PC. Premorbid factors and outcome associated with respiratory virus infections in a pediatric intensive care unit. Pediatr Pulmonol. 2008; 43(3): 275-80.	Hong Kong
Honkinen M, Lahti E, Österback R, Ruuskanen O, Waris M. Viruses and bacteria in sputum samples of children with community-acquired pneumonia. Clin Microbiol Infect. 2012; 18(3): 300-7.	Finland
Hoo Lee J, Hyung Kim Y. Comparison of clinical characteristics between healthcare-associated pneumonia and community-acquired pneumonia in patients admitted to secondary hospitals. Braz J Infect Dis. 2012; 16(4): 321-8.	South Korea
Huang G, Yu D, Mao N, Zhu Z, Zhang H, Jiang Z, Li H, Zhang Y, Shi J, Zhang S, Wang X, Xu W. Viral etiology of acute respiratory infection in Gansu Province, China, 2011. PLoS One. 2013; 8(5): e64254.	China
Huo X, Qin Y, Qi X, Zu R, Tang F, Li L, Hu Z, Zhu F. Surveillance of 16 respiratory viruses in patients with influenza-like illness in Nanjing, China. J Med Virol. 2012; 84(12): 1980-4.	China
Ishiguro T, Takayanagi N, Yamaguchi S, Yamakawa H, Nakamoto K, Takaku Y, Miyahara Y, Kagiyama N, Kurashima K, Yanagisawa T, Sugita Y. Etiology and factors contributing to the severity and mortality of community-acquired pneumonia. Intern Med. 2013; 52(3): 317-24.	Japan
Jain S, Self WH, Wunderink RG, Fakhran S, Balk R, Bramley AM, Reed C, Grijalva CG, Anderson EJ, Courtney DM, Chappell JD, Qi C, Hart EM, Carroll F, Trabue C, Donnelly HK, Williams DJ, Zhu Y, Arnold SR, Ampofo K, Waterer GW, Levine M, Lindstrom S, Winchell JM, Katz JM, Erdman D, Schneider E, Hicks LA, McCullers JA, Pavia AT, Edwards KM, Finelli L, CDC EPIC Study Team. Community-Acquired Pneumonia Requiring Hospitalization among U.S. Adults. N Engl J Med. 2015; 373(5): 415–27.	United States
Johansson N, Kalin M, Tiveljung-Lindell A, Giske CG, Hedlund J. Etiology of Community-Acquired pneumonia: Increased microbiological yield with new diagnostic methods. Clin Infect Dis. 2010; 50(2): 202-9.	Sweden
John TJ, Cherian T, Steinhoff MC, Simoes EA, John M. Etiology of acute respiratory infections in children in tropical southern India. Rev Infect Dis. 1991; 13 Suppl 6: S463-9.	India

Ju X, Fang Q, Zhang J, Xu A, Liang L, Ke C. Viral etiology of influenza-like illnesses in Huizhou, China, from 2011 to 2013. Arch Virol. 2014; 159(8): 2003-10.	China
Kelly MS, Smieja M, Luinstra K, Wirth KE, Goldfarb DM, Steenhoff AP, Arscott-Mills T, Cunningham CK, Boiditswe S, Sethomo W, Shah SS, Finalle R, Feemster KA. Association of respiratory viruses with outcomes of severe childhood pneumonia in Botswana. PLoS One. 2015; 10(5): e0126593.	Botswana
Kelly PM, Kotsimbos T, Reynolds A, Wood-Baker R, Hancox B, Brown SGA, Holmes M, Simpson G, Bowler S, Waterer G, Irving LB, Jenkins C, Thompson PJ, Cheng AC. FluCAN 2009: initial results from sentinel surveillance for adult influenza and pneumonia in eight Australian hospitals. Med J Aust. 2011; 194(4): 169–74.	Australia
Khor CS, Sam IC, Hooi PS, Quek KF, Chan YF. Epidemiology and seasonality of respiratory viral infections in hospitalized children in Kuala Lumpur, Malaysia: a retrospective study of 27 years. BMC Pediatr. 2012; 32.	Malaysia
Kim H-J, Choi SM, Lee J, Park YS, Lee C-H, Yim J-J, Yoo C-G, Kim YW, Han SK, Lee S-M. Respiratory virus of severe pneumonia in South Korea: Prevalence and clinical implications. PLoS One. 2018; 13(6): e0198902.	South Korea
Kim JK, Jeon JS, Kim JW, Rheem I. Epidemiology of respiratory viral infection using multiplex rt-PCR in Cheonan, Korea (2006-2010). J Microbiol Biotechnol. 2013; 23(2): 267-73.	South Korea
Koksal I, Bayraktar O, Yilmaz G, Caylan R, Aydin K, Sucu N, Ozlu T, Bulbul Y, Oztuna F, Ozcan DI, OguS C, Tekeli E, Kaya A, Ayaz C, Dagli CE, Yildiz O, Oymak FS, Kalkan A, Muz MH, Turgut H, Fisekci F, Heper Y, Uzaslan E. Etiological agents of community-acquired pneumonia in adult patients in Turkey; a multicentric, cross-sectional study. Tuberk Toraks. 2010; 58(2): 119-27.	Turkey
Kwofie TB, Anane YA, Nkrumah B, Annan A, Nguah SB, Owusu M. Respiratory viruses in children hospitalized for acute lower respiratory tract infection in Ghana. Virol J. 2012; 78.	Ghana
Laguna-Torres VA, Sánchez-Largaespada JF, Lorenzana I, Forshey B, Aguilar P, Jimenez M, Parrales E, Rodriguez F, García J, Jimenez I, Rivera M, Perez J, Sovero M, Rios J, Gamero ME, Halsey ES, Kochel TJ. Influenza and other respiratory viruses in three Central American countries. Influenza Other Respir Viruses. 2011; 5(2): 123–34.	El Salvador
Laguna-Torres VA, Sánchez-Largaespada JF, Lorenzana I, Forshey B, Aguilar P, Jimenez M, Parrales E, Rodriguez F, García J, Jimenez I, Rivera M, Perez J, Sovero M, Rios J, Gamero ME, Halsey ES, Kochel TJ. Influenza and other respiratory viruses in three Central American countries. Influenza Other Respir Viruses. 2011; 5(2): 123–34.	Honduras
Laguna-Torres VA, Sánchez-Largaespada JF, Lorenzana I, Forshey B, Aguilar P, Jimenez M, Parrales E, Rodriguez F, García J, Jimenez I, Rivera M, Perez J, Sovero M, Rios J, Gamero ME, Halsey ES, Kochel TJ. Influenza and other respiratory viruses in three Central American countries. Influenza Other Respir Viruses. 2011; 5(2): 123–34.	Nicaragua
Lahti E, Peltola V, Waris M, Virkki R, Rantakokko-Jalava K, Jalava J, Eerola E, Ruuskanen O. Induced sputum in the diagnosis of childhood community-acquired pneumonia. Thorax. 2009; 64(3): 252-7.	Finland
Laurent C, Dugue AE, Brouard J, Nimal D, Dina J, Parienti JJ, Vabret A. Viral epidemiology and severity of respiratory infections in infants in 2009: a prospective study. Pediatr Infect Dis J. 2012; 31(8): 827-31.	France
Lee JE, Choe K-W, Lee SW. Clinical and radiological characteristics of 2009 H1N1 influenza associated pneumonia in young male adults. Yonsei Med J. 2013; 54(4): 927-34.	South Korea
Li H, Wei Q, Tan A, Wang L. Epidemiological analysis of respiratory viral etiology for influenza-like illness during 2010 in Zhuhai, China. Virol J. 2013; 143.	China
Li QH, Gao WJ, Li JY, Shi LA, Hao XJ, Ge SW, An SH. [Detection of respiratory viruses in children with acute lower respiratory tract infection: an analysis of 5,150 children]. Chin J Contemp Pediatr. 2016; 18(1): 51-4.	China
Lieberman D, Shimoni A, Shemer-Avni Y, Keren-Naos A, Shtainberg R, Lieberman D. Respiratory viruses in adults with community-acquired pneumonia. Chest. 2010; 138(4): 811-6.	Israel
Lin Y, Fu Y, Xu M, Su L, Cao L, Xu J, Cheng X. Evaluation of a PCR/ESI-MS platform to identify respiratory viruses from nasopharyngeal aspirates. J Med Virol. 2015; 87.0(11): 1867-71.	China
Lindblade KA, Arvelo W, Gray J, Estevez A, Frenkel G, Reyes L, Moscoso F, Moir JC, Fry AM, Olsen SJ. A comparison of the epidemiology and clinical presentation of seasonal influenza A and 2009 pandemic influenza A (H1N1) in Guatemala. PLoS One. 2010; 5(12): e15826.	Guatemala
Litwin CM, Bosley JG. Seasonality and prevalence of respiratory pathogens detected by multiplex PCR at a tertiary care medical center. Arch Virol. 2014; 159(1): 65-72.	United States

Liu WK, Liu Q, Chen de H, Liang HX, Chen XK, Chen MX, Qiu SY, Yang ZY, Zhou R. Epidemiology of acute respiratory infections in children in Guangzhou: a three-year study. PLoS One. 2014; 9(5): e96674.	China
Liu YF, Gao Y, Chen MF, Cao B, Yang XH, Wei L. Etiological analysis and predictive diagnostic model building of community-acquired pneumonia in adult outpatients in Beijing, China. BMC Infect Dis. 2013; 309.	China
Lu G, Li J, Xie Z, Liu C, Guo L, Vernet G, Shen K, Wang J. Human metapneumovirus associated with community-acquired pneumonia in children in Beijing, China. J Med Virol. 2013; 85(1): 138-43.	China
Lynfield R, Davey R, Dwyer DE, Losso MH, Wentworth D, Cozzi-Lepri A, Herman-Lamin K, Cholewinska G, David D, Kuetter S, Ternesgen Z, Uyeki TM, Lane HC, Lundgren J, Neaton JD. Outcomes of influenza A(H1N1)pdm09 virus infection: results from two international cohort studies. PLoS One. 2014; 9(7): e101785.	United States
Marcone DN, Durand LO, Azziz-Baumgartner E, Vidaurreta S, Ekstrom J, Carballal G, Echavarria M. Incidence of viral respiratory infections in a prospective cohort of outpatient and hospitalized children aged ≤5 years and its associated cost in Buenos Aires, Argentina. BMC Infect Dis. 2015; 447.	Argenti na
Marcone DN, Ellis A, Videla C, Ekstrom J, Ricarte C, Carballal G, Vidaurreta SM, Echavarría M. Viral etiology of acute respiratory infections in hospitalized and outpatient children in Buenos Aires, Argentina. Pediatr Infect Dis J. 2013; 32(3): e105-10.	Argenti na
Mardy S, Ly S, Heng S, Vong S, Huch C, Nora C, Asgari N, Miller M, Bergeri I, Rehmet S, Veasna D, Zhou W, Kasai T, Touch S, Buchy P. Influenza activity in Cambodia during 2006-2008. BMC Infect Dis. 2009; 168.	Cambod ia
Martín-Loeches I, Bermejo-Martin JF, Vallés J, Granada R, Vidaur L, Vergara-Serrano JC, Martín M, Figueira JC, Sirvent JM, Blanquer J, Suarez D, Artigas A, Torres A, Diaz E, Rodriguez A, SEMICYUC/REIPI/CIBERES H1N1 Working Group. Macrolide-based regimens in absence of bacterial co-infection in critically ill H1N1 patients with primary viral pneumonia. Intensive Care Med. 2013; 39(4): 693-702.	Spain
Maruyama T, Gabazza EC, Morser J, Takagi T, D'Alessandro-Gabazza C, Hirohata S, Nakayama S, Ramirez AY, Fujiwara A, Naito M, Nishikubo K, Yuda H, Yoshida M, Takei Y, Taguchi O. Community-acquired pneumonia and nursing home-acquired pneumonia in the very elderly patients. Respir Med. 2010; 104(4): 584-92.	Japan
Maruyama T, Niederman MS, Kobayashi T, Kobayashi H, Takagi T, D'Alessandro-Gabazza CN, Fujimoto H, Gil Bernabe P, Hirohata S, Nakayama S, Nishikubo K, Yuda H, Yamaguchi A, Gabazza EC, Noguchi T, Takei Y, Taguchi O. A prospective comparison of nursing home-acquired pneumonia with hospital-acquired pneumonia in non-intubated elderly. Respir Med. 2008; 102(9): 1287-95.	Japan
Mathisen M, Basnet S, Sharma A, Shrestha PS, Sharma BN, Valentiner-Branth P, Sommerfelt H, Strand TA. RNA viruses in young Nepalese children hospitalized with severe pneumonia. Pediatr Infect Dis J. 2011; 30(12): 1032-6.	Nepal
Mathisen M, Strand TA, Sharma BN, Chandyo RK, Valentiner-Branth P, Basnet S, Adhikari RK, Hvidsten D, Shrestha PS, Sommerfelt H. RNA viruses in community-acquired childhood pneumonia in semi-urban Nepal; a cross-sectional study. BMC Med. 2009; 7: 35.	Nepal
Mathisen M, Strand TA, Valentiner-Branth P, Chandyo RK, Basnet S, Sharma BN, Adhikari RK, Hvidsten D, Shrestha PS, Sommerfelt H. Respiratory viruses in nepalese children with and without pneumonia: a case-control study. Pediatr Infect Dis J. 2010; 29(8): 731-5.	Nepal
Mathur S, Dubey T, Kulshrestha M, Agarwal H, Mathur G, Mathur A, Mathur S, Prakash P, Rajpurohit V, Gehlot G, Tak P. Clinical profile and mortality among novel influenza A (H1N1) infected patients: 2009-2010 Jodhpur, Rajasthan pandemic. J Assoc Physicians India. 2013; 61(9): 627-32.	India
McMorrow ML, Emukule GO, Njuguna HN, Bigogo G, Montgomery JM, Nyawanda B, Audi A, Breiman RF, Katz MA, Cosmas L, Waiboci LW, Duque J, Widdowson MA, Mott JA. The Unrecognized Burden of Influenza in Young Kenyan Children, 2008-2012. PLoS One. 2015; 10(9): e0138272.	Kenya
Mermond S, Zurawski V, D'Ortenzio E, Driscoll AJ, DeLuca AN, Deloria-Knoll M, Moisi JC, Murdoch DR, Missotte I, Besson-Leaud L, Chevalier C, Debarnot V, Feray F, Noireterre S, Duparc B, Fresnais F, O'Connor O, Dupont-Rouzeyrol M, Levine OS. Lower respiratory infections among hospitalized children in New Caledonia: a pilot study for the Pneumonia Etiology Research for Child Health project. Clin Infect Dis. 2012; S180-9.	Oceania
Moore HC, de Klerk N, Keil AD, Smith DW, Blyth CC, Richmond P, Lehmann D. Use of data linkage to investigate the aetiology of acute lower respiratory infection hospitalisations in children. J Paediatr Child Health. 2012; 48(6): 520–8.	Australi a

Moreno L, Krishnan JA, Duran P, Ferrero F. Development and validation of a clinical prediction rule to distinguish bacterial from viral pneumonia in children. Pediatr Pulmonol. 2006; 41(4): 331-7.	Argentina
Morimoto K, Suzuki M, Ishifuji T, Yaegashi M, Asoh N, Hamashige N, Abe M, Aoshima M, Ariyoshi K, Adult Pneumonia Study Group-Japan (APSG-J). The burden and etiology of community-onset pneumonia in the aging Japanese population: a multicenter prospective study. PLoS One. 2015; 10(3): e0122247.	Japan
Narvaez-Rivera RM, Rendon A, Salinas-Carmona MC, Rosas-Taraco AG. Soluble RAGE as a severity marker in community acquired pneumonia associated sepsis. BMC Infect Dis. 2012; 12: 15.	Mexico
Nascimento-Carvalho CM, Araujo-Neto CA, Ruuskanen O. Association between bacterial infection and radiologically confirmed pneumonia among children. Pediatr Infect Dis J. 2015; 34(5): 490-3.	Brazil
Nascimento-Carvalho CM, Ribeiro CT, Viriato D, Souza AL, Cardoso MRA, Barral A, Araujo-Neto CA, Oliveira JR, Sobral LS, Saukkoriipi A, Paldanius M, Leinonen M, Vainionpaa R, Ruuskanen O. The role of respiratory viral infections among children hospitalized for community-acquired pneumonia in a developing country. Pediatr Infect Dis J. 2008; 27(10): 939-41.	Brazil
Nasreen S, Luby SP, Brooks WA, Homaira N, Al Mamun A, Bhuiyan MU, Rahman M, Ahmed D, Abedin J, Rahman M, Alamgir AS, Fry AM, Streatfield PK, Rahman A, Bresee J, Widdowson MA, Azziz-Baumgartner E. Population-based incidence of severe acute respiratory virus infections among children aged <5 years in rural Bangladesh, June-October 2010. PLoS One. 2014; 9(2): e89978.	Bangladesh
Ndegwa LK, Katz MA, McCormick K, Nganga Z, Mungai A, Emukule G, Kollmann MKHM, Mayieka L, Otieno J, Breiman RF, Mott JA, Ellingson K. Surveillance for respiratory health care-associated infections among inpatients in 3 Kenyan hospitals, 2010-2012. Am J Infect Control. 2014; 42(9): 985–90.	Kenya
Ngeow YF, Weil AF, Khairullah NS, Yusof MY, Luam L, Gaydos C, Quinn TC. Young Malaysian children with lower respiratory tract infections show low incidence of chlamydial infection. J Paediatr Child Health. 1997; 33(5): 422-5.	Malaysia
O'Callaghan-Gordo C, Bassat Q, Morais L, Díez-Padrisa N, Machevo S, Nhampossa T, Nhalungo D, Sanz S, Quintó L, Alonso PL, Roca A. Etiology and epidemiology of viral pneumonia among hospitalized children in rural Mozambique: a malaria endemic area with high prevalence of human immunodeficiency virus. Pediatr Infect Dis J. 2011; 30(1): 39-44.	Mozambique
Olsen SJ, Thamthitiwat S, Baggett HC, Maloney S, Peruski LF, Chantra S, Chittaganpitch M, Sawanpanyalert P, Fry AM, Simmerman JM, Peret TCT, Erdman D, Benson R, Thacker L, Tondella ML, Winchell J, Fields B, Talkington D, Nicholson WL, Ungchusak K, Dowell SF. Incidence of respiratory pathogens in persons hospitalized with pneumonia in two provinces in Thailand. Epidemiol Infect. 2010; 138(12): 1811-22.	Thailand
Onyango CO, Njeru R, Kazungu S, Achilla R, Bulimo W, Welch SR, Cane PA, Gunson RN, Hammitt LL, Scott JAG, Berkley JA, Nokes DJ. Influenza surveillance among children with pneumonia admitted to a district hospital in coastal Kenya, 2007-2010. J Infect Dis. 2012; 206(Suppl 1): S61-67.	Kenya
Ouédraogo S, Traoré B, Nene Bi ZAB, Yonli FT, Kima D, Bonané P, Congo L, Traoré RO, Yé D, Marguet C, Plantier J-C, Vabret A, Gueudin M. Viral etiology of respiratory tract infections in children at the pediatric hospital in Ouagadougou (Burkina Faso). PLoS One. 2014; 9(10): e110435.	Burkina Faso
Palmu AA, Saukkoriipi A, Snellman M, Jokinen J, Torkko P, Ziegler T, Kaijalainen T, Hausdorff WP, Verlant V, Kilpi TM. Incidence and etiology of community-acquired pneumonia in the elderly in a prospective population-based study. Scand J Infect Dis. 2014; 46(4): 250-9.	Finland
Papenburg J, Hamelin ME, Ouhoummane N, Carbonneau J, Ouakki M, Raymond F, Robitaille L, Corbeil J, Caouette G, Frenette L, De Serres G, Boivin G. Comparison of risk factors for human metapneumovirus and respiratory syncytial virus disease severity in young children. J Infect Dis. 2012; 206(2): 178-89.	Canada
Peng Y, Shu C, Fu Z, Li QB, Liu Z, Yan L. [Pathogen detection of 1 613 cases of hospitalized children with community acquired pneumonia]. Chin J Contemp Pediatr. 2015; 17.0(11): 1193-9.	China
Piralam B, Tomczyk SM, Rhodes JC, Thamthitiwat S, Gregory CJ, Olsen SJ, Praphasiri P, Sawatwong P, Naorat S, Chantra S, Areerat P, Hurst CP, Moore MR, Muangchana C, Baggett HC. Incidence of Pneumococcal Pneumonia Among Adults in Rural Thailand, 2006-2011: Implications for Pneumococcal Vaccine Considerations. Am J Trop Med Hyg. 2015; 93(6): 1140-7.	Thailand
Pretorius MA, Madhi SA, Cohen C, Naidoo D, Groome M, Moyes J, Buys A, Walaza S, Dawood H, Chhagan M, Haffjee S, Kahn K, Puren A, Venter M. Respiratory viral coinfections identified by a 10-plex real-time reverse-transcription polymerase chain reaction assay in patients hospitalized with severe acute respiratory illness--South Africa, 2009-2010. J Infect Dis. 2012; S159-65.	South Africa

Puig-Barbera J, Mira-Iglesias A, Tortajada-Girbes M, Lopez-Labrador FX, Belenguer-Varea A, Carballido-Fernandez M, Carbonell-Franco E, Carratala-Munuera C, Limon-Ramirez R, Mollar-Maseres J, Del Carmen Otero-Reigada M, Schwarz-Chavarri G, Tuells J, Gil-Guillen V, Valencia Hospital Network for the Study of Influenza and Respiratory Viruses Disease. Effectiveness of influenza vaccination programme in preventing hospital admissions, Valencia, 2014/15 early results. Euro Surveill. 2015; 20(8).	Spain
Qu J-X, Gu L, Pu Z-H, Yu X-M, Liu Y-M, Li R, Wang Y-M, Cao B, Wang C, Beijing Network for Adult Community-Acquired Pneumonia (BNACAP). Viral etiology of community-acquired pneumonia among adolescents and adults with mild or moderate severity and its relation to age and severity. BMC Infect Dis. 2015; 89.	China
Quandelacy TM, Viboud C, Charu V, Lipsitch M, Goldstein E. Age- and sex-related risk factors for influenza-associated mortality in the United States between 1997-2007. Am J Epidemiol. 2014; 179(2): 156-67.	United States
Radin JM, Katz MA, Tempia S, Talla Nzussouo N, Davis R, Duque J, Adedeji A, Adjabeng MJ, Ampofo WK, Ayele W, Bakamutumaho B, Barakat A, Cohen AL, Cohen C, Dalhatu IT, Daouda C, Dueger E, Francisco M, Heraud J-M, Jima D, Kabanda A, Kadjo H, Kandeel A, Bi Shamamba SK, Kasolo F, Kronmann KC, Mazaba Liwewe ML, Lutwama JJ, Matonya M, Mmbaga V, Mott JA, Muhimpundu MA, Muthoka P, Njuguna H, Randrianasolo L, Refaey S, Sanders C, Talaat M, Theo A, Valente F, Venter M, Woodfill C, Bresee J, Moen A, Widdowson M-A. Influenza surveillance in 15 countries in Africa, 2006-2010. J Infect Dis. 2012; S14–21.	Angola
Radin JM, Katz MA, Tempia S, Talla Nzussouo N, Davis R, Duque J, Adedeji A, Adjabeng MJ, Ampofo WK, Ayele W, Bakamutumaho B, Barakat A, Cohen AL, Cohen C, Dalhatu IT, Daouda C, Dueger E, Francisco M, Heraud J-M, Jima D, Kabanda A, Kadjo H, Kandeel A, Bi Shamamba SK, Kasolo F, Kronmann KC, Mazaba Liwewe ML, Lutwama JJ, Matonya M, Mmbaga V, Mott JA, Muhimpundu MA, Muthoka P, Njuguna H, Randrianasolo L, Refaey S, Sanders C, Talaat M, Theo A, Valente F, Venter M, Woodfill C, Bresee J, Moen A, Widdowson M-A. Influenza surveillance in 15 countries in Africa, 2006-2010. J Infect Dis. 2012; S14–21.	Cote d'Ivoire
Radin JM, Katz MA, Tempia S, Talla Nzussouo N, Davis R, Duque J, Adedeji A, Adjabeng MJ, Ampofo WK, Ayele W, Bakamutumaho B, Barakat A, Cohen AL, Cohen C, Dalhatu IT, Daouda C, Dueger E, Francisco M, Heraud J-M, Jima D, Kabanda A, Kadjo H, Kandeel A, Bi Shamamba SK, Kasolo F, Kronmann KC, Mazaba Liwewe ML, Lutwama JJ, Matonya M, Mmbaga V, Mott JA, Muhimpundu MA, Muthoka P, Njuguna H, Randrianasolo L, Refaey S, Sanders C, Talaat M, Theo A, Valente F, Venter M, Woodfill C, Bresee J, Moen A, Widdowson M-A. Influenza surveillance in 15 countries in Africa, 2006-2010. J Infect Dis. 2012; S14–21.	Democratic Republic of the Congo
Radin JM, Katz MA, Tempia S, Talla Nzussouo N, Davis R, Duque J, Adedeji A, Adjabeng MJ, Ampofo WK, Ayele W, Bakamutumaho B, Barakat A, Cohen AL, Cohen C, Dalhatu IT, Daouda C, Dueger E, Francisco M, Heraud J-M, Jima D, Kabanda A, Kadjo H, Kandeel A, Bi Shamamba SK, Kasolo F, Kronmann KC, Mazaba Liwewe ML, Lutwama JJ, Matonya M, Mmbaga V, Mott JA, Muhimpundu MA, Muthoka P, Njuguna H, Randrianasolo L, Refaey S, Sanders C, Talaat M, Theo A, Valente F, Venter M, Woodfill C, Bresee J, Moen A, Widdowson M-A. Influenza surveillance in 15 countries in Africa, 2006-2010. J Infect Dis. 2012; S14–21.	Egypt
Radin JM, Katz MA, Tempia S, Talla Nzussouo N, Davis R, Duque J, Adedeji A, Adjabeng MJ, Ampofo WK, Ayele W, Bakamutumaho B, Barakat A, Cohen AL, Cohen C, Dalhatu IT, Daouda C, Dueger E, Francisco M, Heraud J-M, Jima D, Kabanda A, Kadjo H, Kandeel A, Bi Shamamba SK, Kasolo F, Kronmann KC, Mazaba Liwewe ML, Lutwama JJ, Matonya M, Mmbaga V, Mott JA, Muhimpundu MA, Muthoka P, Njuguna H, Randrianasolo L, Refaey S, Sanders C, Talaat M, Theo A, Valente F, Venter M, Woodfill C, Bresee J, Moen A, Widdowson M-A. Influenza surveillance in 15 countries in Africa, 2006-2010. J Infect Dis. 2012; S14–21.	Ethiopia
Radin JM, Katz MA, Tempia S, Talla Nzussouo N, Davis R, Duque J, Adedeji A, Adjabeng MJ, Ampofo WK, Ayele W, Bakamutumaho B, Barakat A, Cohen AL, Cohen C, Dalhatu IT, Daouda C, Dueger E, Francisco M, Heraud J-M, Jima D, Kabanda A, Kadjo H, Kandeel A, Bi Shamamba SK, Kasolo F, Kronmann KC, Mazaba Liwewe ML, Lutwama JJ, Matonya M, Mmbaga V, Mott JA, Muhimpundu MA, Muthoka P, Njuguna H, Randrianasolo L, Refaey S, Sanders C, Talaat M, Theo A, Valente F, Venter M, Woodfill C, Bresee J, Moen A, Widdowson M-A. Influenza surveillance in 15 countries in Africa, 2006-2010. J Infect Dis. 2012; S14–21.	Ghana
Radin JM, Katz MA, Tempia S, Talla Nzussouo N, Davis R, Duque J, Adedeji A, Adjabeng MJ, Ampofo WK, Ayele W, Bakamutumaho B, Barakat A, Cohen AL, Cohen C, Dalhatu IT, Daouda C, Dueger E,	Kenya

Francisco M, Heraud J-M, Jima D, Kabanda A, Kadjo H, Kandeel A, Bi Shamamba SK, Kasolo F, Kronmann KC, Mazaba Liwewe ML, Lutwama JJ, Matonya M, Mmbaga V, Mott JA, Muhimpundu MA, Muthoka P, Njuguna H, Randrianasolo L, Refaey S, Sanders C, Talaat M, Theo A, Valente F, Venter M, Woodfill C, Bresee J, Moen A, Widdowson M-A. Influenza surveillance in 15 countries in Africa, 2006-2010. J Infect Dis. 2012; S14–21.	
Radin JM, Katz MA, Tempia S, Talla Nzussouo N, Davis R, Duque J, Adedeji A, Adjabeng MJ, Ampofo WK, Ayele W, Bakamutumaho B, Barakat A, Cohen AL, Cohen C, Dalhatu IT, Daouda C, Dueger E, Francisco M, Heraud J-M, Jima D, Kabanda A, Kadjo H, Kandeel A, Bi Shamamba SK, Kasolo F, Kronmann KC, Mazaba Liwewe ML, Lutwama JJ, Matonya M, Mmbaga V, Mott JA, Muhimpundu MA, Muthoka P, Njuguna H, Randrianasolo L, Refaey S, Sanders C, Talaat M, Theo A, Valente F, Venter M, Woodfill C, Bresee J, Moen A, Widdowson M-A. Influenza surveillance in 15 countries in Africa, 2006-2010. J Infect Dis. 2012; S14–21.	Morocco
Radin JM, Katz MA, Tempia S, Talla Nzussouo N, Davis R, Duque J, Adedeji A, Adjabeng MJ, Ampofo WK, Ayele W, Bakamutumaho B, Barakat A, Cohen AL, Cohen C, Dalhatu IT, Daouda C, Dueger E, Francisco M, Heraud J-M, Jima D, Kabanda A, Kadjo H, Kandeel A, Bi Shamamba SK, Kasolo F, Kronmann KC, Mazaba Liwewe ML, Lutwama JJ, Matonya M, Mmbaga V, Mott JA, Muhimpundu MA, Muthoka P, Njuguna H, Randrianasolo L, Refaey S, Sanders C, Talaat M, Theo A, Valente F, Venter M, Woodfill C, Bresee J, Moen A, Widdowson M-A. Influenza surveillance in 15 countries in Africa, 2006-2010. J Infect Dis. 2012; S14–21.	Nigeria
Radin JM, Katz MA, Tempia S, Talla Nzussouo N, Davis R, Duque J, Adedeji A, Adjabeng MJ, Ampofo WK, Ayele W, Bakamutumaho B, Barakat A, Cohen AL, Cohen C, Dalhatu IT, Daouda C, Dueger E, Francisco M, Heraud J-M, Jima D, Kabanda A, Kadjo H, Kandeel A, Bi Shamamba SK, Kasolo F, Kronmann KC, Mazaba Liwewe ML, Lutwama JJ, Matonya M, Mmbaga V, Mott JA, Muhimpundu MA, Muthoka P, Njuguna H, Randrianasolo L, Refaey S, Sanders C, Talaat M, Theo A, Valente F, Venter M, Woodfill C, Bresee J, Moen A, Widdowson M-A. Influenza surveillance in 15 countries in Africa, 2006-2010. J Infect Dis. 2012; S14–21.	Rwanda
Radin JM, Katz MA, Tempia S, Talla Nzussouo N, Davis R, Duque J, Adedeji A, Adjabeng MJ, Ampofo WK, Ayele W, Bakamutumaho B, Barakat A, Cohen AL, Cohen C, Dalhatu IT, Daouda C, Dueger E, Francisco M, Heraud J-M, Jima D, Kabanda A, Kadjo H, Kandeel A, Bi Shamamba SK, Kasolo F, Kronmann KC, Mazaba Liwewe ML, Lutwama JJ, Matonya M, Mmbaga V, Mott JA, Muhimpundu MA, Muthoka P, Njuguna H, Randrianasolo L, Refaey S, Sanders C, Talaat M, Theo A, Valente F, Venter M, Woodfill C, Bresee J, Moen A, Widdowson M-A. Influenza surveillance in 15 countries in Africa, 2006-2010. J Infect Dis. 2012; S14–21.	South Africa
Radin JM, Katz MA, Tempia S, Talla Nzussouo N, Davis R, Duque J, Adedeji A, Adjabeng MJ, Ampofo WK, Ayele W, Bakamutumaho B, Barakat A, Cohen AL, Cohen C, Dalhatu IT, Daouda C, Dueger E, Francisco M, Heraud J-M, Jima D, Kabanda A, Kadjo H, Kandeel A, Bi Shamamba SK, Kasolo F, Kronmann KC, Mazaba Liwewe ML, Lutwama JJ, Matonya M, Mmbaga V, Mott JA, Muhimpundu MA, Muthoka P, Njuguna H, Randrianasolo L, Refaey S, Sanders C, Talaat M, Theo A, Valente F, Venter M, Woodfill C, Bresee J, Moen A, Widdowson M-A. Influenza surveillance in 15 countries in Africa, 2006-2010. J Infect Dis. 2012; S14–21.	Tanzania
Radin JM, Katz MA, Tempia S, Talla Nzussouo N, Davis R, Duque J, Adedeji A, Adjabeng MJ, Ampofo WK, Ayele W, Bakamutumaho B, Barakat A, Cohen AL, Cohen C, Dalhatu IT, Daouda C, Dueger E, Francisco M, Heraud J-M, Jima D, Kabanda A, Kadjo H, Kandeel A, Bi Shamamba SK, Kasolo F, Kronmann KC, Mazaba Liwewe ML, Lutwama JJ, Matonya M, Mmbaga V, Mott JA, Muhimpundu MA, Muthoka P, Njuguna H, Randrianasolo L, Refaey S, Sanders C, Talaat M, Theo A, Valente F, Venter M, Woodfill C, Bresee J, Moen A, Widdowson M-A. Influenza surveillance in 15 countries in Africa, 2006-2010. J Infect Dis. 2012; S14–21.	Uganda
Radin JM, Katz MA, Tempia S, Talla Nzussouo N, Davis R, Duque J, Adedeji A, Adjabeng MJ, Ampofo WK, Ayele W, Bakamutumaho B, Barakat A, Cohen AL, Cohen C, Dalhatu IT, Daouda C, Dueger E, Francisco M, Heraud J-M, Jima D, Kabanda A, Kadjo H, Kandeel A, Bi Shamamba SK, Kasolo F, Kronmann KC, Mazaba Liwewe ML, Lutwama JJ, Matonya M, Mmbaga V, Mott JA, Muhimpundu MA, Muthoka P, Njuguna H, Randrianasolo L, Refaey S, Sanders C, Talaat M, Theo A, Valente F, Venter M, Woodfill C, Bresee J, Moen A, Widdowson M-A. Influenza surveillance in 15 countries in Africa, 2006-2010. J Infect Dis. 2012; S14–21.	Zambia
Rahman M, Huq F, Sack DA, Butler T, Azad AK, Alam A, Nahar N, Islam M. Acute lower respiratory tract infections in hospitalized patients with diarrhea in Dhaka, Bangladesh. Rev Infect Dis. 1990; 12(Suppl 8): S899-906.	Bangladesh

Reechaipichitkul W, Thavornpitak Y, Sutra S. Burden of adult pneumonia in Thailand: a nationwide hospital admission data 2010. J Med Assoc Thai. 2014; 97(3): 283-92.	Thailand
Rioseco Z ML, Riquelme O R, Riquelme O M, Inzunza P C, Oyarzún G P, Agüero O Y, Ferrés G M, Vial C P, Fasce P R, Torres M A. [Viral etiology of community acquired pneumonia among adults admitted to a general hospital]. Rev Med Chil. 2012; 140(8): 984-9.	Chile
Salvador García C, Moreno Docón A, Piñero JA, Alfayate Miguelez S, Iborra Bendicho MA. [Aetiology of bronchiolitis in hospitalised children in South-East Spain]. An Pediatr (Barc). 2012; 77(6): 386-90.	Spain
Samransamruajkit R, Hiranrat T, Chieochansin T, Sritippayawan S, Deerojanawong J, Prapphal N, Poovorawan Y. Prevalence, clinical presentations and complications among hospitalized children with influenza pneumonia. Jpn J Infect Dis. 2008; 61(6): 446-9.	Thailand
Sangil A, Calbo E, Robles A, Benet S, Viladot ME, Pascual V, Cuchí E, Pérez J, Barreiro B, Sánchez B, Torres J, Canales L, De Marcos JA, Garau J. Aetiology of community-acquired pneumonia among adults in an H1N1 pandemic year: the role of respiratory viruses. Eur J Clin Microbiol Infect Dis. 2012; 31(10): 2765-72.	Spain
Schlaudecker EP, Heck JP, Macintyre ET, Martinez R, Dodd CN, McNeal MM, Staat MA, Heck JE, Steinhoff MC. Etiology and seasonality of viral respiratory infections in rural Honduran children. Pediatr Infect Dis J. 2012; 31(11): 1113-8.	Honduras
Self WH, Williams DJ, Zhu Y, Ampofo K, Pavia AT, Chappell JD, Hymas WC, Stockmann C, Bramley AM, Schneider E, Erdman D, Finelli L, Jain S, Edwards KM, Grijalva CG. Respiratory Viral Detection in Children and Adults: Comparing Asymptomatic Controls and Patients With Community-Acquired Pneumonia. J Infect Dis. 2016; 213(4): 584-91.	United States
Sentilhes AC, Choumlivong K, Celhay O, Sisouk T, Phonekeo D, Vongphrachanh P, Brey P, Buchy P. Respiratory virus infections in hospitalized children and adults in Lao PDR. Influenza Other Respir Viruses. 2013; 7(6): 1070-8.	Laos
Shafik CF, Mohareb EW, Yassin AS, Amin MA, El Kholy A, El-Karaksy H, Youssef FG. Viral etiologies of lower respiratory tract infections among Egyptian children under five years of age. BMC Infect Dis. 2012; 350.	Egypt
Shibli F, Flatau E, Nitzan O, Chazan B, Edelstein H, Raz R, Colodner R, Blondheim O. Etiology of community-acquired pneumonia in hospitalized patients in Northern Israel. Isr Med Assoc J. 2010; 12(8): 477-82.	Israel
Shu LH, Xu JJ, Wang S, Zhong HQ, Dong XY, Jiang K, Zhang HY, Xiong Q, Wang C, Sun T, Sun C, Lu Q. [Distribution of pathogenic microorganisms and its relationship with clinical features in children with community-acquired pneumonia]. Chin J Contemp Pediatr. 2015; 17(10): 1056-61.	China
Song JY, Lee JS, Wie SH, Kim HY, Lee J, Seo YB, Jeong HW, Kim SW, Lee SH, Park KH, Noh JY, Choi WS, Cheong HJ, Kim WJ. Prospective cohort study on the effectiveness of influenza and pneumococcal vaccines in preventing pneumonia development and hospitalization. Clin Vaccine Immunol. 2015; 22(2): 229-34.	South Korea
Spichak TV, Yatsyshina SB, Đšatosova LĐš, Đšim SS, Korppi MO. Is the role of rhinoviruses as causative agents of pediatric community-acquired pneumonia over-estimated?. <i>Eur J Pediatr</i>. 2016; 175(12): 1951â€“8.	Russia
Storms AD, Kusriastuti R, Misriyah S, Praptiningsih CY, Amalya M, Lafond KE, Samaan G, Triada R, Iuliano AD, Ester M, Sidjabat R, Chittenden K, Vogel R, Widdowson MA, Mahoney F, Uyeki TM. The East Jakarta Project: surveillance for highly pathogenic avian influenza A(H5N1) and seasonal influenza viruses in patients seeking care for respiratory disease, Jakarta, Indonesia, October 2011-September 2012. Epidemiol Infect. 2015; 143(16): 3394–404.	Indonesia
Sundaram ME, Meece JK, Sifakis F, Gasser RA, Belongia EA. Medically attended respiratory syncytial virus infections in adults aged >= 50 years: clinical characteristics and outcomes. Clin Infect Dis. 2014; 58(3): 342-9.	United States
Sung RYT, Chan PKS, Tsen T, Li AM, Lam WY, Yeung ACM, Nelson EAS. Identification of viral and atypical bacterial pathogens in children hospitalized with acute respiratory infections in Hong Kong by multiplex PCR assays. J Med Virol. 2009; 81(1): 153-9.	Hong Kong
Sung RYT, Cheng AFB, Chan RCK, Tam JS, Oppenheimer SJ. Epidemiology and Etiology of Pneumonia in Children in Hong Kong. Clin Infect Dis. 1993; 17(5): 894-6.	Hong Kong
Suzuki A, Lupisan S, Furuse Y, Fuji N, Saito M, Tamaki R, Galang H, Sombrero L, Mondoy M, Aniceto R, Olveda R, Oshitani H. Respiratory viruses from hospitalized children with severe pneumonia in the Philippines. BMC Infect Dis. 2012; 12: 267.	Philippines

Tajima T, Nakayama E, Kondo Y, Hirai F, Ito H, Iitsuka T, Momomura M, Kutsuma H, Kodaka Y, Funaki N, Yanagawa Y, Ubukata K. Etiology and clinical study of community-acquired pneumonia in 157 hospitalized children. J Infect Chemother. 2006; 12(6): 372-9.	Japan
Tecu C, Mihai ME, Alexandrescu VI, Or??eanu D, Zapucioiu C, Ivanciuc AE, Necula G, Lupulescu E, Chiri?? D, Pi?igoi D. Single and multipathogen viral infections in hospitalized children with acute respiratory infections. Roum Arch Microbiol Immunol. 2013; 72(4): 242-9.	Romani a
Teeratakulpisarn J, Pientong C, Ekalaksananan T, Ruangsiripiyakul H, Uppala R. Rhinovirus infection in children hospitalized with acute bronchiolitis and its impact on subsequent wheezing or asthma: a comparison of etiologies. Asian Pac J Allergy Immunol. 2014; 32(3): 226–34.	Thailan d
Tessmer A, Welte T, Schmidt-Ott R, Eberle S, Barten G, Suttorp N, Schaberg T, CAPNETZ study group. Influenza vaccination is associated with reduced severity of community-acquired pneumonia. Eur Respir J. 2011; 38(1): 147-53.	German y
Tran DN, Trinh QD, Pham NTK, Vu MP, Ha MT, Nguyen TQN, Okitsu S, Hayakawa S, Mizuguchi M, Ushijima H. Clinical and epidemiological characteristics of acute respiratory virus infections in Vietnamese children. Epidemiol Infect. 2015; 1–10.	Vietnam
Vallés J, Martin-Loeches I, Torres A, Diaz E, Seijas I, López MJ, Garro P, Castillo C, Garnacho-Montero J, Martin Mdel M, de la Torre MV, Olaechea P, Cilloniz C, Almirall J, García F, Jiménez R, Seoane E, Soriano C, Mesalles E, Posada P. Epidemiology, antibiotic therapy and clinical outcomes of healthcare-associated pneumonia in critically ill patients: a Spanish cohort study. Intensive Care Med. 2014; 40(4): 572-81.	Spain
Verani JR, McCracken J, Arvelo W, Estevez A, Lopez MR, Reyes L, Moir JC, Bernart C, Moscoso F, Gray J, Olsen SJ, Lindblade KA. Surveillance for hospitalized acute respiratory infection in Guatemala. PLoS One. 2013; 8(12): e83600.	Guatem ala
Viasus D, Marinescu C, Villoslada A, Cordero E, Galvez-Acebal J, Farinas MC, Gracia-Ahufinger I, Fernandez-Navarro A, Niubo J, Ortega L, Munez-Rubio E, Romero-Gomez MP, Carratala J. Community-acquired pneumonia during the first post-pandemic influenza season: a prospective, multicentre cohort study. J Infect. 2013; 67(3): 185-93.	Spain
Von Baum H, Schweiger B, Welte T, Marre R, Suttorp N, Pletz MWR, Ewig S, CAPNETZ Study Group. How deadly is seasonal influenza-associated pneumonia? The German Competence Network for Community-Acquired Pneumonia. Eur Respir J. 2011; 37(5): 1151-7.	German y
Vong S, Guillard B, Borand L, Rammaert B, Goyet S, Te V, Lorn Try P, Hem S, Rith S, Ly S, Cavailler P, Mayaud C, Buchy P. Acute lower respiratory infections in 5 year -old hospitalized patients in Cambodia, a low-income tropical country: clinical characteristics and pathogenic etiology. BMC Infect Dis. 2013; 97.	Cambod ia
Weigl JA, Puppe W, Belke O, Neusüss J, Bagci F, Schmitt HJ. Population-based incidence of severe pneumonia in children in Kiel, Germany. Klin Padiatr. 2005; 217(4): 211–9.	German y
Wiemken T, Peyrani P, Bryant K, Kelley RR, Summersgill J, Arnold F, Carrico R, McKinney WP, Jonsson C, Carrico K, Ramirez J. Incidence of respiratory viruses in patients with community-acquired pneumonia admitted to the intensive care unit: results from the Severe Influenza Pneumonia Surveillance (SIPS) project. Eur J Clin Microbiol Infect Dis. 2013; 32(5): 705-10.	United States
Williams JV, Edwards KM, Weinberg GA, Griffin MR, Hall CB, Zhu Y, Szilagyi PG, Wang CK, Yang C-F, Silva D, Ye D, Spaete RR, Crowe JE Jr. Population-based incidence of human metapneumovirus infection among hospitalized children. J Infect Dis. 2010; 201(12): 1890-8.	Nepal
Wolf DG, Greenberg D, Shemer-Avni Y, Givon-Lavi N, Bar-Ziv J, Dagan R. Association of human metapneumovirus with radiologically diagnosed community-acquired alveolar pneumonia in young children. J Pediatr. 2010; 156(1): 115-20.	Israel
Woolpert T, Brodine S, Lemus H, Waalen J, Blair P, Faix D. Determination of clinical and demographic predictors of laboratory-confirmed influenza with subtype analysis. BMC Infect Dis. 2012; 129.	United States
Yoshida LM, Suzuki M, Yamamoto T, Nguyen HA, Nguyen CD, Nguyen AT, Oishi K, Vu TD, Le TH, Le MQ, Yanai H, Kilgore PE, Dang DA, Ariyoshi K. Viral pathogens associated with acute respiratory infections in central vietnamese children. Pediatr Infect Dis J. 2010; 29(1): 75–7.	Vietnam
Yoshida LM, Suzuki M, Yamamoto T, Nguyen HA, Nguyen CD, Nguyen AT, Oishi K, Vu TD, Le TH, Le MQ, Yanai H, Kilgore PE, Dang DA, Ariyoshi K. Viral pathogens associated with acute respiratory infections in central vietnamese children. Pediatr Infect Dis J. 2010; 29(1): 75–7.	Belgium
Yu X, Lu R, Wang Z, Zhu N, Wang W, Julian D, Chris B, Lu J, Lv J, Tan W. Etiology and clinical characterization of respiratory virus infections in adult patients attending an emergency department in Beijing. PLoS One. 2012; 7(2): e32174.	China

Zhang C, Zhu N, Xie Z, Lu R, He B, Liu C, Ma X, Tan W. Viral etiology and clinical profiles of children with severe acute respiratory infections in China. PLoS One. 2013; 8(8): e72606.	China
Zhang PJ, Cao B, Li XL, Liang LR, Yang SG, Gu L, Xu Z, Hu K, Zhang HY, Yan XX, Huang WB, Chen W, Zhang JX, Li LJ, Wang C. Risk factors for adult death due to 2009 pandemic influenza A (H1N1) virus infection: a 2151 severe and critical cases analysis. Chin Med J (Engl). 2013; 126(12): 2222-8.	China
Zhang Q, Guo Z, Bai Z, MacDonald NE. A 4 year prospective study to determine risk factors for severe community acquired pneumonia in children in southern China. Pediatr Pulmonol. 2013; 48(4): 390-7.	China
Zhang Q, MacDonald NE, Guo Z. Vaccine preventable community-acquired pneumonia in hospitalized children in Northwest China. Pediatr Infect Dis J. 2011; 30(1): 7-10.	China
Zimmerman RK, Rinaldo CR, Nowalk MP, Gk B, Thompson MG, Moehling KK, Bullotta A, Wisniewski S. Influenza and other respiratory virus infections in outpatients with medically attended acute respiratory infection during the 2011-12 influenza season. Influenza Other Respir Viruses. 2014; 8(4): 397-405.	Pennsylvania
Zolotusca L, Jorgensen P, Popovici O, Pistol A, Popovici F, Widdowson MA, Alexandrescu V, Ivanciuc A, Cheng PY, Gross D, Brown CS, Mott JA. Risk factors associated with fatal influenza, Romania, October 2009-May 2011. Influenza Other Respir Viruses. 2014; 8(1): 8-12.	Romania

We used a counterfactual modeling strategy to estimate population attributable fractions (PAFs). The PAF represents the relative reduction in LRI mortality if there were no exposure to influenza. As LRIs can be caused by multiple pathogens and the pathogens may co-infect, aetiologies in GBD are not mutually exclusive and the sum of all aetiologies does not necessarily equal 100%. We did not attribute etiologies to neonatal pneumonia deaths due to a dearth of reliable data in this age group. We calculated uncertainty of our PAF estimates from 1,000 draws of each parameter using normal distributions in log space.

As the case-fatality of viral causes of pneumonia is lower than for bacterial causes, we adjusted for differential case-fatality by determining the etiological fractions for mortality attributable to influenza (**Appendix Figure 4**). We measured the etiologic fractions by applying a relative case-fatality adjustment based on in-hospital case-fatality, which we coded to specific pneumonia etiologies. Hospital admissions data of this type were limited to data from the following 12

countries: Austria, Brazil, Chile, China, Ecuador, Italy, Kenya, Mexico, New Zealand, Philippines, Portugal, and the United States. ICD 10 codes J10 (J10.0-J10.08), J11 (J11.0-J11.08) and J12 were categorized as viral pneumonia diagnoses and ICD 10 codes J13, J14, and J15 were categorized as bacterial pneumonia. We generated the pooled estimate of the case-fatality differential between bacterial and viral etiologies using DisMod-MR to determine an age pattern for this ratio (**Appendix Figure 4**).

More detail on the LRI modeling approach can be found elsewhere, including the GBD 2017 Causes of Death manuscript, GBD 2017 morbidity and mortality manuscript, and the GBD 2016 LRI capstone manuscript. LRI hospitalizations were modeled as a proportion of inpatient admissions to LRI incidence by age/sex/year/geography. Hospital admissions data came from 47 sources which are listed in **Appendix Table 4.**

Appendix Figure 2. The number of data sources used in the influenza proportion model. Gray shading indicates that a country does not have any data sources used in modeling.

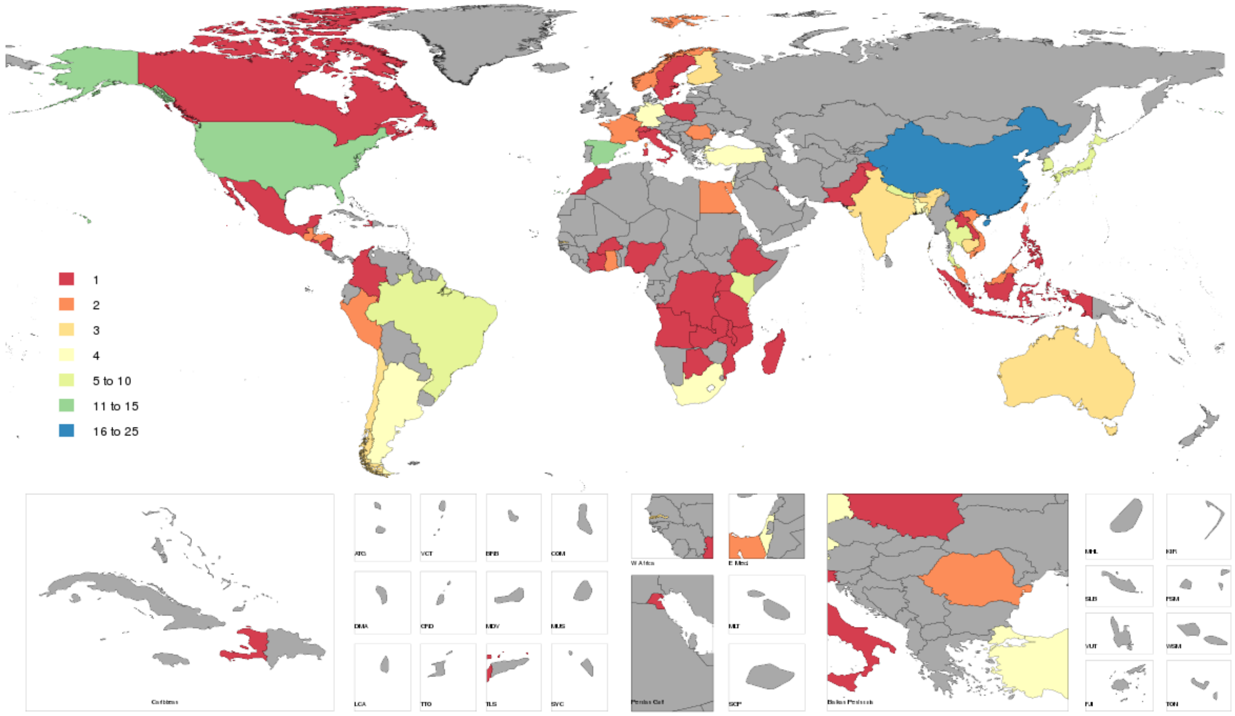

Appendix Figure 3. The mean, adjusted percentage of LRI episodes that test positive for influenza *before* modeling. A) Geographic distribution, B) Age distribution, C) Time distribution. Gray in the map indicates that a country does not have any data used in modeling.

A)

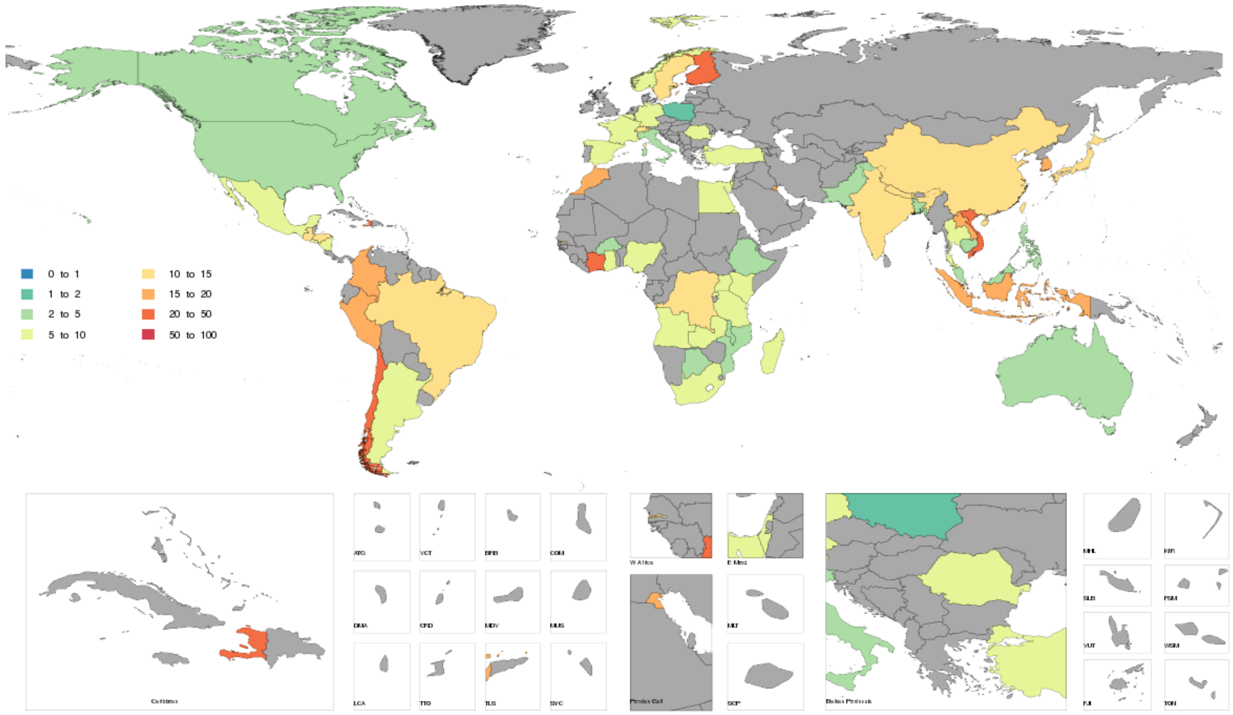

B)

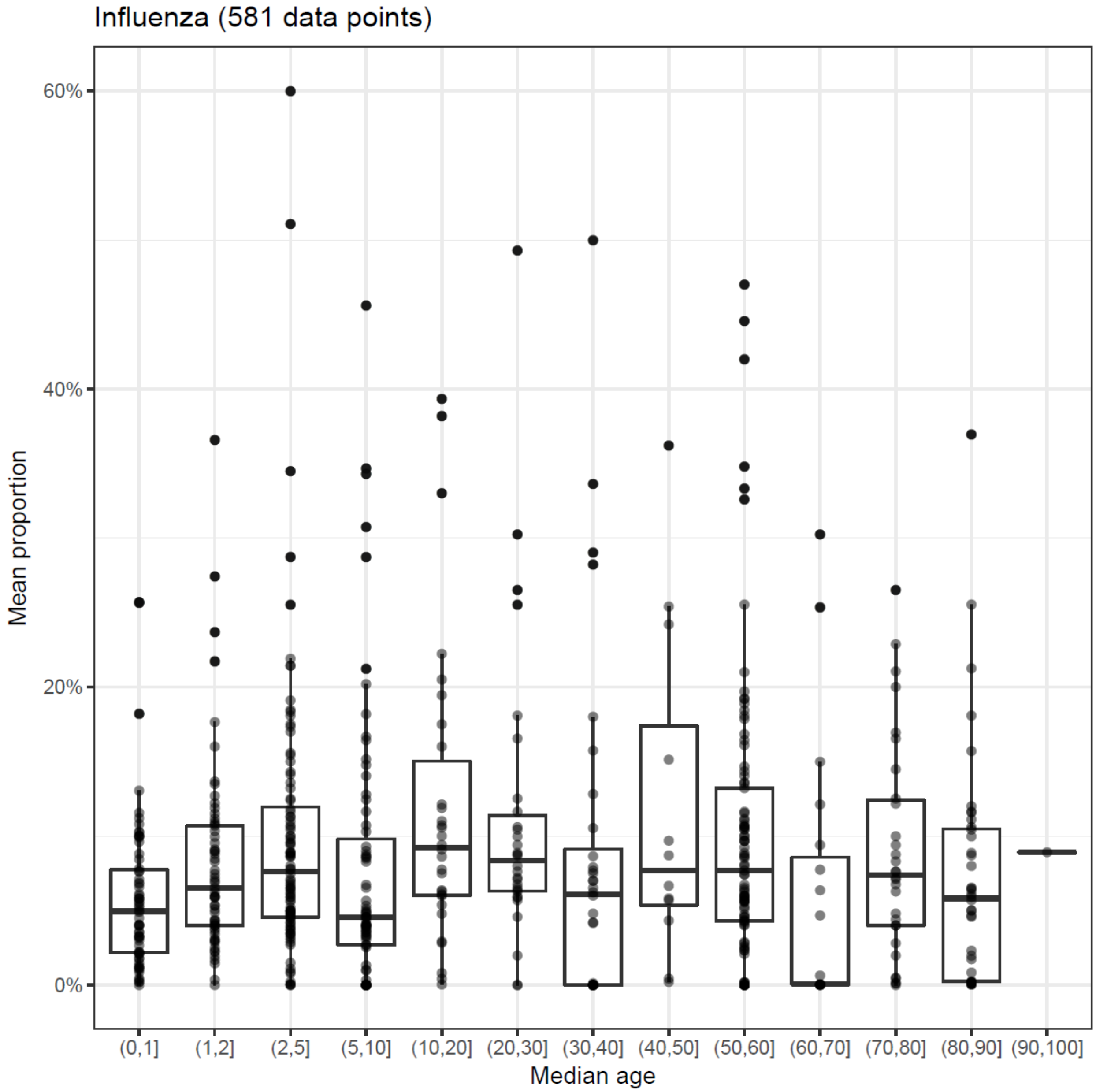

Influenza (581 data points)
Mean proportion
60%
40%
20%
0%
(0,1]
(1,2]
(2,5]
(5,10]
(10,20]
(20,30]
(30,40]
(40,50]
(50,60]
(60,70]
(70,80]
(80,90]
(90,100]
Median age

C)

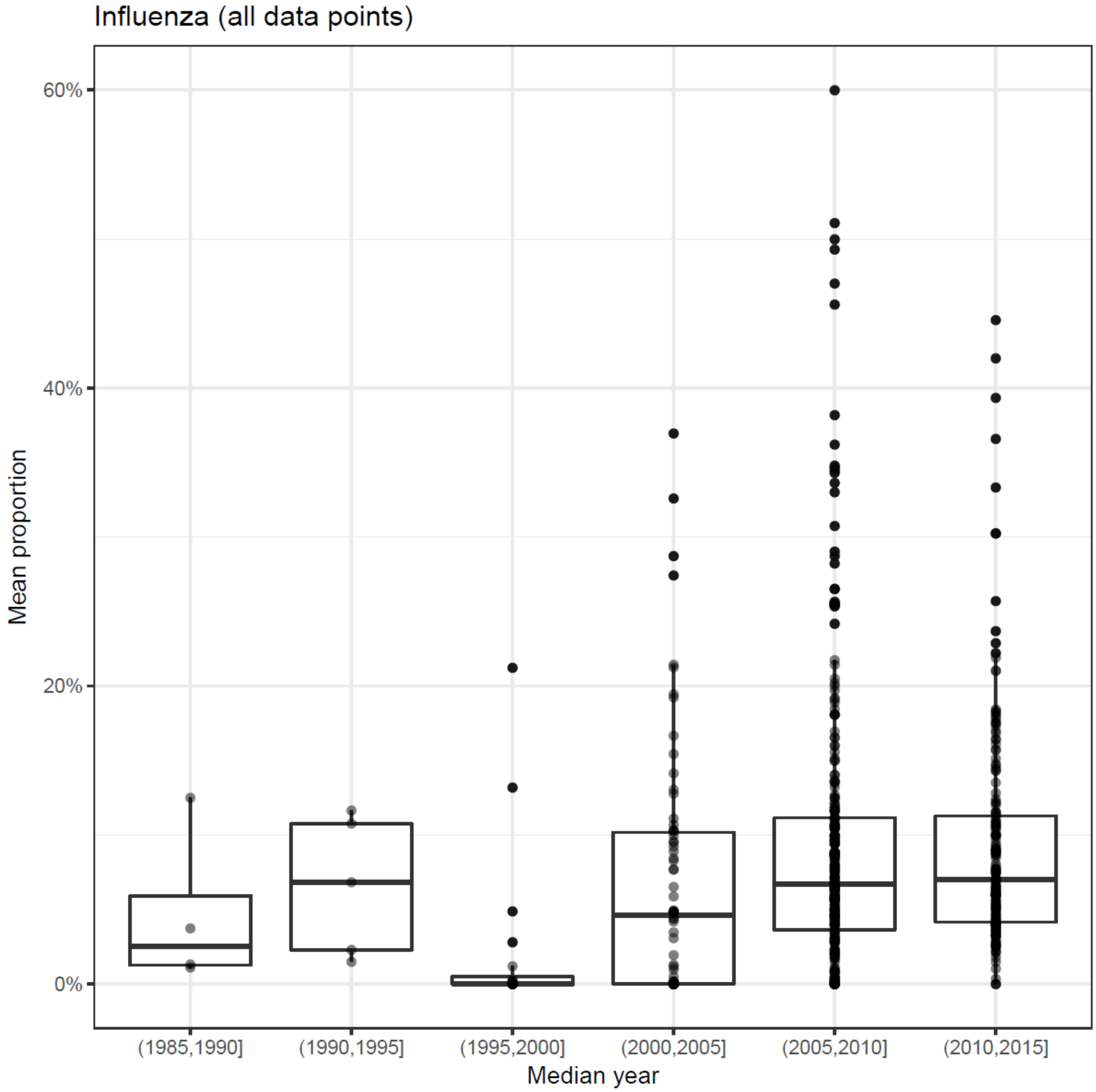
Influenza (all data points)
60%
40%
20%
0%
Mean proportion
(1985,1990]
(1990,1995]
(1995,2000]
(2000,2005]
(2005,2010]
(2010,2015]
Median year

Appendix Figure 4. Predicted ratio of mortality in viral to bacterial causes of LRI based on ICD-coded hospitalisations and deaths.

The transparency of the points corresponds to the uncertainty around each data point with darker points having smaller standard error estimates.

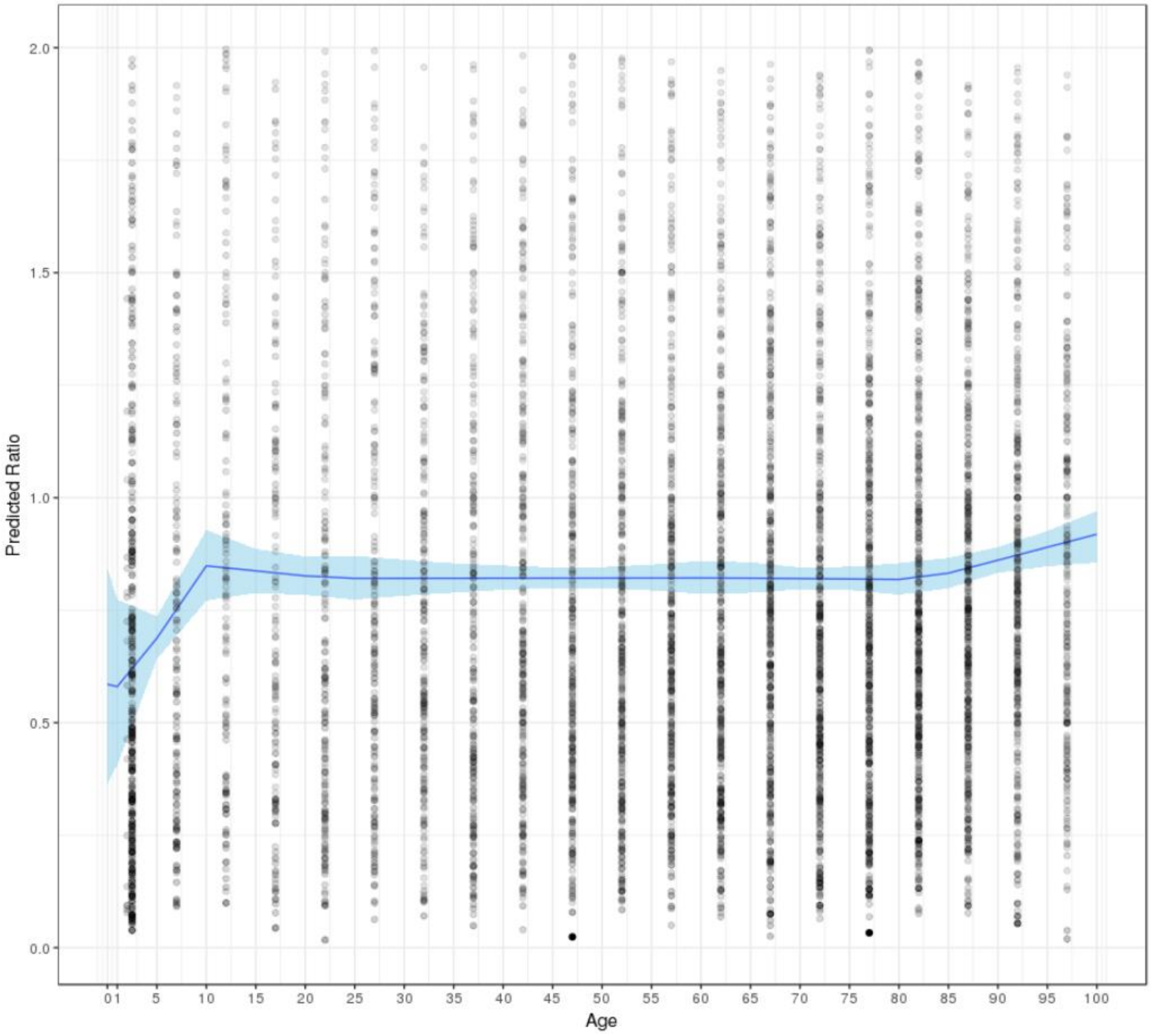

Appendix Table 4. Source list for LRI inpatient admissions data. These data sources are used in the LRI hospitalization model, the model that produces the total number of LRI hospitalisations. The product of the LRI hospitalisations estimates and the influenza hospitalisation PAF estimates is the hospitalised influenza LRI estimates.

Country	Name of database (series or system, or citation)
Austria	Austria Hospital Inpatient Discharges
Belgium	European Hospital Morbidity DataBase
Brazil	Brazil Hospital Information System (SIH)
Chile	Chile Hospital Discharge Information System
China	China Hospital Inpatient Discharges
Croatia	European Hospital Morbidity DataBase
Cyprus	European Hospital Morbidity DataBase
Czech Republic	European Hospital Morbidity DataBase
Denmark	European Hospital Morbidity DataBase
Ecuador	Ecuador Hospital Inpatient Discharges
Finland	European Hospital Morbidity DataBase
Georgia	Georgia Hospital Data
Germany	Germany Hospital Statistics Reporting System
Iceland	European Hospital Morbidity DataBase
India	India - Mysore JSS Hospital
India	India - Shillong Nazareth Hospital Inpatient Discharges
Indonesia	Indonesia Integrated Hospital Data
Iran	Iran Hospital Data
Italy	Italy - Hospital Inpatient Discharges
Japan	Japan Diagnosis Procedure Combination Database 2015
Jordan	Jordan Al-Bashir Hospital Discharges 2016
Kenya	Kenya National Inpatient Morbidity and Mortality Statistics
Kyrgyzstan	Kyrgyzstan - Bishkek Clinical-Related Groups Hospital Claims
Latvia	European Hospital Morbidity DataBase
Lithuania	European Hospital Morbidity DataBase
Luxembourg	European Hospital Morbidity DataBase
Malta	European Hospital Morbidity DataBase
Mexico	Mexico Automated Hospital Discharge System (SAEH)
Nepal	Nepal Hospital Inpatient Discharges
New Zealand	New Zealand National Minimum Dataset
Norway	Norway Patient Register
Philippines	Philippine Health Insurance Corporation Claims
Poland	European Hospital Morbidity DataBase
Portugal	Portugal Hospital Inpatient Discharges

Qatar	Qatar - Annual Inpatients Discharge Abstract: Hamad General Hospital
Romania	European Hospital Morbidity DataBase
Serbia	European Hospital Morbidity DataBase
Slovakia	European Hospital Morbidity DataBase
Slovenia	European Hospital Morbidity DataBase
Sweden	Sweden National Patient Register
Switzerland	European Hospital Morbidity DataBase
Taiwan	Taiwan National Health Insurance Claims
Turkey	Turkey Diagnosis-Related Group Hospital Inpatient Database
United Kingdom	United Kingdom - England Hospital Episode Statistics
United States	United States State Inpatient Databases
United States	United States National Hospital Discharge Survey
Vietnam	Vietnam Hospital Data

Supplementary results

Appendix Figure 5. Influenza LRI case fatality ratio (%), all ages in 2017.

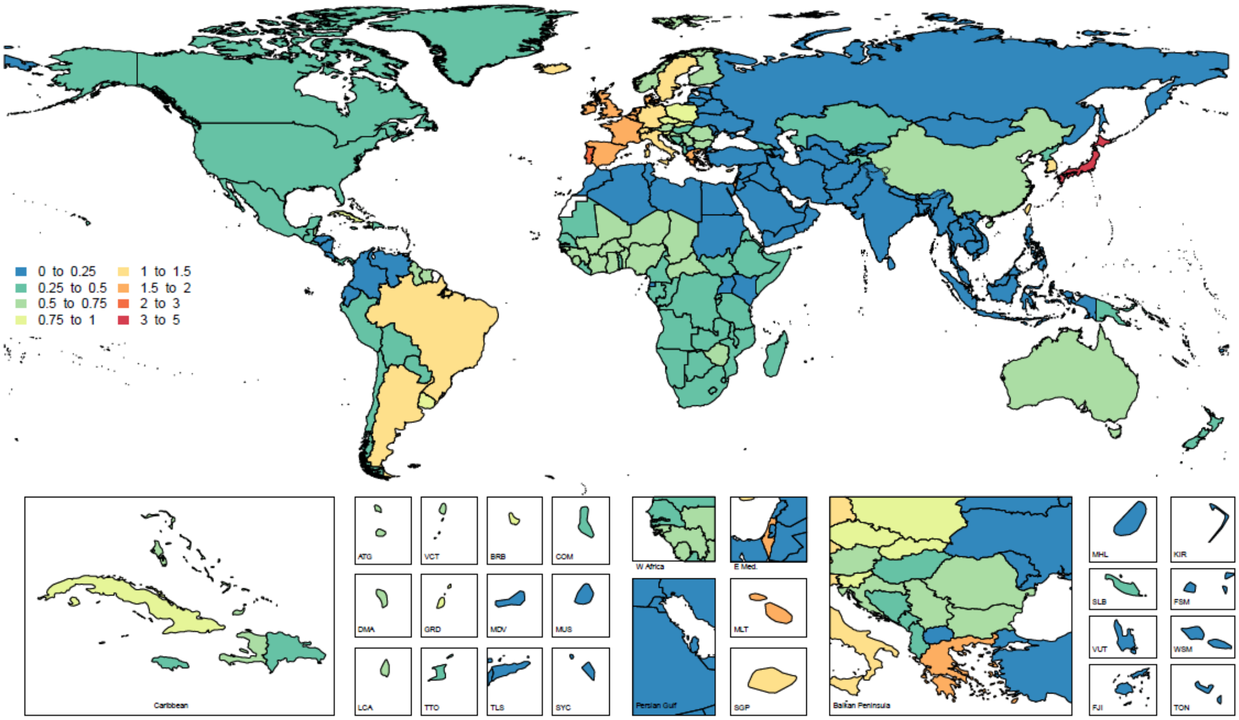

Appendix Figure 6. The influenza attributable fraction for LRI among all ages in 2017 for A) Episodes, B) Hospitalisations, and C) Deaths.

A)

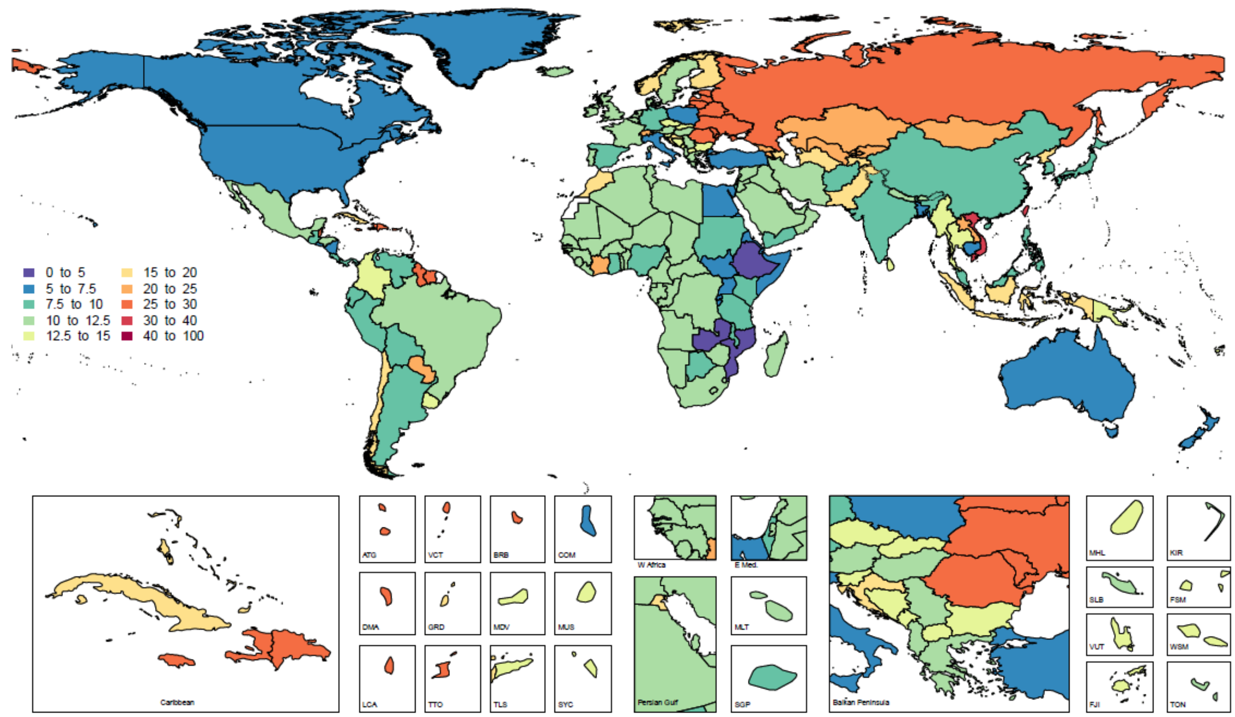

B)

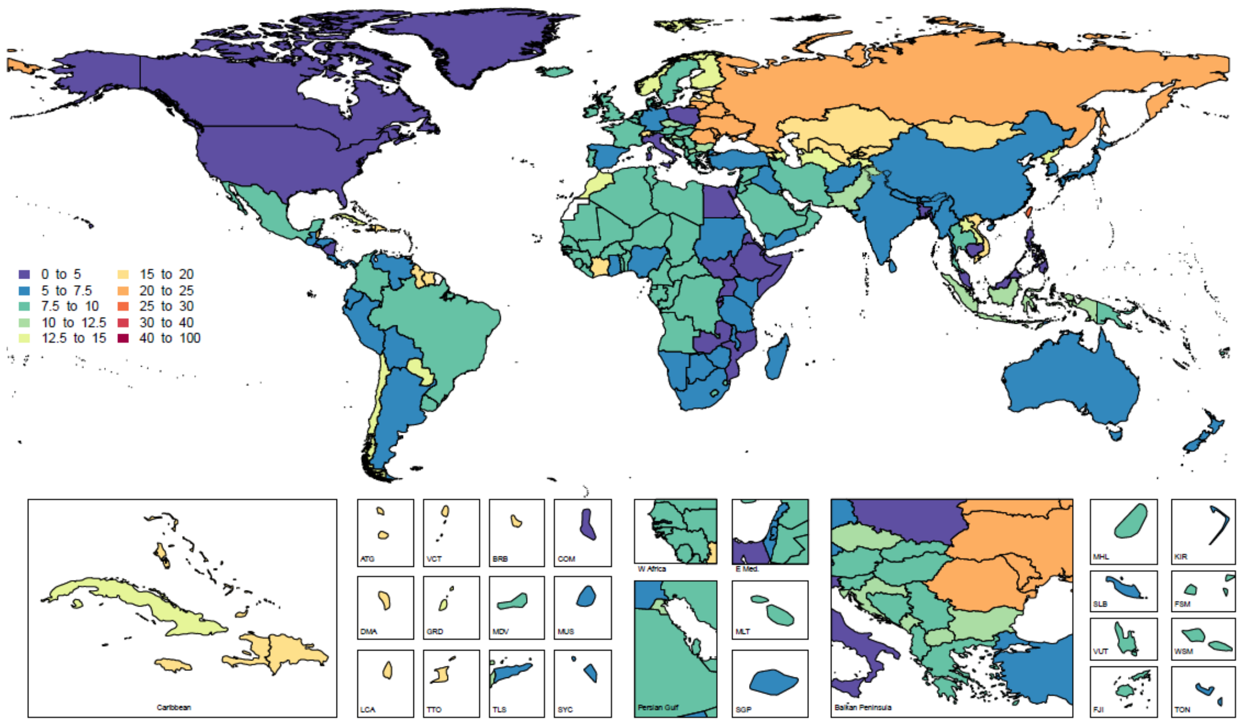

C)

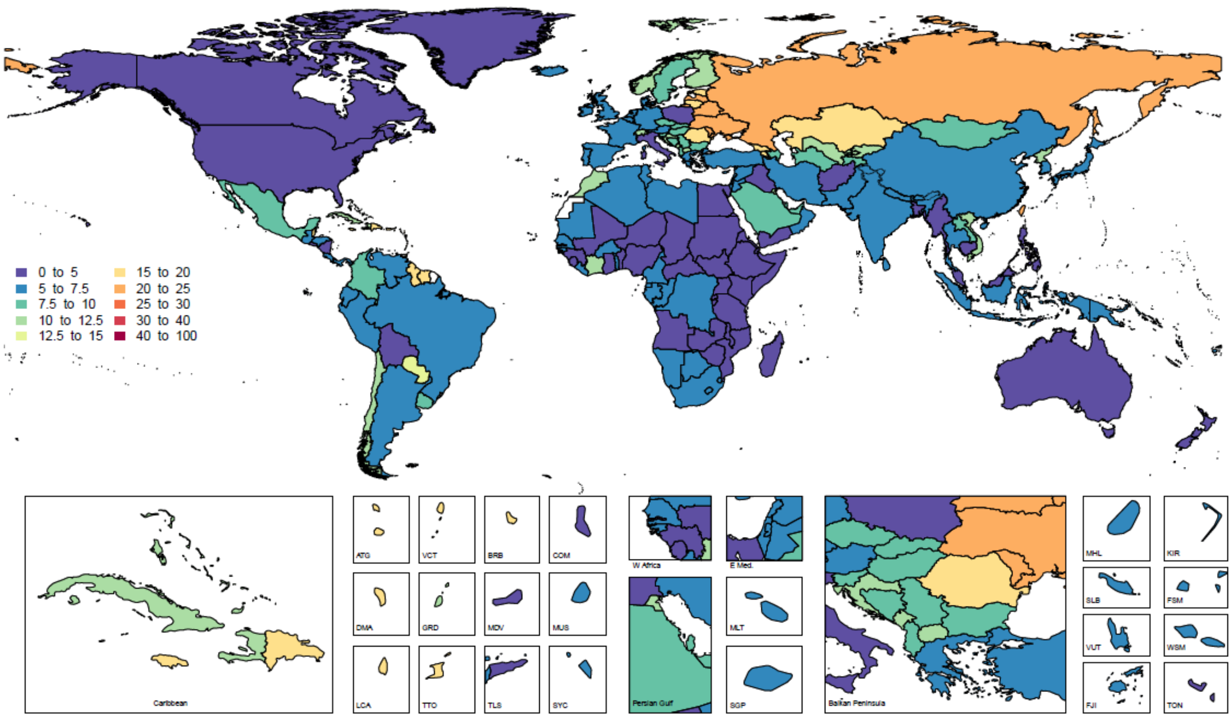

Appendix Figure 7. Proportion of influenza LRI episodes that were hospitalised in 2017.

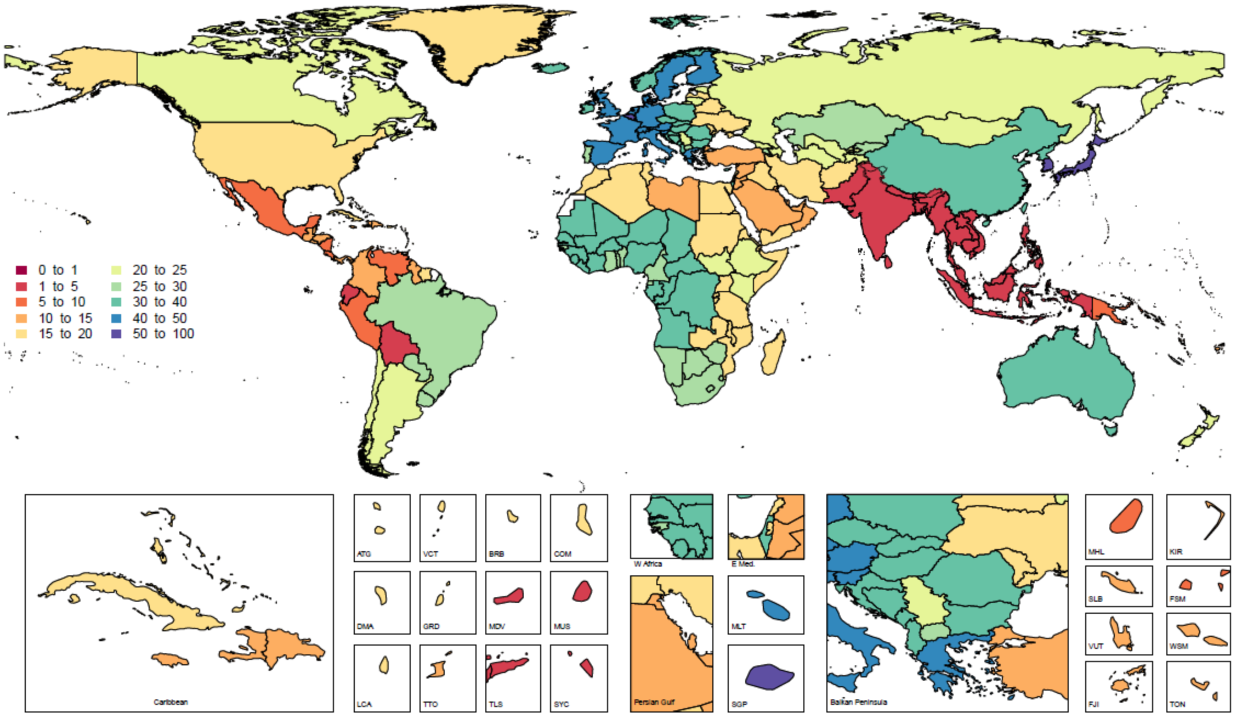

Appendix Figure 8. The influenza LRI case fatality ratio and proportion of influenza LRI episodes that were hospitalised in 2017.

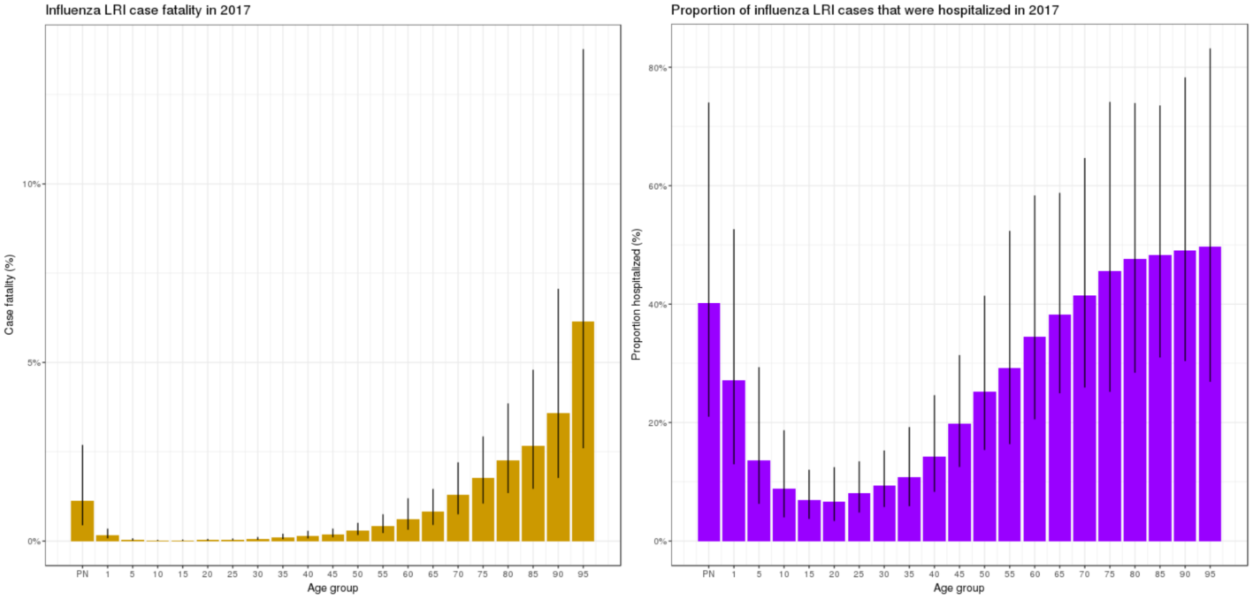

Comparison with GBD 2016

The attribution of influenza to fatal LRI episodes is much greater in GBD 2017 compared to GBD 2016.[8] The primary reason is due to a reanalysis of the ratio of mortality in viral compared to bacterial causes of LRI. We performed an age-integrating meta-regression of these values using hospital admissions records that were specific to the viral or bacterial cause of LRI among individuals for whom survival or mortality were known. We then created a ratio of the case fatality for viral causes of LRI and for bacterial causes of LRI and took the ratio of those estimates. For GBD 2017, we added substantially more data to this model including data from Austria, Brazil, Chile, China, Ecuador, Italy, Kenya, Mexico, New Zealand, Philippines, Portugal, and the United States. GBD 2016 data were limited to the USA, Brazil, Austria, and Mexico. The results of this re-analysis are shown in **Appendix Table 5 and** in scatter plots of the number of deaths and the attributable fraction comparing GBD 2016 and 2017 (**Appendix Figure 9**).

Appendix Table 5. Ratio of mortality in viral to bacterial causes of LRI comparing GBD 2016 and GBD 2017.

These values represent a scalar, so larger values indicate a more similar probability of death in viral and bacterial LRI and also indicate that the attributable fraction of LRI deaths due to influenza will be greater.

Age Group	Ratio GBD 2016	Ratio GBD 2017
Early Neonatal	0.34 (0.19-0.58)	0.59 (0.36-0.84)
Late Neonatal	0.34 (0.19-0.58)	0.58 (0.37-0.84)
Post Neonatal	0.34 (0.19-0.58)	0.58 (0.41-0.77)
1 to 4	0.28 (0.16-0.44)	0.69 (0.64-0.74)
5 to 9	0.31 (0.15-0.56)	0.85 (0.77-0.93)
10 to 14	0.33 (0.19-0.53)	0.84 (0.79-0.89)
15 to 19	0.37 (0.2-0.64)	0.83 (0.78-0.87)
20 to 24	0.46 (0.12-1.16)	0.82 (0.77-0.87)
25 to 29	0.44 (0.17-0.93)	0.82 (0.78-0.86)
30 to 34	0.46 (0.22-0.83)	0.82 (0.79-0.85)
35 to 39	0.5 (0.22-1)	0.82 (0.8-0.85)
40 to 44	0.61 (0.13-1.75)	0.82 (0.8-0.85)
45 to 49	0.5 (0.21-0.99)	0.82 (0.8-0.85)
50 to 54	0.44 (0.23-0.74)	0.82 (0.79-0.85)
55 to 59	0.42 (0.21-0.75)	0.82 (0.79-0.86)
60 to 64	0.42 (0.15-0.95)	0.82 (0.79-0.86)
65 to 69	0.39 (0.19-0.7)	0.82 (0.8-0.85)
70 to 74	0.38 (0.21-0.61)	0.82 (0.79-0.85)
75 to 79	0.37 (0.2-0.62)	0.82 (0.78-0.85)
80 to 84	0.37 (0.17-0.71)	0.83 (0.8-0.87)
85 to 89	0.37 (0.17-0.71)	0.86 (0.83-0.89)
90 to 94	0.37 (0.17-0.71)	0.89 (0.85-0.93)
95 to 99	0.37 (0.17-0.71)	0.92 (0.86-0.97)

Appendix Figure 9. A comparison of the number of deaths (A) and the attributable fraction (B) of influenza LRI between GBD 2016 and GBD 2017.

A)

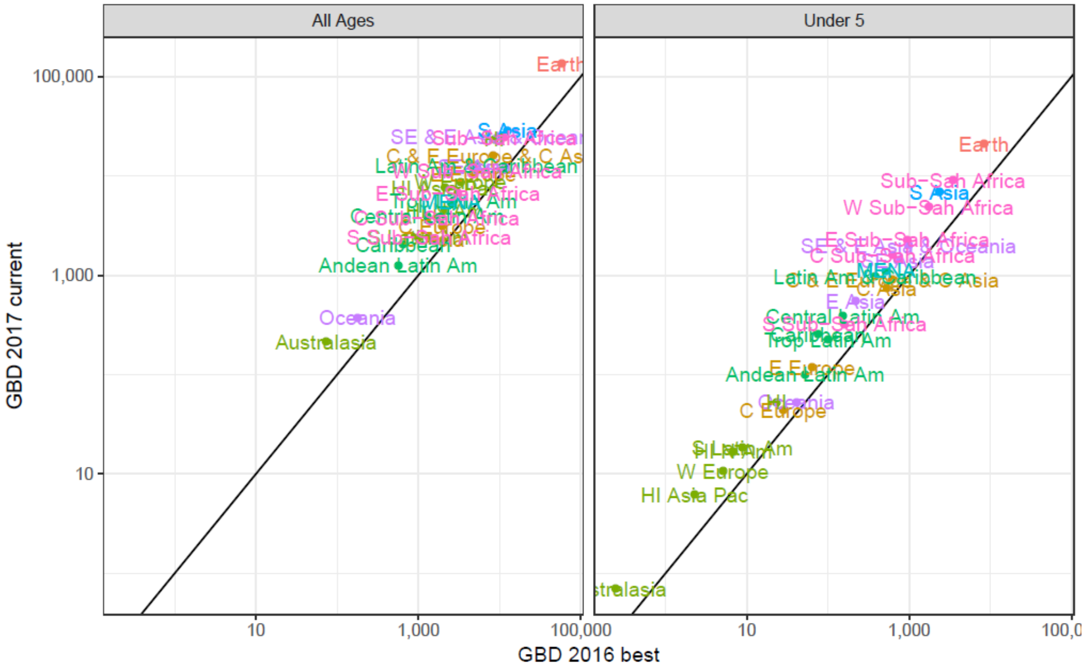

B)

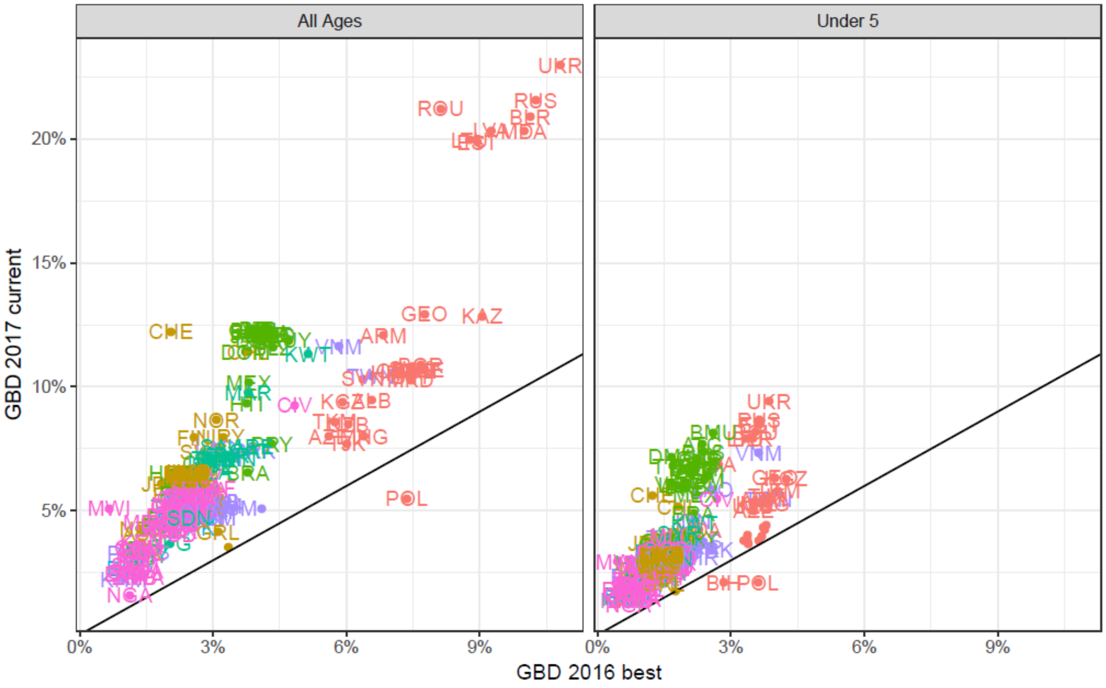

Contributors

C.T. and R.C.R. conducted the analyses for this manuscript. C.T. and R.C.R. prepared the first draft. C.T. constructed the figures and tables and prepared the appendix. A.H.M., C.L.J.M, S.I.H., and R.C.R. provided overall guidance. B.F.B, C.T., and I.A.K. finalised the manuscript based on comments from other authors and reviewer feedback. B.F.B. managed the project. All other authors provided data or developed models for indicators, reviewed results, initiated modelling infrastructure, and/ or reviewed and contributed to the report.

References

1 GBD 2015 Mortality and Causes of Death Collaborators. Global, regional, and national life expectancy, all-cause and cause-specific mortality for 249 causes of death, 1980–2015: a systematic analysis for the Global Burden of Disease Study 2015. *Lancet* 2016; **388**: 1459–544.

2 Foreman KJ, Lozano R, Lopez AD, Murray CJ. Modeling causes of death: an integrated approach using CODEm. *Popul Health Metr* 2012; **10**: 1.

3 Foreman KJ, Lozano R, Lopez AD, Murray CJ. Modeling causes of death: an integrated approach using CODEm. *Popul Health Metr* 2012; **10**: 1.

4 GBD 2016 Mortality Collaborators. Global, regional, and national under-5 mortality, adult mortality, age-specific mortality, and life expectancy, 1970-2016: a systematic analysis for the Global Burden of Disease Study 2016. *Lancet Lond Engl* 2017; **390**: 1084–150.

5 World Health Organization: Department of Child and Adolescent Health and Development. Handbook Integrated Management of Childhood Illness. 2005.

6 GBD 2016 Causes of Death Collaborators. Global, regional, and national age-sex specific mortality for 264 causes of death, 1980-2016: a systematic analysis for the Global Burden of Disease Study 2016. *Lancet* 2017; **390**: 1151–210.

7 GBD 2016 Disease and Injury Incidence and Prevalence Collaborators. Global, regional, and national incidence, prevalence, and years lived with disability for 328 diseases and injuries for 195 countries, 1990-2016: a systematic analysis for the Global Burden of Disease Study 2016. *Lancet* 2017; **390**: 1211–59.

8 GBD 2016 LRI Collaborators. Estimates of the global, regional, and national morbidity, mortality, and aetiologies of lower respiratory tract infections in 195 countries: a systematic analysis for the Global Burden of Disease Study 2016. *LANCET Infect Dis* 2018; **In process**.

9 Shi T, McLean K, Campbell H, Nair H. Aetiological role of common respiratory viruses in acute lower respiratory infections in children under five years: A systematic review and meta-analysis. *J Glob Health* 2015; **5**: 010408.
